# Variation of Long Non-Coding RNA And mRNA Profiles in Breast Cancer Cells With Influences of Adipocytes

**DOI:** 10.3389/fonc.2021.631551

**Published:** 2021-05-21

**Authors:** Xin-Hui Cao, Kai Yang, Ming-Xing Liang, Pei Ma, Di Xu, Yin-Jiao Fei, Wei Zhang, Xiu Chen, Jin-Hai Tang

**Affiliations:** ^1^ School of Clinical Medicine, Xuzhou Medical University, Xuzhou, China; ^2^ Department of General Surgery, The First Affiliated Hospital of Nanjing Medical University, Nanjing, China; ^3^ Department of Oncology, The First Affiliated Hospital of Nanjing Medical University, Nanjing, China

**Keywords:** breast cancer, long-non-coding RNA, adipocytes, tumor microenvironment, mRNA

## Abstract

**Background:**

It is well known that obesity is one of the risks for incurrence and development in breast cancer patients. Long non-coding RNAs (lncRNAs) are reported to participate in the composition of tumor microenvironment and to regulate breast cancer cell metabolic activities. However, there was rare study focused on the lncRNAs in breast cancer with the influences of adipocytes. The study aimed to investigate lncRNAs expression profiles and discover potential biomarkers to predict the incidence and progression of adipocyte-associated-breast cancer.

**Methods:**

We co-cultured adipocytes with breast cancer cells and profiled the expression of lncRNAs as well as mRNAs by using the RNA-sequencing method. Wound Healing, Migration assays and Invasion assays were applied to verify the invasion and metastasis of cancer cells.

**Results:**

MDA-MB-231/Hpa-V and SK-BR-3/Hpa-V cells showed elevated migration and invasiveness compared to the control group. A sum of 371 mRNAs (181 upregulated and 190 downregulated) and 850 lncRNAs(414 upregulated and 436 downregulated) were differentially expressed in MDA-MB-231/Hpa-V comparing to MDA-MB-231(P < 0.05; |log2 (fold change)|>1.2). GO enrichment, KEGG pathway and interaction networks demonstrated that differentially expressed lncRNAs were involved in functional categories, such as material metabolism, which might lead to the progression of breast cancer.

**Conclusion:**

Our study detected a lncRNA profile in breast cancer cells affecting by adipocytes and provided a better understanding of the tumor microenvironment. LncRNAs may be helpful to predict the therapeutic responses and prognosis of obese breast cancer patients.

## Backgrounds

The breast cancer remains to be the leading cause of cancer incidences and mortality in United States women, with 276,480 estimated new cases and 42,170 estimated deaths in 2020 (1). Nevertheless, the progress against breast cancer is slowing since 2004 partly attributed to early detection through screening, continued decline in the fertility rate as well as decreased obesity (2).

Obesity is one well-known risk for breast cancer, meanwhile, adipose tissue accounts for almost 30% of body mass in obese women. In the obese population, the crosstalk between cancer cells and adipocytes have the potency to alter the tumor microenvironment, which may explain the alterations of adipocytes in breast cancer development (3). The adipose tissue consists of subcutaneous adipose tissue and visceral adipose tissue, and researchers have reported that the obesity-driven dysfunction of visceral adipose tissue played a major role in the metastasis progress in breast cancer (4). Besides, growing evidence have suggested that abdominal obesity, namely the excess visceral adipose tissue might increase risks for triple negative breast cancer (5).

As far as we know, long non-coding RNAs (lncRNAs) possess more than 200 nucleotides, including intronic, antisense, long intervening non-coding RNAs (lincRNA), competing endogenous RNA (ceRNA) and so on (6). LncRNAs were used to be considered as transcriptional noise, while after considerable researches, lncRNAs are detected to be vital elements in tumor microenvironment, although with a little enigmatic understanding (7). Until now, lncRNAs are reported to modulate breast cancer cell metabolic activities, such as cell proliferation, metastasis, invasion, drug resistance and some other behaviors (8). Besides, the most frequent ways of lncRNAs to regulate cancer biological processes are the cross-talking with microRNAs(miRNAs), sponging miRNAs and the regulation of their expressions namely the lncRNA-miRNA-mRNA network (9, 10).

Also, experts have already discovered precise mechanisms for the effect of obesity or adipocytes on lncRNA expressions (11, 12). Similarly, recent studies have found that lncRNA LINC00689 was associated with obesity and eukaryotic gene expressions (13). However, differential expression profiles of lncRNAs in breast cancer cells with the adipocyte’s influences are unknown.

Therefore, we co-cultured adipocytes and breast cancer cells and analyzed the changes of lncRNAs in breast cancer cells, which might be helpful to find out potential biomarkers to predict the incidence and progression of adipocyte-associated-breast cancer.

## Methods

### Cell Lines

Human breast cancer cell line MDA-MB-231 and SK-BR-3(SkBr3) was obtained from the Cell Bank of the Chinese Academy of Sciences (Shanghai, China) and the human preadipocyte-visceral Hpa-V was bought from Cellbio Company (Shanghai, China). MDA-MB-231, SkBr3 and Hpa-V cells were both cultured in Dulbecco’s modified Eagle’s medium (DMEM) high glucose (HyClone), supplemented with 10% fetal bovine serum (FBS), and 1%-1.5% penicillin with streptomycin (Cellbio). Both of the cells were incubated at 37°C and 5% CO2 atmosphere.

### Cell Co-Culture Experiment

About 4×10^4^ Hpa-V cells were seeded in the upper chamber of transwell assay inserts (pore size of 0.4 mm; Corning) with 1mL serum-free DMEM medium, while 5×10^5^ MDA-MB-231 or SkBr3 cells with DMEM medium containing 10% FBS were added into the lower chamber by a 6-well transwell chamber. The co-cultured MDA-MB-231 or SkBr3 cells were named MDA-MB-231/Hpa-V or SkBr3/Hpa-V. After 48 hours, MDA-MB-231/Hpa-V, SkBr3/Hpa-V and MDA-MB-231, SkBr3 cells on the lower chamber were collected to perform the following experiments.

### Wound Healing

Firstly, MDA-MB-231/Hpa-V, SkBr3/Hpa-V, MDA-MB-231 and SkBr3 cells in 6-well plates were cultured to 90-95% confluency. Then a linear wound was scraped by a sterile 200μl Pipette head across the confluent cell layer. Cells were washed thrice by PBS to remove floated cells and debris. After pictured instantly at 0 hour, cells were cultured as mentioned above for another 24 hours and were pictured again. The widths of the wounds were photographed by the camera (Canon, Japan). The ratio of the cell migration area to the initial area represents the migration ability of cells under different treatments. The healing area(%) was calculated as (initial 0h area - residual 24h area)/initial 0h area*100%. All experiments were repeated three times.

### Migration Assays

The transwell experiment was used to verify the effect of Hpa-V cells on the transfer ability of MDA-MB-231 or SkBr3 cells. Firstly, cells were digested with trypsin, resuspended in a serum-free medium in the co-culture group and the control group. About 4×10^4^ cells were seeded in the upper chamber of transwell assay inserts (pore size of 8 mm; Corning) with 200μL serum-free DMEM medium, and 600μL DMEM medium containing 20% FBS was added into the lower chamber by a 24-well transwell chamber. After 24 hours of incubation at 37°C, the chamber was fixed with 4% paraformaldehyde (Servicebio),stained with 1% crystal violet (Sangon biotech) and washed softly by PBS. Next, the cells inside the chamber were gently wiped with a cotton swab. Until the chamber was dried, we took pictures with a microscope (Carl ZEISS, USA). All experiments were repeated thrice.

### Invasion Assays

Before seeding cells in the upper transwell chamber (pore size of 8 mm; Corning), 100μL mixture (1:8) of Matrigel (Corning, NY, USA) and serum-free DMEM medium (Kaiji, Nanjing, China) were coated on the upper transwell chamber. After 1h for Matrigel concretion, followed steps were similar to migration assays. All experiments were performed triplicated.

### Sequencing of lncRNA and mRNA Expression Profiles

5×10^5^ MDA-MB-231/Hpa-V and MDA-MB-231 cells were seeded in a 6-well plate separately. Total RNA was extracted from breast cancer cells and cocultured breast cancer cells by using a miRNeasy Mini Kit (Qiagen, Hilden, Germany) following the manufacturer’s instructions. The RNA concentrations and purities were measured at 260/280 nm using a NanoDrop 2000 spectrophotometer (Thermo Electron Corporation, USA) and by Bioanalyzer 4200 (Agilent, Santa Clara, CA, USA). The NGS libraries were prepared using HISAT2 for Illumina^®^ (Vazyme, Nanjing, China) and obtained most of the transcripts in this state. The sequenced reads were then compared to the reference genomes, and the genes or transcripts were annotated and quantified.

### Differential Expressions of mRNAs and lncRNAs

The mRNAs and lncRNAs with a P value<0.05 and |log2 (fold change)|>1.2 were selected and considered differentially expressed between the compared groups by analysis of DEseq2.

### Validation by RT-qPCR

Reverse transcription and quantitative real-time PCR (RT-qPCR) were performed to affirm the sequencing results. A total of 500ng of RNA was reversely transcribed *via* using HiScript II Q RT SuperMix for qPCR (Vazyme) for cDNA synthesis, and ChamQ SYBR qPCR Master Mix (High ROX Premixed) (Vazyme) was used to perform PCR on StepOnePlus Real-Time PCR System (Thermo Fisher Scientific, USA). The amplication procedure was set as 95°C 3min following 40 cycles of 95°C 10s and 60°C 30s. Then the melting curve was complicated as 15s at 95°C, 1min at 60°C, 15s at 95°C. The relative expressions of lncRNAs were calculated by using the 2^−ΔΔCt^ method with the internal reference being β-actin.

### Gene Ontology and KEGG Pathway Analysis

Differentially expressed mRNAs were further analyzed with Gene Ontology (GO) enrichment analysis (http://www.geneontology.org) and the Kyoto Encyclopedia of Genes and Genomes (KEGG) pathway database (http://www.genome.jp/kegg) to investigate their functions. P value<0.05 was considered statistically significant.

### Co-Expression Network

The interactions between lncRNAs and mRNAs were constructed as a coding-noncoding gene co-expression (CNC) network with Cytoscape (version 3.7.2) software. And the mRNAs correlated with lncRNAs were calculated through enrichment analysis.

### Statistical Analysis

We used student’s t-test and chi-squared test analysis to compare continuous variables as mean ± SD and categorical variables as percentages and numbers, respectively by using IBM SPSS Statistics 25.0 or GraphPad Prism 8.0.1. When we were conducting RNA-sequencing analysis, such as hierarchical clustering, GO enrichment, KEGG pathway analysis and co-expression network, we applied the R package with the above mentioned analysis. GraphPad Prism 6.0 and Adobe Photoshop CC were utilized to draw graphs. P < 0.05 was considered to be statistically significant.

## Results

### Hpa-V Cells Improve the Migration of Breast Cancer Cells

**Figure 1:**
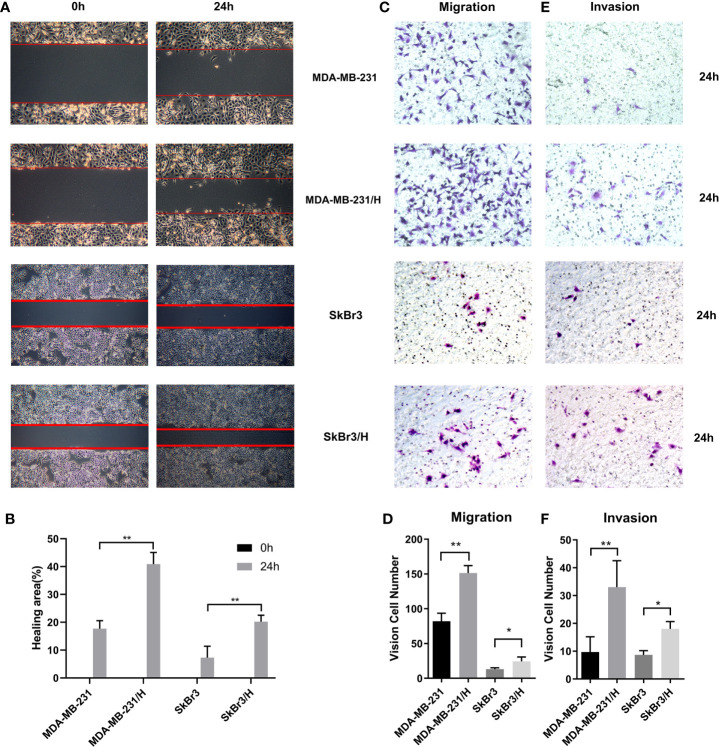
Adipocytes promote invasion and migration of breast cancer cells. **(A)** wound healing experiment of the MDA-MB-231 or SkBr3 and Hpa-V co-cultured group and the control group (40 magnification); **(B)** a histogram shows the detailed healing area of the wound healing experiment; **(C)** transwell migration tests of co-cultured group and the control(200 magnification); **(D)** a histogram shows the specific vision cell number of transwell migration tests; **(E)** matrigel invasion tests of co-cultured group and the control(200 magnification); **(F)** a histogram shows the specific invaded cell number of matrigel invasion tests. MDA-MB-231/H: MDA-MB-231/Hpa-V; SkBr3/H: SkBr3/Hpa-V. **P < 0.01, *P < 0.05. The experiments were performed in triplicate.

A fixed number of adipocyte Hpa-V was co-cultured with MDA-MB-231 or SkBr3 breast cancer cells for 48 hours. Namely, we used 4*10^4^/5*10^5^ as the Hpa-V/breast cancer cells proportion in the following studies. Wound healing (**Figures 1A, B**) and transwell migration tests (**Figures 1C, D**) on MDA-MB-231/Hpa-V and SkBr3/Hpa-V cells showed that the migration ability of MDA-MB-231/Hpa-V or SkBr3/Hpa-V cells were enhanced compared with the control MDA-MB-231 or SkBr3 group. Matrigel invasion experiments concluded that MDA-MB-231/Hpa-V or SkBr3/Hpa-V cells showed increased invasiveness compared with the control group (**Figures 1E, F**).

### The Landscape of mRNAs and lncRNAs Expression of Breast Cancer Cells

**Figure 2:**
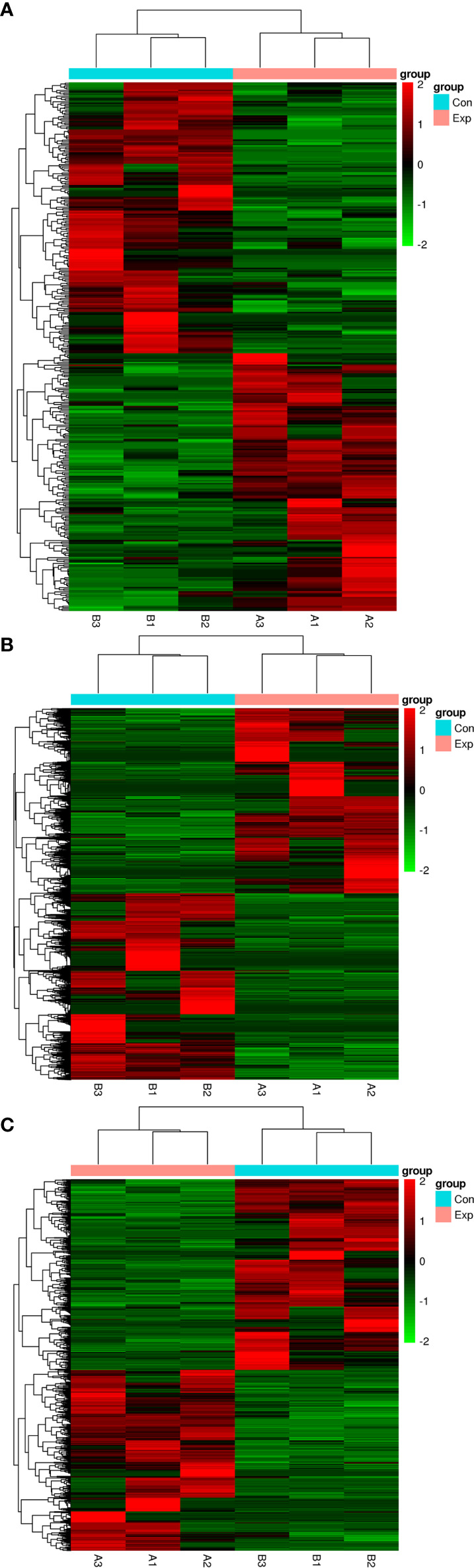
The hierarchical clustering heatmaps of differential mRNAs **(A)**, transcripts **(B)** and lncRNAs **(C)** after filtered by DEseq2. (P < 0.05, |log2 (fold change)|>1.2). The red color means upregulated, while the green color means downregulated. Exp: the experimental group, namely the MDA-MB-231/Hpa-V co-cultured group and the three samples were listed as A1, A2, A3. Con: the control group, namely the MDA-MB-231 group and the three samples were listed as B1, B2, B3.

**Figure 3:**
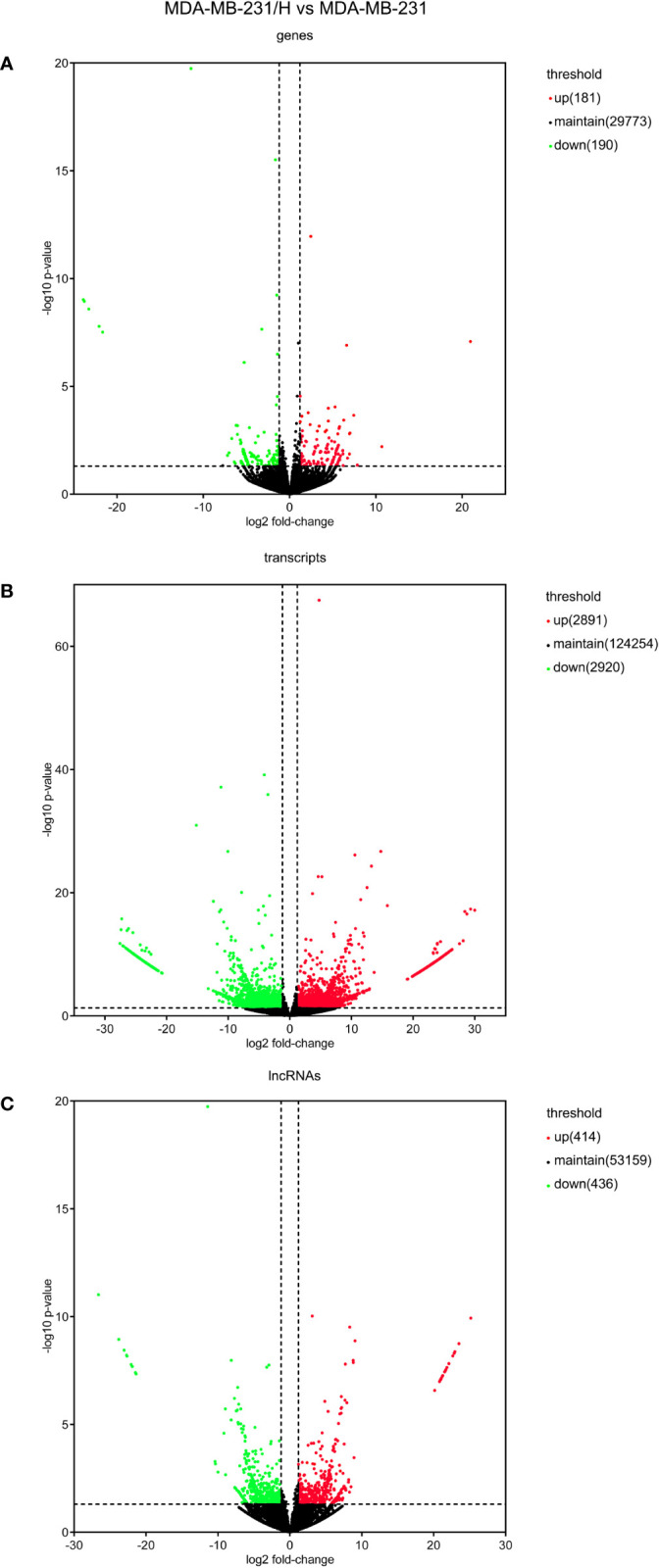
The volcano plots of the differentially expressed mRNAs **(A)**, transcripts **(B)** and lncRNAs **(C)**. (P < 0.05; |log2 (fold change)|>1.2). The red dots mean upregulated, while the green dots mean downregulated, and the black dots mean no differential expressions.

**Figure 4:**
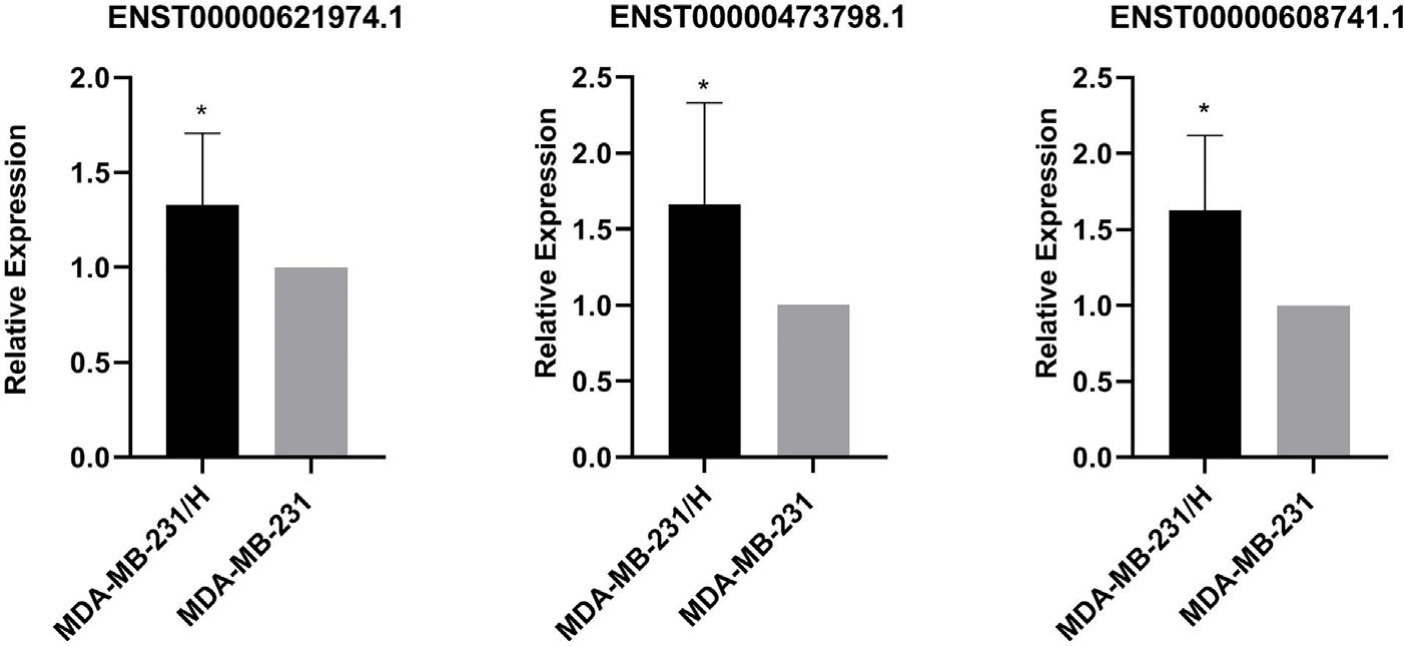
Verification of 3 differentially expressed lncRNAs(ENST00000621974.1, ENST00000473798.1, ENST00000608741.1). The relative expressions of ENST00000621974.1, ENST00000473798.1 and ENST00000608741.1 were significantly increased in MDA-MB-231/Hpa-V in comparison with MDA-MB-231 cells. MDA-MB-231/H: MDA-MB-231/Hpa-V. *P<0.05. All experiment was performed in triplicate.

We performed transcripts and genes differential expression analysis between MDA-MB-231/Hpa -V and MDA-MB-231 cells as shown in **Figure 2** and **Tables S1–3**. To be more precise, there were 181 upregulated mRNA and 190 downregulated mRNA in MDA-MB-231/Hpa-V and MDA-MB-231 cells after filtering (P < 0.05; |log2 (fold change)|>1.2, **Figure 3A**, **Table S1**). And **Table 1** listed the top 30 differential genes. A total of 5,811 differential transcripts, including 3,407 protein-coding pattern, corresponding to 372 genes in MDA-MB-231/Hpa-V and MDA-MB-231 cells (P < 0.05; |log2 (fold change)|>1.2, **Figure 3B**, **Table S2**). In details, a sum of 850 lncRNAs were differentially expressed. Among them, 414 lncRNAs were upregulated in MDA-MB-231/Hpa-V comparing to MDA-MB-231, while 436 were downregulated (P < 0.05; |log2 (fold change)|>1.2, **Figure 3C**, **Table S3**). After filtering the results with the condition as length <2000bp, we found that in the rest lncRNAs, there were 61 lncRNAs upregulated in MDA-MB-231/Hpa-V while 69 downregulated. Among them, the top 30 differential lncRNAs were displayed in **Table 2**. In order to validate the sequencing outcomes, 3 lncRNAs were randomly selected to be examined by RT-qPCR. The results were consistent with the sequencing (**Figure 4**).

**Table 1:** Top 30 differential genes in MDA-MB-231/H vs MDA-MB-231 cells.

gene ID	gene name	chromosome	strand	fold change	p value	style
ENSG00000256349.1	AP002748.4	11	+	2033484.32424	0.00000	up
ENSG00000249839.1	AC011330.1	15	–	1636.89652	0.00628	up
ENSG00000217576.7	AL158066.1	13	–	230.02132	0.04475	up
ENSG00000270181.2	BIVM-ERCC5	13	+	174.01852	0.00022	up
ENSG00000283149.1	AC068631.2	3	+	128.54384	0.00146	up
ENSG00000272162.1	AL024498.2	6	+	123.57337	0.01366	up
ENSG00000270249.1	AC093668.1	7	–	122.66286	0.00157	up
ENSG00000240180.1	RPL12P28	10	–	104.76417	0.02126	up
ENSG00000210825.1	SNORA40	11	–	97.62974	0.00000	up
ENSG00000275558.1	RN7SKP175	8	+	77.51600	0.00037	up
ENSG00000272741.1	AC069257.3	3	–	73.86756	0.04670	up
ENSG00000124749.16	COL21A1	6	–	73.40452	0.01354	up
ENSG00000241326.1	AL603962.1	1	+	72.10779	0.00930	up
ENSG00000248101.2	AC002116.1	19	–	70.82991	0.00962	up
ENSG00000258572.1	AL133467.1	14	+	60.11768	0.01645	up
ENSG00000207501.1	RNVU1-14	1	+	0.00000	0.00000	down
ENSG00000276027.1	RNU12	22	+	0.00000	0.00000	down
ENSG00000273217.1	AC008695.1	5	–	0.00000	0.00000	down
ENSG00000273088.1	AL713999.1	1	–	0.00000	0.00000	down
ENSG00000196826.7	AC008758.1	19	–	0.00000	0.00000	down
ENSG00000275325.4	PDCD6IPP1	15	+	0.00000	0.00000	down
ENSG00000276612.3	FP565260.2	21	–	0.00036	0.00000	down
ENSG00000248710.1	AC079594.2	3	–	0.00466	0.04753	down
ENSG00000270299.1	AL121758.1	20	–	0.00679	0.01600	down
ENSG00000264668.1	AC138696.1	8	+	0.00771	0.01203	down
ENSG00000273628.1	AL354798.1	13	+	0.00962	0.00260	down
ENSG00000268412.2	TRMT112P6	2	–	0.01175	0.03185	down
ENSG00000284337.1	AC013271.1	2	+	0.01273	0.03769	down
ENSG00000196796.5	NPIPB10P	16	+	0.01367	0.00064	down
ENSG00000125695.12	AC046185.1	17	–	0.01514	0.00169	down

**Table 2:** Top 30 differential lncRNAs in MDA-MB-231/H vs MDA-MB-231 cells.

lncRNA name	type	length	gene name	fold change	p value	style
ENST00000526233.5	processed transcript	861	GPAA1	3373296.7008	0.0000	up
ENST00000522156.1	processed transcript	795	RPL30	446.0222	0.0000	up
ENST00000589598.5	processed transcript	769	PEPD	112.6651	0.0245	up
ENST00000630374.1	processed transcript	1977	SMIM14	85.7116	0.0000	up
ENST00000587299.1	processed transcript	1403	ATP6V0A1	74.0519	0.0341	up
ENST00000413148.1	sense intronic	1428	AL603962.1	73.3357	0.0318	up
ENST00000517898.1	antisense RNA	1370	AC090152.1	72.2292	0.0109	up
ENST00000555132.5	processed transcript	1772	CALM1	68.9368	0.0371	up
ENST00000609983.1	lincRNA	1801	MAPKAPK5-AS1	64.1641	0.0388	up
ENST00000554161.1	lincRNA	1776	AL133467.1	60.0657	0.0423	up
ENST00000556225.1	antisense RNA	1500	PSMA3-AS1	60.0190	0.0481	up
ENST00000471864.1	antisense RNA	1993	FAM201A	56.2623	0.0465	up
ENST00000559458.2	3prime overlapping ncRNA	1150	AL662884.1	54.2053	0.0062	up
ENST00000576943.1	antisense RNA	1978	ZNF213-AS1	43.9761	0.0016	up
ENST00000572222.1	antisense RNA	1225	MMP25-AS1	42.6586	0.0014	up
ENST00000477686.1	processed transcript	987	MRPL20	0.0020	0.0021	down
ENST00000496602.1	processed transcript	856	XIAP	0.0057	0.0000	down
ENST00000594572.1	processed transcript	979	PPFIA4	0.0061	0.0112	down
ENST00000588226.5	antisense RNA	1225	PARD6G-AS1	0.0068	0.0000	down
ENST00000556647.1	processed transcript	1558	SRSF5	0.0068	0.0122	down
ENST00000529498.1	processed transcript	1966	SPA17	0.0101	0.0415	down
ENST00000497786.1	processed transcript	717	TCTA	0.0102	0.0000	down
ENST00000562549.2	lincRNA	1583	AC092368.3	0.0113	0.0123	down
ENST00000510072.1	processed transcript	1113	TRIM41	0.0114	0.0013	down
ENST00000513851.1	lincRNA	575	AC108062.1	0.0128	0.0002	down
ENST00000491687.1	processed transcript	1935	PIGO	0.0144	0.0372	down
ENST00000563904.1	processed transcript	1269	HERC2P4	0.0169	0.0002	down
ENST00000563508.1	processed transcript	910	ZSCAN29	0.0170	0.0151	down
ENST00000482692.1	processed transcript	1378	CREB5	0.0172	0.0485	down
ENST00000507770.1	sense intronic	989	RASA2-IT1	0.0204	0.0021	down

**Figure 5:**
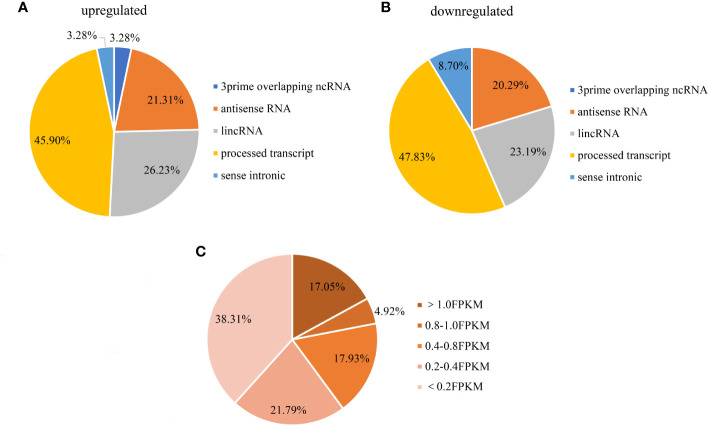
Distribution of differential lncRNAs (**A**: upregulated, **B**: downregulated) in breast cancer cells. **(C)** Distribution of differential lncRNAs abundance(shorter than 2000bp). **(A)** among the upregulated lncRNAs shorter than 2000bp, 45.90% were processed transcripts, 26.23% were lincRNAs and 21.31% were antisense RNAs; **(B)** among the downregulated lncRNAs shorter than 2000bp, 47.83% were processed transcripts, 23.19% were lincRNAs and 20.29% were antisense RNAs; **(C)** 39.9% of the lncRNAs FPKM expression were over 0.4, and 17.05% were over 1.

As we all know, the lncRNAs can be categorized into six types: 3prime overlapping ncRNA, antisense RNA, lincRNA, processed transcript, sense intronic. A large quantity of differentially expressed lncRNAs shorter than 2000bp was processed transcript, with antisense RNA and lincRNA made up for the majority of the rest (**Figures 5A, B**). Moreover, expressions of these lncRNAs were relatively high across all samples (almost 39.9% had an FPKM >0.4) (**Figure 5C**).

### GO and KEGG Pathway Analysis

**Figure 6:**
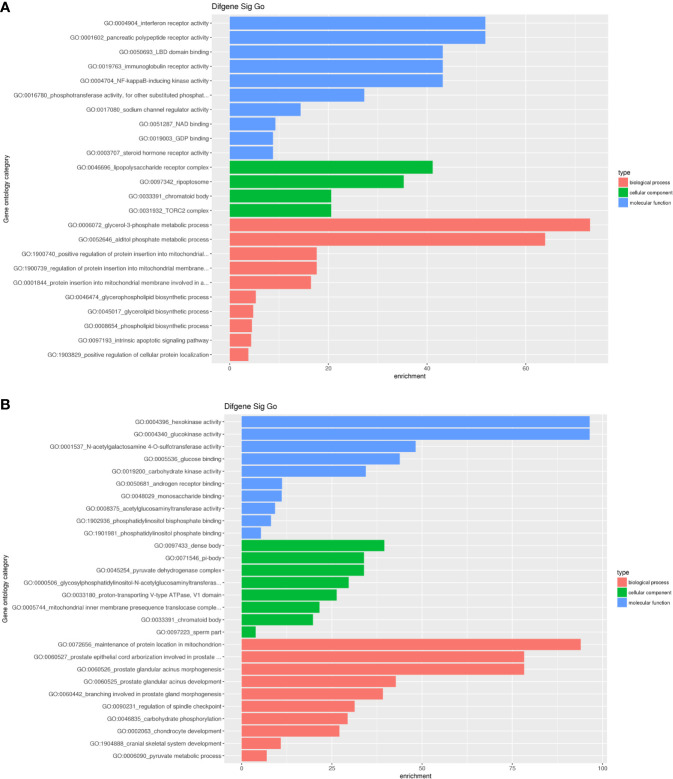
Significant GO enrichment terms correlated to differential mRNAs. **(A)** upregulated mRNAs were involved in interferon receptor activity, pancreatic polypeptide receptor activity, LBD domain binding(the first three biological processes), lipopolysaccharide receptor complex, ripoptosome, chromatoid body (the first three cellular components), glycerol-3-phosphate metabolic process, alditol phosphate metabolic process and positive regulation of protein insertion into mitochondrial(the first three molecular functions); **(B)** downregulated mRNAs were associated with hexokinase activity, glucokinase activity, N-acetylgalactosamine 4-O-sulfotransferase activity (the first three biological processes), dense body, pi-body, pyruvate dehydrogenase complex (the first three cellular components), maintenance of protein location in the mitochondrion, prostate epithelial cord arborization involved in prostate and prostate glandular acinus morphogenesis(the first three molecular functions).

To analyze the biological functions of differential mRNAs, we performed GO enrichment analysis. As the results presented, most upregulated mRNAs were involved in interferon receptor activity, pancreatic polypeptide receptor activity, LBD domain binding, lipopolysaccharide receptor complex, ripoptosome, chromatoid body, glycerol-3-phosphate metabolic process, alditol phosphate metabolic process and positive regulation of protein insertion into mitochondrial. Simultaneously, most downregulated mRNAs were associated with hexokinase activity, glucokinase activity, dense body, maintenance of protein location in the mitochondrion (**Figure 6**).

**Figure 7:**
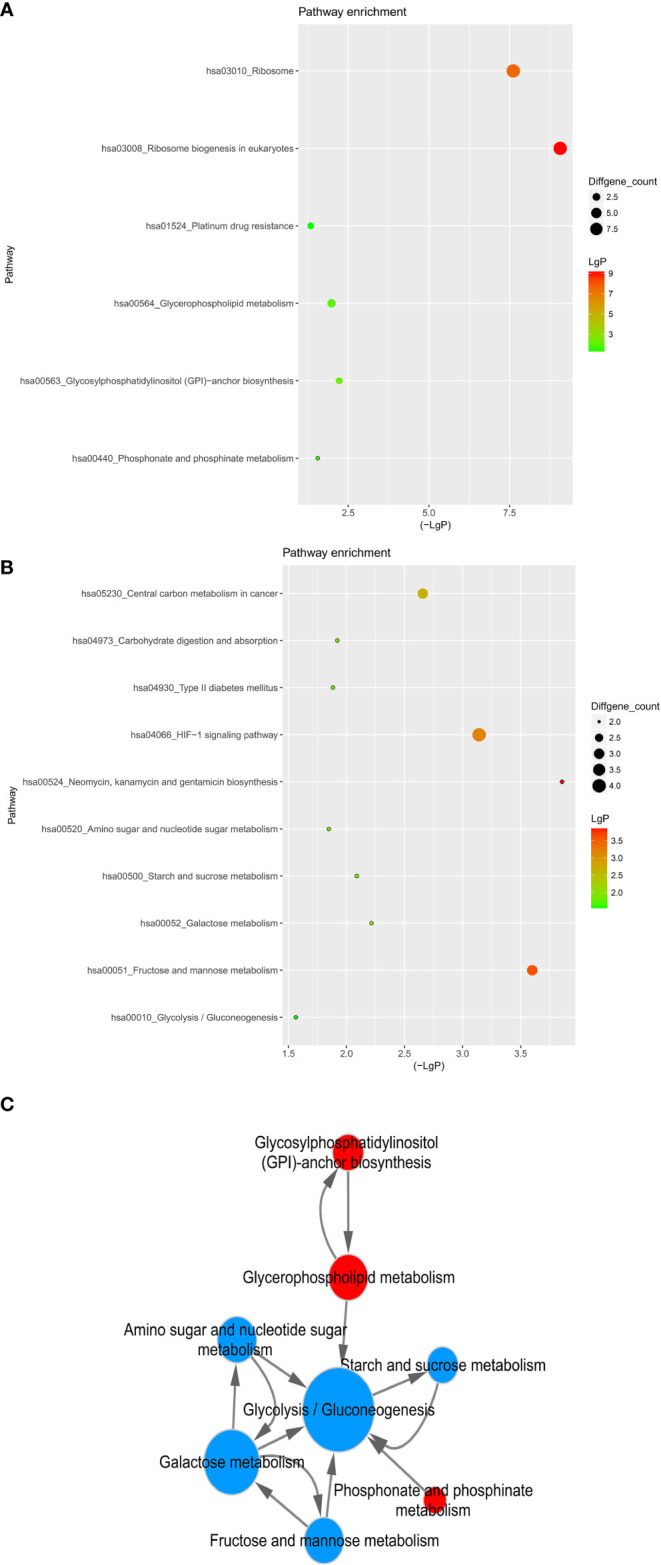
KEGG pathway of differential genes. **(A)** upregulated gene pathway enrichments; **(B)** downregulated gene pathway enrichments; **(C)** metabolism associated pathway network. The size of dots represent the gene count; LgP means the statistical significance, the red color means a greater significance than the green color and the horizontal axis shows the concrete value.

Then we carried out the KEGG pathway analysis for differential mRNAs. There was an amount of 84 pathways related to the upregulated mRNAs, and 68 signaling pathways were associated with downregulated mRNAs (**Figure 7**). Specifically, Ribosome biogenesis in eukaryotes and Ribosome (hsa03008 and hsa03010), Glycosylphosphatidylinositol (GPI)-anchor biosynthesis and Glycerophospholipid metabolism (hsa00563 and hsa00564), Phosphonate and phosphinate metabolism(hsa00440) and Platinum drug resistance(hsa01524) were obviously activated, whereas downregulated mRNAs were significantly relevant to Neomycin, kanamycin and gentamicin biosynthesis(hsa00524), Fructose and mannose metabolism(hsa00051), HIF-1 signaling pathway(hsa04066), Central carbon metabolism in cancer(hsa05230), Galactose metabolism(hsa00052), Starch and sucrose metabolism(hsa00500), Carbohydrate digestion and absorption(hsa04973), Type II diabetes mellitus(hsa04930), Amino sugar and nucleotide sugar metabolism(hsa00520), Glycolysis/Gluconeogenesis(hsa00010). The signaling pathway regulating glucose and lipid metabolism were both dysfunctional in breast cancer cells after the treatment of Hpa-V cells.

### Construction of Co-Expression Network

**Figure 8:**
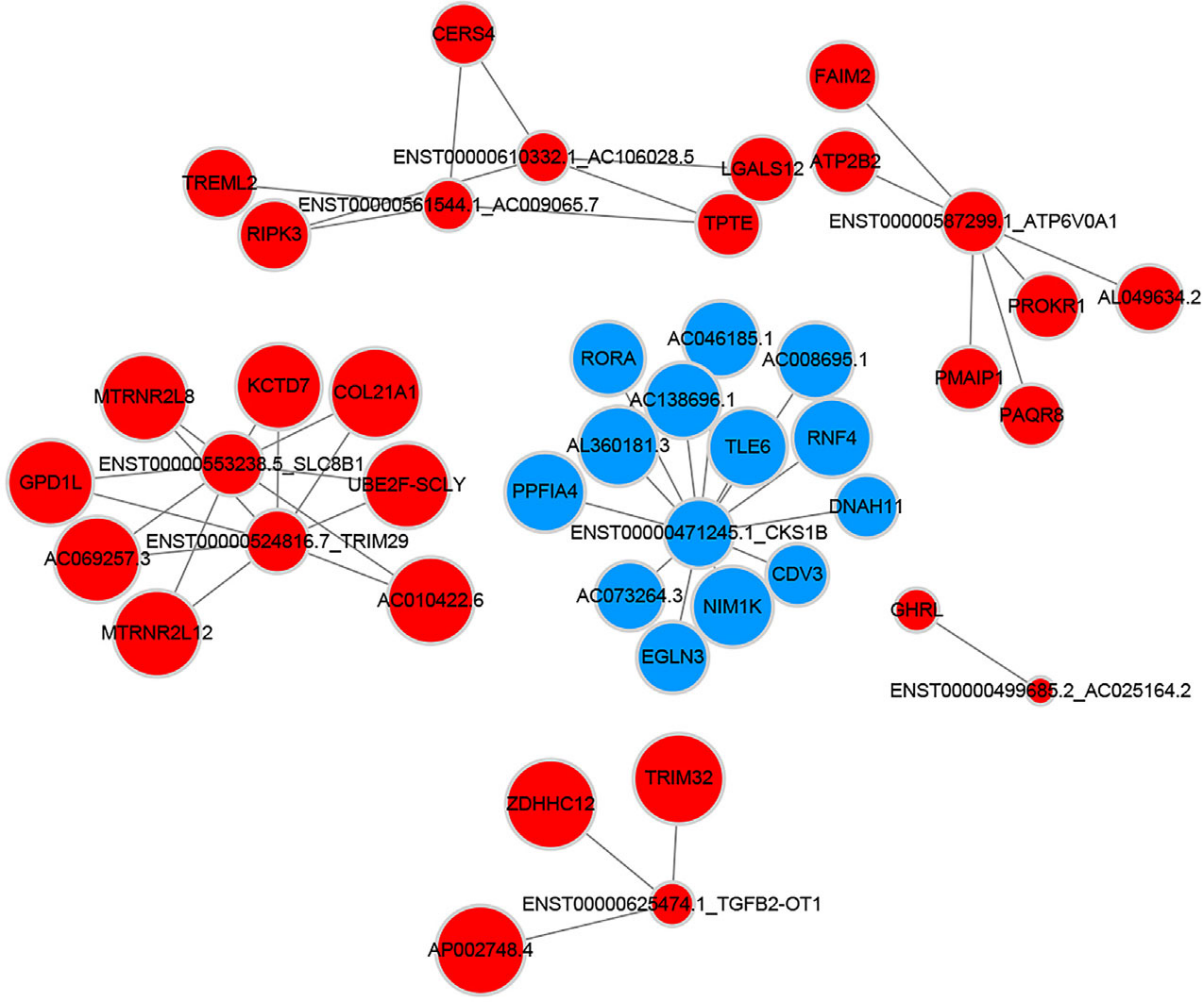
Metabolism related coding-non-coding gene co-expression networks (Blue nodes represent downregulated lncRNAs and mRNAs; red nodes represent upregulated lncRNAs and mRNAs).

To explore the molecular mechanism of adipocytes’ effects on breast cancer cells, we picked mRNAs related to lipid metabolism and depicted the mRNA-lncRNA gene network. As described in **Figure 8**, a total of 8 lncRNAs were connected with these metabolism-relative mRNAs. Within the network 34 connections were shown positive and 13 were negative.

## Discussion

Adipocytes were found to be the novel therapeutic target for breast cancer patients, due to their mutually active functions with cancer cells, resulting in tumor progression (14). In accordance with previous findings, our study confirmed that adipocytes tended to promote cancer invasion and migration of breast cancer cells. Meanwhile, the communication medium between adipocytes and breast cancer cells were under study. Researchers have already found that abnormal secretion of proinflammatory mediators or proangiogenic factors by adipocytes contributed to a more invasive tumor microenvironment, stimulating the development of breast cancer (15).

LncRNAs are transcripts with over 200 bases and function in transcription regulation of gene expression by influencing DNA methylation or activities of transcription factors, and post-transcription regulation by competing endogenous RNAs or stabilize proteins (16–19). In breast cancer studies, a large number of lncRNAs have been reported to be involved in cancer cell proliferation, apoptosis and metastasis (20–22). However, the role of lncRNAs in the relationship between adipocytes and breast cancer cells remains unclear. Therefore, in our study, we co-cultured breast cancer cells with adipocytes and analyzed the variation of lncRNA profiles in breast cancer cells before and after interactions with adipocytes. We found that 61 lncRNAs whose length were shorter than 2000bp were upregulated in MDA-MB-231/Hpa-V cells in contrast to MDA-MB-231 cells, while 69 lncRNAs were downregulated. Then we randomly selected 3 lncRNAs(ENST00000621974.1, ENST00000473798.1 and ENST00000608741.1) to examine the sequencing results by RT-qPCR and we knew that the results were in consistence. Furthermore, lncRNAs shorter than 2000bp were mainly processed transcript, antisense RNA and lincRNA.

We also analyzed the variation of mRNA profiles in breast cancer cells before and after co-cultured with adipocytes. Then we performed GO enrichment and KEGG pathway analysis to figure out the biological functions of differential mRNAs. The results came that after interacting with adipocytes, breast cancer cells were undergoing several transformations of material metabolism. For instance, the glycerophospholipid, glycosylphosphatidylinositol(GPI), lipopolysaccharide metabolism processes were strengthened to some extent. Additionally, previous literature has already reported that alterations in glucose metabolism of breast cancer cells impaired tumor initiation and metastasis (23). Besides, disordered lipid metabolism in breast cancer cells such as enhanced fatty acid β-oxidation was discovered to promote cancer progression and chemoresistance (24). Here, we could speculate that adipocytes might promote the progression of breast cancer cells by regulating cellular substance metabolism.

Moreover, we focused on the mRNAs correlated to glycolipid metabolism and constructed the mRNA-lncRNA network. In this way, we found the lncRNAs altered by adipocytes, which participated in the glucose and lipid metabolic process of breast cancer cells. Among these lncRNAs, ENST00000625474.1 was discovered to be significantly associated with gastric cancer metastasis and overall survival (25). Besides, transcripts ENST00000524816.7 and ENST00000471245.1 were respectively noncoding regions of gene TRIM29 and CKS1B, which were responsible for breast cancer proliferation, metastasis and invasion (26, 27). ENST00000587299.1 and ENST00000553238.5 participated in the transcription of gene SLC8B1 and ATP6V0A1 that was associated with the invasive process of oral tongue cancer and pancreatic ductal adenocarcinoma, respectively (28, 29). Therefore, lncRNAs may be the vital ingredients in the process of adipocytes induced progression of breast cancer cells.

Simultaneously, with the latest developments in preclinical and clinical medicine, organizations including National Comprehensive Cancer Network(NCCN) have proposed several recommendations for implementation of molecular analysis in the area of cancer risk stratification, therapeutic strategy and management (30). Therefore, with findings in our study, lncRNAs may be good biomarkers to better understand the logic of tumor microenvironment in obese breast cancer patients.

## Data Availability Statement

The datasets presented in this study can be found in online repositories. The names of the repository/repositories and accession number(s) can be found below: The SRA accessions for 6 samples are SRR12807060, SRR12807059, SRR12807058, SRR12807057, SRR12807056, SRR12807055. The BioProject accession: PRJNA668527.

## Author Contributions

J-HT and XC conceived and designed the experiments. X-HC, KY and M-XL performed the experiments and analyzed the data. X-HC, KY, M-XL, PM, DX, Y-JF and WZ prepared the figures and tables. J-HT, X-HC, KY, M-XL and XC drafted the work or revised it critically for important content. All authors contributed to the article and approved the submitted version.

## Funding

This research was supported by the National Natural Science Foundation of China (No. 81902987 and 81872365), National Key Research and Development Program of China (No. 2016YFC0905900) and Jiangsu Provincial Key Research Development Program (No. BE2019731).

## Conflict of Interest

The authors declare that the research was conducted in the absence of any commercial or financial relationships that could be construed as a potential conflict of interest.

## References

[1] Siegel RL Miller KD Jemal A. Cancer Statistics, 2020. CA Cancer J Clin (2020) 70(1):7–30. 10.3322/caac.21590 31912902

[2] Pfeiffer RM Webb-Vargas Y Wheeler W Gail MH. Proportion of U.S. Trends in Breast Cancer Incidence Attributable to Long-term Changes in Risk Factor Distributions. Cancer Epidemiol Biomarkers Prev (2018) 27(10):1214–22. 10.1158/1055-9965.EPI-18-0098 PMC8423092 30068516

[3] Pallegar NK Christian SL. Adipocytes in the Tumour Microenvironment. Adv Exp Med Biol (2020) 1234:1–13. 10.1007/978-3-030-37184-5_1 32040851

[4] Pahk K Joung C Kim S. Visceral Fat Metabolic Activity Evaluated by Preoperative (18)F-FDG PET/CT Significantly Affects Axillary Lymph Node Metastasis in Postmenopausal Luminal Breast Cancer. Sci Rep (2020) 10(1):1348. 10.1038/s41598-020-57937-4 31992764 PMC6987196

[5] Agurs-Collins T Ross SA Dunn BK. The Many Faces of Obesity and Its Influence on Breast Cancer Risk. Front Oncol (2019) 9:765. 10.3389/fonc.2019.00765 PMC6737012 31555578

[6] Ma L Bajic VB Zhang Z. On the Classification of Long Non-Coding RNAs. R Biol (2013) 10(6):925–33. 10.4161/rna.24604 PMC4111732 23696037

[7] Prabhu KS Raza A Karedath T Raza SS Fathima H Ahmed EI. Non-Coding RNAs as Regulators and Markers for Targeting of Breast Cancer and Cancer Stem Cells. Cancers (Basel) (2020) 12(2):351. 10.3390/cancers12020351 PMC7072613 32033146

[8] Dong H Hu J Zou K Ye M Chen Y Wu C. Activation of LncRNA TINCR by H3K27 Acetylation Promotes Trastuzumab Resistance and Epithelial-Mesenchymal Transition by Targeting MicroRNA-125b in Breast Cancer. Mol Cancer (2019) 18(1):3. 10.1186/s12943-018-0931-9 30621694 PMC6323810

[9] Dykes IM Emanueli C. Transcriptional and Post-Transcriptional Gene Regulation by Long Non-Coding RNA. Genomics Proteomics Bioinf (2017) 15(3):177–86. 10.1016/j.gpb.2016.12.005 PMC5487525 28529100

[10] Zhang J Liu L Li J Le TD. LncmiRSRN: Identification and Analysis of Long Non-Coding RNA Related miRNA Sponge Regulatory Network in Human Cancer. Bioinformatics (2018) 34(24):4232–40. 10.1093/bioinformatics/bty525 29955818

[11] Zhang FF Liu YH Wang DW Liu TS Yang Y Guo JM. Obesity-Induced Reduced Expression of the Lncrna ROIT Impairs Insulin Transcription by Downregulation of Nkx6.1 Methylation. Diabetologia (2020) 63(4):811–24. 10.1007/s00125-020-05090-y 32008054

[12] Yang L Wang X Guo H Zhang W Wang W Ma H. Whole Transcriptome Analysis of Obese Adipose Tissue Suggests u001kfc.1 as a Potential Regulator to Glucose Homeostasis. Front Genet (2019) 10:1133. 10.3389/fgene.2019.01133 31824561 PMC6881462

[13] Liu X Zhu Q Guo Y Xiao Z Hu L Xu Q. Lncrna LINC00689 Promotes the Growth, Metastasis and Glycolysis of Glioma Cells by Targeting miR-338-3p/PKM2 Axis. BioMed Pharmacother (2019) 117:109069. 10.1016/j.biopha.2019.109069 31181442

[14] Rybinska I Agresti R Trapani A Tagliabue E Triulzi T. Adipocytes in Breast Cancer, the Thick and the Thin. Cells (2020) 9(3):560. 10.3390/cells9030560 PMC7140407 32120856

[15] Zhang F Liu S. Mechanistic Insights of Adipocyte Metabolism in Regulating Breast Cancer Progression. P Res (2020) 155:104741. 10.1016/j.phrs.2020.104741 32151679

[16] Guttman M Russell P Ingolia NT Weissman JS Lander ES. Ribosome Profiling Provides Evidence That Large Noncoding RNAs do Not Encode Proteins. Cell (2013) 154(1):240–51. 10.1016/j.cell.2013.06.009 PMC3756563 23810193

[17] Yang L Lin C Jin C Yang JC Tanasa B Li W. lncRNA-dependent Mechanisms of Androgen-Receptor-Regulated Gene Activation Programs. Nature (2013) 500(7464):598–602. 10.1038/nature12451 23945587 PMC4034386

[18] Tsai MC Manor O Wan Y Mosammaparast N Wang JK Lan F. Long Noncoding RNA as Modular Scaffold of Histone Modification Complexes. Science (2010) 329(5992):689–93. 10.1126/science.1192002 PMC2967777 20616235

[19] Gong C Maquat LE. lncRNAs Transactivate STAU1-Mediated mRNA Decay by Duplexing With 3’ UTRs Via Alu Elements. Nature (2011) 470(7333):284–8. 10.1038/nature09701 PMC3073508 21307942

[20] Mendell JT. Targeting a Long Noncoding RNA in Breast Cancer. N Engl J Med (2016) 374(23):2287–9. 10.1056/NEJMcibr1603785 27276568

[21] Huarte M Guttman M Feldser D Garber M Koziol MJ Kenzelmann-Broz D. A Large Intergenic Noncoding RNA Induced by p53 Mediates Global Gene Repression in the p53 Response. Cell (2010) 142(3):409–19. 10.1016/j.cell.2010.06.040 PMC2956184 20673990

[22] Gupta RA Shah N Wang KC Kim J Horlings HM Wong DJ. Long Non-Coding RNA HOTAIR Reprograms Chromatin State to Promote Cancer Metastasis. Nature (2010) 464(7291):1071–6. 10.1038/nature08975 PMC3049919 20393566

[23] Semenza GL. Hypoxia-Inducible Factors: Coupling Glucose Metabolism and Redox Regulation With Induction Of the Breast Cancer Stem Cell Phenotype. EMBO J (2017) 36(3):252–9. 10.15252/embj.201695204 PMC5286373 28007895

[24] Wang T Fahrmann JF Lee H Li YJ Tripathi SC Yue C. Jak/Stat3-Regulated Fatty Acid β-Oxidation Is Critical for Breast Cancer Stem Cell Self-Renewal and Chemoresistance. Cell Metab (2018) 27(1):136–50.e5. 10.1016/j.cmet.2017.11.001 29249690 PMC5777338

[25] Song P Jiang B Liu Z Ding J Liu S Guan W. A Three-lncRNA Expression Signature Associated With the Prognosis of Gastric Cancer Patients. Cancer Med (2017) 6(6):1154–64. 10.1002/cam4.1047 PMC5463065 28444881

[26] Black JC Zhang H Kim J Getz G Whetstine JR. Regulation of Transient Site-Specific Copy Gain by Microrna. J Biol Chem (2016) 291(10):4862–71. 10.1074/jbc.M115.711648 PMC4777823 26755726

[27] Guo GC Wang JX Han ML Zhang LP Li L. microRNA-761 Induces Aggressive Phenotypes in Triple-Negative Breast Cancer Cells by Repressing TRIM29 Expression. Cell Oncol (Dordr) (2017) 40(2):157–66. 10.1007/s13402-016-0312-6 PMC13001536 28054302

[28] Pérez-Valencia JA Prosdocimi F Cesari IM da Costa IR Furtado C Agostini M. Angiogenesis and Evading Immune Destruction are the Main Related Transcriptomic Characteristics to the Invasive Process of Oral Tongue Cancer. Sci Rep (2018) 8(1):2007. 10.1038/s41598-017-19010-5 29386520 PMC5792437

[29] Flinck M Hagelund S Gorbatenko A Severin M Pedraz-Cuesta E Novak I. The Vacuolar H(+) ATPase α3 Subunit Negatively Regulates Migration and Invasion of Human Pancreatic Ductal Adenocarcinoma Cells. Cells (2020) 9(2):465. 10.3390/cells9020465 PMC7072798 32085585

[30] Goetz MP Gradishar WJ Anderson BO Abraham J Aft R Allison KH. NCCN Guidelines Insights: Breast Cancer, Version 3.2018. J Natl Compr Canc Netw (2019) 17: (2):118–26. 10.6004/jnccn.2019.0009 30787125

