# Supplementary material for: Variation of Long Non-Coding RNA And mRNA Profiles in Breast Cancer Cells With Influences of Adipocytes
Source: Front Oncol. 2021 May 21;11:631551. doi: 10.3389/fonc.2021.631551 (PMC8176020; doi:10.3389/fonc.2021.631551)
Supplement: Supplementary file 1 [file DataSheet_1.zip › sequencing/025G-201090513-CX-116_│┬╨π_6╚╦╤∙▒╛lncRNA_20190627/025G-201090513-CX-116_chenxiu_6╚╦╤∙▒╛lncRNA_20190627/1-Quality/clean/A1_clean_R1_fastqc/fastqc_report.html]

A1\_clean\_R1.fastq.gz FastQC Report 

FastQC Report

星期一 22 七月 2019  
A1\_clean\_R1.fastq.gz

## Summary

- Basic Statistics
- Per base sequence quality
- Per tile sequence quality
- Per sequence quality scores
- Per base sequence content
- Per sequence GC content
- Per base N content
- Sequence Length Distribution
- Sequence Duplication Levels
- Overrepresented sequences
- Adapter Content

## Basic Statistics

| Measure | Value |
| --- | --- |
| Filename | A1\_clean\_R1.fastq.gz |
| File type | Conventional base calls |
| Encoding | Sanger / Illumina 1.9 |
| Total Sequences | 53816416 |
| Sequences flagged as poor quality | 0 |
| Sequence length | 40-150 |
| %GC | 48 |

## Per base sequence quality

## Per tile sequence quality

## Per sequence quality scores

## Per base sequence content

## Per sequence GC content

## Per base N content

## Sequence Length Distribution

## Sequence Duplication Levels

## Overrepresented sequences

| Sequence | Count | Percentage | Possible Source |
| --- | --- | --- | --- |
| CCGAGAACGTATTCACCGTAGCGTAGCTGATCTACGATTACTAGCGATTC | 380092 | 0.7062752004890107 | No Hit |
| CGAGAACGTATTCACCGTAGCGTAGCTGATCTACGATTACTAGCGATTCC | 213213 | 0.39618580323148983 | No Hit |
| GTCTGATTAGTATTTAGCCTTACCGGGTGGTCCCGGCAGATTCAGACAGG | 198574 | 0.36898406612584533 | No Hit |
| CCCATTTTTAAGTGAAGCTGTGAAGCTCCTTTCTATTACTCATCATGCGA | 167248 | 0.3107750616466173 | No Hit |
| ATTCAGGCGGATCATTTAACGCGTTAGCTGCGTTAGTGAAATTATTCCAC | 165807 | 0.3080974400078965 | No Hit |
| GGCGGATCATTTAACGCGTTAGCTGCGTTAGTGAAATTATTCCACCAACT | 152395 | 0.28317567635867835 | No Hit |
| CCCCTCCTTAGGCAACCTGGTGGTCCCCCGCTCCCGGGAGGTCACCATAT | 125605 | 0.23339532680883096 | No Hit |
| CCAGGCTGGAGTGCAGTGGCTATTCACAGGCGCGATCCCACTACTGATCA | 123791 | 0.23002460810470915 | No Hit |
| GTCTGGAGTCTTGGAAGCTTGACTACCCTACGTTCTCCTACAAATGGACC | 123696 | 0.22984808204247567 | No Hit |
| CTGGAGTCTTGGAAGCTTGACTACCCTACGTTCTCCTACAAATGGACCTT | 121151 | 0.225119041743694 | No Hit |
| GTCCCTTAGTGTCAATATATAACCAGTTAGCTGCCTTCGCCTATTGGTGT | 120397 | 0.2237179822602828 | No Hit |
| CTGATTAGTATTTAGCCTTACCGGGTGGTCCCGGCAGATTCAGACAGGGT | 105496 | 0.1960294048566891 | No Hit |
| GTGGCTATTCACAGGCGCGATCCCACTACTGATCAGCACGGGAGTTTTGA | 105322 | 0.19570608343744034 | No Hit |
| GCTCAGGCTGGAGTGCAGTGGCTATTCACAGGCGCGATCCCACTACTGAT | 100360 | 0.18648584848162314 | No Hit |
| CCCTCCTTAGGCAACCTGGTGGTCCCCCGCTCCCGGGAGGTCACCATATT | 93460 | 0.1736644818562425 | No Hit |
| CCGGATAACGCTTGCGACCTATGTATTACCGCGGCTGCTGGCACATAGTT | 93095 | 0.17298625014345065 | No Hit |
| GCTCCGTTTCCGACCTGGGCCGGTTCACCCCTCCTTAGGCAACCTGGTGG | 92845 | 0.17252170787441512 | No Hit |
| CCTTAGTGTCAATATATAACCAGTTAGCTGCCTTCGCCTATTGGTGTTCT | 89865 | 0.1669843640275116 | No Hit |
| CACGTGTGTTGCCCCACTCGTAAGAGGCATGATGATTTGACGTCGTCCCC | 87162 | 0.16196173301469946 | No Hit |
| CCGGCATTCTCACTTTTAATCTCTCCACCAGTCCTCACGGTCTGACTTCA | 85404 | 0.15869507177884162 | No Hit |
| GGCTGGAGTGCAGTGGCTATTCACAGGCGCGATCCCACTACTGATCAGCA | 85274 | 0.15845350979894315 | No Hit |
| CCTCCTTAGGCAACCTGGTGGTCCCCCGCTCCCGGGAGGTCACCATATTG | 79573 | 0.14786008789585692 | No Hit |
| GCCCAGGCTGGAGTGCAGTGGCTATTCACAGGCGCGATCCCACTACTGAT | 78074 | 0.14507469245071986 | No Hit |
| CCACAATCCAGTAAGTGGTAGAACTATCCTTTTTCGTCACTCCATCATTC | 71260 | 0.13241312836588748 | No Hit |
| CCCCCATTAAACAATACTATACGCTAGCCCTAAAGCTATTTCGAAGAGAA | 67681 | 0.12576274124237483 | No Hit |
| CTCCGTTTCCGACCTGGGCCGGTTCACCCCTCCTTAGGCAACCTGGTGGT | 67676 | 0.12575345039699412 | No Hit |
| CAGGCTGGAGTGCAGTGGCTATTCACAGGCGCGATCCCACTACTGATCAG | 67371 | 0.12518670882877078 | No Hit |
| GGGGTCTTGTCGTCTTGATGCGGGTAACCAGCGTTTTCACTGGTACCATA | 65164 | 0.1210857296777251 | No Hit |
| GTCCCCACCTTCCTCCTGGTTACCCAGGCAGTATCTCTAGAGTCCTTAAC | 62655 | 0.11642358346568452 | No Hit |
| CTTAGTGTCAATATATAACCAGTTAGCTGCCTTCGCCTATTGGTGTTCTT | 61366 | 0.11402840352653734 | No Hit |
| GGGACCTTAGCTGACGATCTGGGTTGTTTCCCTCGCGAGCGTGGACGTTA | 58641 | 0.10896489279405006 | No Hit |
| CCATTTTTAAGTGAAGCTGTGAAGCTCCTTTCTATTACTCATCATGCGAT | 58630 | 0.1089444529342125 | No Hit |
| CTCCGTTTAACCTTCGGGCACTGGGCAGGCTTCACCCTCTATACGTCGTT | 58626 | 0.10893702025790793 | No Hit |
| CTCCATCATTCTTTTACCAAGTACAGGAATATTAACCTGTTGTCCATCGA | 58137 | 0.10802837557967443 | No Hit |
| AGCACGTGTGTTGCCCCACTCGTAAGAGGCATGATGATTTGACGTCGTCC | 55341 | 0.10283293484278105 | No Hit |

## Adapter Content

Produced by FastQC (version 0.11.7)
