# Supplementary material for: Variation of Long Non-Coding RNA And mRNA Profiles in Breast Cancer Cells With Influences of Adipocytes
Source: Front Oncol. 2021 May 21;11:631551. doi: 10.3389/fonc.2021.631551 (PMC8176020; doi:10.3389/fonc.2021.631551)
Supplement: Supplementary file 1 [file DataSheet_1.zip › sequencing/025G-201090513-CX-116_│┬╨π_6╚╦╤∙▒╛lncRNA_20190627/025G-201090513-CX-116_chenxiu_6╚╦╤∙▒╛lncRNA_20190627/1-Quality/clean/A1_clean_R2_fastqc/fastqc_report.html]

A1\_clean\_R2.fastq.gz FastQC Report 

FastQC Report

星期一 22 七月 2019  
A1\_clean\_R2.fastq.gz

## Summary

- Basic Statistics
- Per base sequence quality
- Per tile sequence quality
- Per sequence quality scores
- Per base sequence content
- Per sequence GC content
- Per base N content
- Sequence Length Distribution
- Sequence Duplication Levels
- Overrepresented sequences
- Adapter Content

## Basic Statistics

| Measure | Value |
| --- | --- |
| Filename | A1\_clean\_R2.fastq.gz |
| File type | Conventional base calls |
| Encoding | Sanger / Illumina 1.9 |
| Total Sequences | 53816416 |
| Sequences flagged as poor quality | 0 |
| Sequence length | 40-150 |
| %GC | 48 |

## Per base sequence quality

## Per tile sequence quality

## Per sequence quality scores

## Per base sequence content

## Per sequence GC content

## Per base N content

## Sequence Length Distribution

## Sequence Duplication Levels

## Overrepresented sequences

| Sequence | Count | Percentage | Possible Source |
| --- | --- | --- | --- |
| CTAAAATTAAAACAAAACCAATTTATTAAACACGCAATTTTTTGAGAGTT | 167756 | 0.3117190115372975 | No Hit |
| GCGGTGGCGCGTGCCTGTAGTCCCAGCTACTCGGGAGGCTGAGGCTGGAG | 111215 | 0.20665627380314586 | No Hit |
| GGTGGCGCGTGCCTGTAGTCCCAGCTACTCGGGAGGCTGAGGCTGGAGGA | 97007 | 0.18025540756931863 | No Hit |
| GCGGTGGCGCGTGCCTGTAGTCCCAGCTACTCGGGAGGCTGAGGTGGGAG | 90514 | 0.16819031575792784 | No Hit |
| GGTGGCGCGTGCCTGTAGTCCCAGCTACTCGGGAGGCTGAGGTGGGAGGA | 81700 | 0.15181241352081118 | No Hit |
| ATTAAAACAAAACCAATTTATTAAACACGCAATTTTTTGAGAGTTTGATC | 70905 | 0.131753478343857 | No Hit |
| GTTTTTTGCTAAGTCTGGAGTTAAATGCTGAAGCTCAACTTCAGTCCGCT | 55685 | 0.10347214500497394 | No Hit |

## Adapter Content

Produced by FastQC (version 0.11.7)
