# Supplementary material for: Variation of Long Non-Coding RNA And mRNA Profiles in Breast Cancer Cells With Influences of Adipocytes
Source: Front Oncol. 2021 May 21;11:631551. doi: 10.3389/fonc.2021.631551 (PMC8176020; doi:10.3389/fonc.2021.631551)
Supplement: Supplementary file 1 [file DataSheet_1.zip › sequencing/025G-201090513-CX-116_│┬╨π_6╚╦╤∙▒╛lncRNA_20190627/025G-201090513-CX-116_chenxiu_6╚╦╤∙▒╛lncRNA_20190627/1-Quality/clean/A2_clean_R1_fastqc/fastqc_report.html]

A2\_clean\_R1.fastq.gz FastQC Report 

FastQC Report

星期一 22 七月 2019  
A2\_clean\_R1.fastq.gz

## Summary

- Basic Statistics
- Per base sequence quality
- Per tile sequence quality
- Per sequence quality scores
- Per base sequence content
- Per sequence GC content
- Per base N content
- Sequence Length Distribution
- Sequence Duplication Levels
- Overrepresented sequences
- Adapter Content

## Basic Statistics

| Measure | Value |
| --- | --- |
| Filename | A2\_clean\_R1.fastq.gz |
| File type | Conventional base calls |
| Encoding | Sanger / Illumina 1.9 |
| Total Sequences | 55307646 |
| Sequences flagged as poor quality | 0 |
| Sequence length | 40-150 |
| %GC | 53 |

## Per base sequence quality

## Per tile sequence quality

## Per sequence quality scores

## Per base sequence content

## Per sequence GC content

## Per base N content

## Sequence Length Distribution

## Sequence Duplication Levels

## Overrepresented sequences

| Sequence | Count | Percentage | Possible Source |
| --- | --- | --- | --- |
| GCCCCATTGGCTCCTCAGCCAAGCACATACACCAAATGTCTGAACCTGCG | 296391 | 0.5358951635728629 | No Hit |
| CCGAGAACGTATTCACCGTAGCGTAGCTGATCTACGATTACTAGCGATTC | 228364 | 0.41289770314939817 | No Hit |
| GGCAGACGTTCGAATGGGTCGTCGCCGCCACGGGGGGCGTGCGATCGGCC | 176467 | 0.31906438397323944 | No Hit |
| GTCGGCATGTATTAGCTCTAGAATTACCACAGTTATCCAAGTAGGAGAGG | 157755 | 0.2852318104444365 | No Hit |
| CGAGAACGTATTCACCGTAGCGTAGCTGATCTACGATTACTAGCGATTCC | 137299 | 0.2482459658471091 | No Hit |
| CCCATTTTTAAGTGAAGCTGTGAAGCTCCTTTCTATTACTCATCATGCGA | 127535 | 0.23059198722722712 | No Hit |
| GTCTGATTAGTATTTAGCCTTACCGGGTGGTCCCGGCAGATTCAGACAGG | 123907 | 0.22403231553192482 | No Hit |
| GCTGGATAGTAGGTAGGGACAGTGGGAATCTCGTTCATCCATTCATGCGC | 120090 | 0.2171309189329808 | No Hit |
| GCCCGAGGTTATCTAGAGTCACCAAAGCCGCCGGCGCCCGCCCCCCGGCC | 119979 | 0.21693022335465156 | No Hit |
| GCTGTGGTTTCGCTGGATAGTAGGTAGGGACAGTGGGAATCTCGTTCATC | 117105 | 0.2117338351373696 | No Hit |
| CCCCGCCTCACCGGGTCAGTGAAAAAACGATCAGAGTAGTGGTATTTCAC | 113102 | 0.2044961378396036 | No Hit |
| GGCGGATCATTTAACGCGTTAGCTGCGTTAGTGAAATTATTCCACCAACT | 111481 | 0.20156525916868712 | No Hit |
| ATTCAGGCGGATCATTTAACGCGTTAGCTGCGTTAGTGAAATTATTCCAC | 105286 | 0.19036427621598648 | No Hit |
| GTCTGGAGTCTTGGAAGCTTGACTACCCTACGTTCTCCTACAAATGGACC | 102026 | 0.18446997364523524 | No Hit |
| CTGGAGTCTTGGAAGCTTGACTACCCTACGTTCTCCTACAAATGGACCTT | 99732 | 0.18032226502643053 | No Hit |
| GTCCCTTAGTGTCAATATATAACCAGTTAGCTGCCTTCGCCTATTGGTGT | 97928 | 0.17706050986151173 | No Hit |
| CCCGTCGGCATGTATTAGCTCTAGAATTACCACAGTTATCCAAGTAGGAG | 97239 | 0.17581475082125173 | No Hit |
| GTTCCCTTGGCTGTGGTTTCGCTGGATAGTAGGTAGGGACAGTGGGAATC | 92903 | 0.1679749667884979 | No Hit |
| CCCGAGGTTATCTAGAGTCACCAAAGCCGCCGGCGCCCGCCCCCCGGCCG | 91908 | 0.16617593885662754 | No Hit |
| GTCCTGTATTGTTATTTTTCGTCACTACCTCCCCGGGTCGGGAGTGGGTA | 87878 | 0.15888942371548412 | No Hit |
| CCCCTCCTTAGGCAACCTGGTGGTCCCCCGCTCCCGGGAGGTCACCATAT | 87778 | 0.15870861688816046 | No Hit |
| CCAGGCTGGAGTGCAGTGGCTATTCACAGGCGCGATCCCACTACTGATCA | 86000 | 0.15549387149834581 | No Hit |
| GTCCTAACACGTGCGCTCGTGCTCCACCTCCCCGGCGCGGCGGGCGAGAC | 83450 | 0.15088329740159254 | No Hit |
| GTGGCTATTCACAGGCGCGATCCCACTACTGATCAGCACGGGAGTTTTGA | 79114 | 0.14304351336883872 | No Hit |
| GCGGTATCCAGGCGGCTCGGGCCTGCTTTGAACACTCTAATTTTTTCAAA | 78129 | 0.1412625661197007 | No Hit |
| GCTCAGGCTGGAGTGCAGTGGCTATTCACAGGCGCGATCCCACTACTGAT | 76886 | 0.13901513725606765 | No Hit |
| CTCTCATGTCTCTTCACCGTGCCAGACTAGAGTCAAGCTCAACAGGGTCT | 75775 | 0.13700637340450178 | No Hit |
| CCTTAGTGTCAATATATAACCAGTTAGCTGCCTTCGCCTATTGGTGTTCT | 73827 | 0.13348425640823694 | No Hit |
| GTTGGTTTTGATCTGATAAATGCACGCATCCCCCCCGCGAAGGGGGTCAG | 71651 | 0.12954989984567414 | No Hit |
| CCGGATAACGCTTGCGACCTATGTATTACCGCGGCTGCTGGCACATAGTT | 70842 | 0.12808717261262575 | No Hit |
| CTCCAATGGATCCTCGTTAAAGGATTTAAAGTGGACTCATTCCAATTACA | 70786 | 0.1279859207893245 | No Hit |
| GCCGTATCGTTCCGCCTGGGCGGGATTCTGACTTAGAGGCGTTCAGTCAT | 68640 | 0.1241058062749588 | No Hit |
| CTGATTAGTATTTAGCCTTACCGGGTGGTCCCGGCAGATTCAGACAGGGT | 68529 | 0.12390511069662953 | No Hit |
| GCTCCGTTTCCGACCTGGGCCGGTTCACCCCTCCTTAGGCAACCTGGTGG | 66854 | 0.12087659633895827 | No Hit |
| CCGTCGGCATGTATTAGCTCTAGAATTACCACAGTTATCCAAGTAGGAGA | 66269 | 0.11981887639911487 | No Hit |
| CCCTCCTTAGGCAACCTGGTGGTCCCCCGCTCCCGGGAGGTCACCATATT | 66198 | 0.11969050355171507 | No Hit |
| CCCGAAGTTACGGATCCGGCTTGCCGACTTCCCTTACCTACATTGTTCCA | 66191 | 0.11967784707380243 | No Hit |
| CACGTGTGTTGCCCCACTCGTAAGAGGCATGATGATTTGACGTCGTCCCC | 66138 | 0.11958201945532088 | No Hit |
| CCGGATAAAACTGCGTGGCGGGGGTGCGTCGGGTCTGCGAGAGCGCCAGC | 64895 | 0.11733459059168781 | No Hit |
| CCCCAGTCATCAGTCCTGCCTTAGGCAATGGTCTCCGAAGTTAACTCACC | 63275 | 0.11440551998904455 | No Hit |
| GGCTGGAGTGCAGTGGCTATTCACAGGCGCGATCCCACTACTGATCAGCA | 62335 | 0.11270593581220217 | No Hit |
| CTCCGACTTTCGTTCTTGATTAATGAAAACATTCTTGGCAAATGCTTTCG | 61103 | 0.1104783956995747 | No Hit |
| GTGGTTTCGCTGGATAGTAGGTAGGGACAGTGGGAATCTCGTTCATCCAT | 61018 | 0.1103247098963496 | No Hit |
| CCTGTATTGTTATTTTTCGTCACTACCTCCCCGGGTCGGGAGTGGGTAAT | 57030 | 0.10311413362268211 | No Hit |
| CTTGGCTGTGGTTTCGCTGGATAGTAGGTAGGGACAGTGGGAATCTCGTT | 56648 | 0.10242345154230575 | No Hit |
| CACCTCTCATGTCTCTTCACCGTGCCAGACTAGAGTCAAGCTCAACAGGG | 56474 | 0.10210884766276257 | No Hit |
| CCGGCATTCTCACTTTTAATCTCTCCACCAGTCCTCACGGTCTGACTTCA | 55702 | 0.10071301895582394 | No Hit |
| ATCTGATAAATGCACGCATCCCCCCCGCGAAGGGGGTCAGCGCCCGTCGG | 55548 | 0.10043457644174551 | No Hit |

## Adapter Content

Produced by FastQC (version 0.11.7)
