# Supplementary material for: Variation of Long Non-Coding RNA And mRNA Profiles in Breast Cancer Cells With Influences of Adipocytes
Source: Front Oncol. 2021 May 21;11:631551. doi: 10.3389/fonc.2021.631551 (PMC8176020; doi:10.3389/fonc.2021.631551)
Supplement: Supplementary file 1 [file DataSheet_1.zip › sequencing/025G-201090513-CX-116_│┬╨π_6╚╦╤∙▒╛lncRNA_20190627/025G-201090513-CX-116_chenxiu_6╚╦╤∙▒╛lncRNA_20190627/1-Quality/clean/A2_clean_R2_fastqc/fastqc_report.html]

A2\_clean\_R2.fastq.gz FastQC Report 

FastQC Report

星期一 22 七月 2019  
A2\_clean\_R2.fastq.gz

## Summary

- Basic Statistics
- Per base sequence quality
- Per tile sequence quality
- Per sequence quality scores
- Per base sequence content
- Per sequence GC content
- Per base N content
- Sequence Length Distribution
- Sequence Duplication Levels
- Overrepresented sequences
- Adapter Content

## Basic Statistics

| Measure | Value |
| --- | --- |
| Filename | A2\_clean\_R2.fastq.gz |
| File type | Conventional base calls |
| Encoding | Sanger / Illumina 1.9 |
| Total Sequences | 55307646 |
| Sequences flagged as poor quality | 0 |
| Sequence length | 40-150 |
| %GC | 53 |

## Per base sequence quality

## Per tile sequence quality

## Per sequence quality scores

## Per base sequence content

## Per sequence GC content

## Per base N content

## Sequence Length Distribution

## Sequence Duplication Levels

## Overrepresented sequences

| Sequence | Count | Percentage | Possible Source |
| --- | --- | --- | --- |
| CCTGCCAGTAGCATATGCTTGTCTCAAAGATTAAGCCATGCATGTCTAAG | 198507 | 0.35891420871537366 | No Hit |
| GTCAAAGTGAAGAAATTCAATGAAGCGCGGGTAAACGGCGGGAGTAACTA | 144192 | 0.26070898045452884 | No Hit |
| CTAAAATTAAAACAAAACCAATTTATTAAACACGCAATTTTTTGAGAGTT | 131399 | 0.23757836303501326 | No Hit |
| GGCGGGAGTAACTATGACTCTCTTAAGGTAGCCAAATGCCTCGTCATCTA | 102659 | 0.18561448086219398 | No Hit |
| GTGAAACTGCGAATGGCTCATTAAATCAGTTATGGTTCCTTTGGTCGCTC | 94820 | 0.17144103366829244 | No Hit |
| GTTGTTGCCATGGTAATCCTGCTCAGTACGAGAGGAACCGCAGGTTCAGA | 85721 | 0.1549894204501128 | No Hit |
| GTTAGTTTTACCCTACTGATGATGTGTTGTTGCCATGGTAATCCTGCTCA | 78190 | 0.14137285828436813 | No Hit |
| GGTAATTCTAGAGCTAATACATGCCGACGGGCGCTGACCCCCTTCGCGGG | 72415 | 0.1309312640064269 | No Hit |
| GATTAAGCCATGCATGTCTAAGTACGCACGGCCGGTACAGTGAAACTGCG | 70822 | 0.12805101124716103 | No Hit |
| GCGGTGGCGCGTGCCTGTAGTCCCAGCTACTCGGGAGGCTGAGGCTGGAG | 70526 | 0.127515823038283 | No Hit |
| GTAACTATGACTCTCTTAAGGTAGCCAAATGCCTCGTCATCTAATTAGTG | 69669 | 0.12596630852811924 | No Hit |
| CAAAGATTAAGCCATGCATGTCTAAGTACGCACGGCCGGTACAGTGAAAC | 69302 | 0.12530274747184142 | No Hit |
| GGGGAATCAGGGTTCGATTCCGGAGAGGGAGCCTGAGAAACGGCTACCAC | 68271 | 0.12343862908213449 | No Hit |
| CTGAATGTCAAAGTGAAGAAATTCAATGAAGCGCGGGTAAACGGCGGGAG | 66456 | 0.12015698516621011 | No Hit |
| GGTGGCGCGTGCCTGTAGTCCCAGCTACTCGGGAGGCTGAGGCTGGAGGA | 65979 | 0.11929453659987627 | No Hit |
| GAGACAGGTTAGTTTTACCCTACTGATGATGTGTTGTTGCCATGGTAATC | 63098 | 0.11408549190468167 | No Hit |
| GCGGTGGCGCGTGCCTGTAGTCCCAGCTACTCGGGAGGCTGAGGTGGGAG | 60840 | 0.11000287374371348 | No Hit |
| GGTGGCGCGTGCCTGTAGTCCCAGCTACTCGGGAGGCTGAGGTGGGAGGA | 59427 | 0.1074480732736302 | No Hit |
| GTCTAAGTACGCACGGCCGGTACAGTGAAACTGCGAATGGCTCATTAAAT | 57325 | 0.10364751376328692 | No Hit |
| GACTAATCGAACCATCTAGTAGCTGGTTCCCTCCGAAGTTTCCCTCAGGA | 56463 | 0.10208895891175697 | No Hit |
| ATTAAAACAAAACCAATTTATTAAACACGCAATTTTTTGAGAGTTTGATC | 55707 | 0.10072205929719012 | No Hit |

## Adapter Content

Produced by FastQC (version 0.11.7)
