# Supplementary material for: Variation of Long Non-Coding RNA And mRNA Profiles in Breast Cancer Cells With Influences of Adipocytes
Source: Front Oncol. 2021 May 21;11:631551. doi: 10.3389/fonc.2021.631551 (PMC8176020; doi:10.3389/fonc.2021.631551)
Supplement: Supplementary file 1 [file DataSheet_1.zip › sequencing/025G-201090513-CX-116_│┬╨π_6╚╦╤∙▒╛lncRNA_20190627/025G-201090513-CX-116_chenxiu_6╚╦╤∙▒╛lncRNA_20190627/1-Quality/clean/A3_clean_R1_fastqc/fastqc_report.html]

A3\_clean\_R1.fastq.gz FastQC Report 

FastQC Report

星期二 23 七月 2019  
A3\_clean\_R1.fastq.gz

## Summary

- Basic Statistics
- Per base sequence quality
- Per tile sequence quality
- Per sequence quality scores
- Per base sequence content
- Per sequence GC content
- Per base N content
- Sequence Length Distribution
- Sequence Duplication Levels
- Overrepresented sequences
- Adapter Content

## Basic Statistics

| Measure | Value |
| --- | --- |
| Filename | A3\_clean\_R1.fastq.gz |
| File type | Conventional base calls |
| Encoding | Sanger / Illumina 1.9 |
| Total Sequences | 54992436 |
| Sequences flagged as poor quality | 0 |
| Sequence length | 40-150 |
| %GC | 48 |

## Per base sequence quality

## Per tile sequence quality

## Per sequence quality scores

## Per base sequence content

## Per sequence GC content

## Per base N content

## Sequence Length Distribution

## Sequence Duplication Levels

## Overrepresented sequences

| Sequence | Count | Percentage | Possible Source |
| --- | --- | --- | --- |
| CCGAGAACGTATTCACCGTAGCGTAGCTGATCTACGATTACTAGCGATTC | 812550 | 1.477566842101703 | No Hit |
| CTCAGGTGGATCATTTAATGCGTTAGCTGCGCTAGTGAAAAATTCCACCA | 591463 | 1.075535188148421 | No Hit |
| CGAGAACGTATTCACCGTAGCGTAGCTGATCTACGATTACTAGCGATTCC | 444862 | 0.808951252859575 | No Hit |
| GTCTGGTTAGTATTTAGCCTTACCGGATGGTCCCGGCAGATTCAGACAGG | 198927 | 0.36173520300137274 | No Hit |
| CCTCAGTGTCAGTACATAACCAGTTAGCTGCCTTCGCCATTGATGTTCTT | 179077 | 0.32563932974345783 | No Hit |
| CTGGTTAGTATTTAGCCTTACCGGATGGTCCCGGCAGATTCAGACAGGGT | 176392 | 0.32075684008615296 | No Hit |
| GGATCATTTAATGCGTTAGCTGCGCTAGTGAAAAATTCCACCAGCTAATG | 172223 | 0.3131757974860397 | No Hit |
| CCTCTTGCTACTAAGATGTTTCAATTCACAAGGTGTCTCGCTCACACTTT | 146492 | 0.26638572621151024 | No Hit |
| CCGGCATTCTCACTTCTAAGCACTCCACTAATCCTCACGGTTTAGCTTCG | 134880 | 0.24527009496360555 | No Hit |
| CTCAGTGTCAGTACATAACCAGTTAGCTGCCTTCGCCATTGATGTTCTTC | 122085 | 0.22200325877544322 | No Hit |
| GGGACCTTAGCTGACGATCTGGGTTGTTACCCTCGCGAGCATCGACGTTA | 121523 | 0.2209813000464282 | No Hit |
| GTCAGTACATAACCAGTTAGCTGCCTTCGCCATTGATGTTCTTCCTAATA | 120374 | 0.2188919217908441 | No Hit |
| CACCGCTTCACTAGGAATTCCACTAACCTCTTTATAACTCTAGTTTGCTA | 114907 | 0.20895055458172465 | No Hit |
| CCAAAATCCATTAATTGGTAGAATTATCCTTCTCCGTCACTCCATCACTT | 110565 | 0.20105492326253743 | No Hit |
| GGGCTCTTCTGCTTTCGCTCGCCACTACTAACAGAATCATTATTTATTTT | 106710 | 0.19404486827970305 | No Hit |
| CCTCAACTTAATGTAGTAATTAAAGATAGGGGTTGCGCTCGTTGCAGGAC | 103438 | 0.1880949590958291 | No Hit |
| CCCCTATACATCGTCTTGCGACTTAGCAGAGAGCTGTGTTTTTGATAAAC | 103087 | 0.18745668949817024 | No Hit |
| CCCCACCTTCCTACCAATTGCTTGGTCAGTATCTTTAAAGTCCTCAACTT | 100024 | 0.18188683258184818 | No Hit |
| CCTCTTTATAACTCTAGTTTGCTAGTATCAAAAGCGGGCCAGGGTTGAGC | 99963 | 0.18177590823581627 | No Hit |
| CTTCGCCATTGATGTTCTTCCTAATATCTACACATTTCACCGCTTCACTA | 99062 | 0.18013750109196836 | No Hit |
| GGTGGATCATTTAATGCGTTAGCTGCGCTAGTGAAAAATTCCACCAGCTA | 98578 | 0.17925738005132197 | No Hit |
| GTCCGTTCTCGGTACGGGTTCCTATAGTTTTATAACGTTTAGAAGCTTTT | 98572 | 0.179246469459909 | No Hit |
| GCTCCACGGGGTCTTTTCGTTTTGATGCGGGTATCCAGCGTTTTCACTGG | 96405 | 0.17530592752792404 | No Hit |
| GTGGTATTTCAAGGTTGATTCCACAGAAACTAGCGTCTCCGCTTCTCCAT | 96374 | 0.17524955613895699 | No Hit |
| CCCATTTCAAAGTGAAGCAAACGCTCCTTTGATATCTAAATCATGCGATC | 96123 | 0.17479312973151434 | No Hit |
| CACGTGTGTTGCCCCACTCGTAAGAGGCATGATGATTTGACGTCATCCCC | 95601 | 0.1738439082785858 | No Hit |
| GTCTCGCTCACACTTTCTATATATTCAAAAGTGGGCAACTAGAGATTAAT | 92279 | 0.16780307749960377 | No Hit |
| GGGGTCTTTTCGTTTTGATGCGGGTATCCAGCGTTTTCACTGGAACCATA | 92182 | 0.16762668960509405 | No Hit |
| GCCCCTCACAATTTAATGTTGCCATTCAAAGAATGGCCCCCCTTCTCGCG | 91769 | 0.16687567722950117 | No Hit |
| CCCGGGGTAGCTTTTATCCGTTGAGCGACAGCCATTCCATAATGAACTGC | 90234 | 0.16408438425968255 | No Hit |
| GTCTGGAGTCTTGGAAGCTTGACTACCCTACGTTCTCCTACAAATGGACC | 88459 | 0.16085666763334508 | No Hit |
| GCCCTATTCAGACTCGATTTCTCTTCGGCTCCGCTTTTATCCACTTAACC | 87068 | 0.15832722885743777 | No Hit |
| CTACGAACTCTTTACACCCAATAATTTCGGATAACGCTTGCACCCTATGT | 86230 | 0.15680338292342605 | No Hit |
| CTGGAGTCTTGGAAGCTTGACTACCCTACGTTCTCCTACAAATGGACCTT | 85914 | 0.15622875844234288 | No Hit |
| GCTCCATGTCACCATCTTGCTTCTCTTTGTACCGGCCATTGTAGCACGTG | 80434 | 0.1462637516184953 | No Hit |
| GTTAGTATTTAGCCTTACCGGATGGTCCCGGCAGATTCAGACAGGGTTTC | 80124 | 0.14570003772882512 | No Hit |
| GGCTGTTCAGTCTCTCAACTCAGCTATACATCATCGCCTTGGTAGGCCAT | 78929 | 0.14352701160574155 | No Hit |
| CAATAATTTCGGATAACGCTTGCACCCTATGTATTACCGCAGCTGCTGGC | 77476 | 0.14088483005190022 | No Hit |
| CTCCCATTTCAAAGTGAAGCAAACGCTCCTTTGATATCTAAATCATGCGA | 77211 | 0.14040294559782732 | No Hit |
| GACCCGAGAACGTATTCACCGTAGCGTAGCTGATCTACGATTACTAGCGA | 76664 | 0.1394082633473447 | No Hit |
| CCAGGCTGGAGTGCAGTGGCTATTCACAGGCGCGATCCCACTACTGATCA | 76506 | 0.13912095110680311 | No Hit |
| CTCGCTCACACTTTCTATATATTCAAAAGTGGGCAACTAGAGATTAATCT | 76263 | 0.13867907215457778 | No Hit |
| GATCATTTAATGCGTTAGCTGCGCTAGTGAAAAATTCCACCAGCTAATGA | 76217 | 0.13859542428707833 | No Hit |
| CCCCGGGGTAGCTTTTATCCGTTGAGCGACAGCCATTCCATAATGAACTG | 75849 | 0.1379262413470827 | No Hit |
| GGTATTTCAAGGTTGATTCCACAGAAACTAGCGTCTCCGCTTCTCCATCT | 75487 | 0.13726796899850008 | No Hit |
| GCTCAGGCTGGAGTGCAGTGGCTATTCACAGGCGCGATCCCACTACTGAT | 74364 | 0.13522586997237218 | No Hit |
| GCTCGCCTTAGAATACTCATCTTGGGAACGTGTGTCCGTTCTCGGTACGG | 72124 | 0.1311525825115294 | No Hit |
| CCCTACTGCTGCCTCCCGTAGGAGTCTGGGCCGTATCTCAGTCCCAGTGT | 70703 | 0.12856859077855726 | No Hit |
| CCTCATTTCCTTGCCCCAAAATCCATTAATTGGTAGAATTATCCTTCTCC | 68965 | 0.12540815613259976 | No Hit |
| CTCGTAGTCTACAAGGAATTTCAAGGGAATACTCATCTTTGAGGAGGCTT | 68870 | 0.12523540510189438 | No Hit |
| CCCACATCAAATAGGGACCGAACTGTCTCACGACGTTCTGAACCCAGCTC | 66750 | 0.12138032946931103 | No Hit |
| CCCCTCCTTAGGCAACCTGGTGGTCCCCCGCTCCCGGGAGGTCACCATAT | 66400 | 0.12074387830355432 | No Hit |
| GCGTTAGCTGCGCTAGTGAAAAATTCCACCAGCTAATGATCATCGTTTAC | 65966 | 0.11995467885801603 | No Hit |
| CTTATAACAGCAGTTTACAATCCGAAGACCTTCATCCTGCACGCTGTGTC | 65779 | 0.11961463209231174 | No Hit |
| CCCGAGAACGTATTCACCGTAGCGTAGCTGATCTACGATTACTAGCGATT | 65424 | 0.11896908876704426 | No Hit |
| CTCCGTCACTCCATCACTTACTATCGGAAGTACAGGAATATTAACCTGTT | 65206 | 0.11857267061237294 | No Hit |
| CACGGGGTCTTTTCGTTTTGATGCGGGTATCCAGCGTTTTCACTGGAACC | 64942 | 0.11809260459020218 | No Hit |
| GTTCAGTCTCTCAACTCAGCTATACATCATCGCCTTGGTAGGCCATTACC | 64922 | 0.11805623595215894 | No Hit |
| AGCACGTGTGTTGCCCCACTCGTAAGAGGCATGATGATTTGACGTCATCC | 64484 | 0.11725976277901201 | No Hit |
| CTCCCACCTATCCTACACATGTTAAACCAAATTCCAATACGAAGTTGTAG | 62932 | 0.11443755646685665 | No Hit |
| GTCACTCCATCAGACTTTCGTCCATTGTGGAAAATTCCCTACTGCTGCCT | 62699 | 0.1140138618336529 | No Hit |
| GTTACATTGTCGGCGCAAGGTCTCTTGACTAGTGAGCTATTACGCACTCT | 62683 | 0.11398476692321832 | No Hit |
| GTACAAGACCCGAGAACGTATTCACCGTAGCGTAGCTGATCTACGATTAC | 62611 | 0.11385383982626265 | No Hit |
| GTTCCGACTGCATGATAATACACAATGGTATTCGGAGTTTGATTATAGTC | 62507 | 0.11366472290843782 | No Hit |
| CCCTCATTTCCTTGCCCCAAAATCCATTAATTGGTAGAATTATCCTTCTC | 62507 | 0.11366472290843782 | No Hit |
| CGGGCACTGGGCAGACTTCACCCCCTATACATCGTCTTGCGACTTAGCAG | 62105 | 0.11293371328376871 | No Hit |
| CCACGGGGTCTTTTCGTTTTGATGCGGGTATCCAGCGTTTTCACTGGAAC | 62033 | 0.11280278618681304 | No Hit |
| GTGGCTATTCACAGGCGCGATCCCACTACTGATCAGCACGGGAGTTTTGA | 61923 | 0.11260275867757523 | No Hit |
| GTGTCTGGTTAGTATTTAGCCTTACCGGATGGTCCCGGCAGATTCAGACA | 61837 | 0.1124463735339893 | No Hit |
| TCCCATTTCAAAGTGAAGCAAACGCTCCTTTGATATCTAAATCATGCGAT | 61451 | 0.11174445881975477 | No Hit |
| GGCTGGAGTGCAGTGGCTATTCACAGGCGCGATCCCACTACTGATCAGCA | 60967 | 0.11086433777910838 | No Hit |
| TCTGGTTAGTATTTAGCCTTACCGGATGGTCCCGGCAGATTCAGACAGGG | 60963 | 0.11085706405149973 | No Hit |
| GCACGTGTGTTGCCCCACTCGTAAGAGGCATGATGATTTGACGTCATCCC | 59589 | 0.10835853861792921 | No Hit |
| CAGGTGGATCATTTAATGCGTTAGCTGCGCTAGTGAAAAATTCCACCAGC | 59031 | 0.10734385361652282 | No Hit |
| AGCGATTCCAACTTCATGAAGTCGAGTTGCAGACTTCAATCCGAACTGAG | 58516 | 0.10640736118690941 | No Hit |
| CGGCCATTGTAGCACGTGTGTTGCCCCACTCGTAAGAGGCATGATGATTT | 58481 | 0.10634371607033376 | No Hit |
| CCTACTGCTGCCTCCCGTAGGAGTCTGGGCCGTATCTCAGTCCCAGTGTG | 58288 | 0.10599275871321649 | No Hit |
| CCCTTATAACAGCAGTTTACAATCCGAAGACCTTCATCCTGCACGCTGTG | 58159 | 0.1057581809978376 | No Hit |
| GTTCCTCAGTGTCAGTACATAACCAGTTAGCTGCCTTCGCCATTGATGTT | 57240 | 0.10408704207975075 | No Hit |
| GCTCCGTTTCCGACCTGGGCCGGTTCACCCCTCCTTAGGCAACCTGGTGG | 57043 | 0.10372881099502484 | No Hit |
| GGCCTATCAACCTCGTAGTCTACAAGGAATTTCAAGGGAATACTCATCTT | 56509 | 0.10275776835927035 | No Hit |

## Adapter Content

Produced by FastQC (version 0.11.7)
