# Supplementary material for: Variation of Long Non-Coding RNA And mRNA Profiles in Breast Cancer Cells With Influences of Adipocytes
Source: Front Oncol. 2021 May 21;11:631551. doi: 10.3389/fonc.2021.631551 (PMC8176020; doi:10.3389/fonc.2021.631551)
Supplement: Supplementary file 1 [file DataSheet_1.zip › sequencing/025G-201090513-CX-116_│┬╨π_6╚╦╤∙▒╛lncRNA_20190627/025G-201090513-CX-116_chenxiu_6╚╦╤∙▒╛lncRNA_20190627/1-Quality/clean/A3_clean_R2_fastqc/fastqc_report.html]

A3\_clean\_R2.fastq.gz FastQC Report 

FastQC Report

星期二 23 七月 2019  
A3\_clean\_R2.fastq.gz

## Summary

- Basic Statistics
- Per base sequence quality
- Per tile sequence quality
- Per sequence quality scores
- Per base sequence content
- Per sequence GC content
- Per base N content
- Sequence Length Distribution
- Sequence Duplication Levels
- Overrepresented sequences
- Adapter Content

## Basic Statistics

| Measure | Value |
| --- | --- |
| Filename | A3\_clean\_R2.fastq.gz |
| File type | Conventional base calls |
| Encoding | Sanger / Illumina 1.9 |
| Total Sequences | 54992436 |
| Sequences flagged as poor quality | 0 |
| Sequence length | 40-150 |
| %GC | 48 |

## Per base sequence quality

## Per tile sequence quality

## Per sequence quality scores

## Per base sequence content

## Per sequence GC content

## Per base N content

## Sequence Length Distribution

## Sequence Duplication Levels

## Overrepresented sequences

| Sequence | Count | Percentage | Possible Source |
| --- | --- | --- | --- |
| CCTTACCCACTCTTGACATCCTTCGCAAAGCTATAGAGATATAGTGGAGG | 191673 | 0.3485442979830899 | No Hit |
| ACCTTACCCACTCTTGACATCCTTCGCAAAGCTATAGAGATATAGTGGAG | 191004 | 0.3473277670405435 | No Hit |
| CTTACCCACTCTTGACATCCTTCGCAAAGCTATAGAGATATAGTGGAGGT | 146760 | 0.2668730659612897 | No Hit |
| GTGAAATAGATTCTGAAACTACTTACTTACAATTAGTCAGAGCACGTTAA | 129609 | 0.23568514040730984 | No Hit |
| GTCGAAGAAGGACGTGATTACCTGCGATAAGCCTCGTTTAGTTGGATATA | 121598 | 0.22111768243909036 | No Hit |
| GGAAGTCGAAGAAGGACGTGATTACCTGCGATAAGCCTCGTTTAGTTGGA | 121088 | 0.22019028216898776 | No Hit |
| CTTGGGTCTGGAAGTCGAAGAAGGACGTGATTACCTGCGATAAGCCTCGT | 117889 | 0.21437311851397164 | No Hit |
| GAAATAGATTCTGAAACTACTTACTTACAATTAGTCAGAGCACGTTAATG | 107026 | 0.19461949276078624 | No Hit |
| GCCTAATACATGCATGTCGAGCGGAGTATTAATTTATTAGTGCTTAGCGG | 95469 | 0.17360387526750043 | No Hit |
| GATGAACGCTGGCTGTGTGCCTAATACATGCATGTCGAGCGGAGTATTAA | 95352 | 0.17339111873494747 | No Hit |
| GAACGCTGGCTGTGTGCCTAATACATGCATGTCGAGCGGAGTATTAATTT | 94504 | 0.17184908848191413 | No Hit |
| GTGGGGATGACGTCAAATCATCATGCCTCTTACGAGTGGGGCAACACACG | 91566 | 0.16650653555336228 | No Hit |
| GTTCGGTTAAGTCCTGCAACGAGCGCAACCCCTATCTTTAATTACTACAT | 89074 | 0.16197500325317468 | No Hit |
| GAGTGAAATAGATTCTGAAACTACTTACTTACAATTAGTCAGAGCACGTT | 76391 | 0.1389118314380545 | No Hit |
| CAACCCTGGCCCGCTTTTGATACTAGCAAACTAGAGTTATAAAGAGGTTA | 74605 | 0.13566411206079323 | No Hit |
| CCACTCTTGACATCCTTCGCAAAGCTATAGAGATATAGTGGAGGTTAACG | 74040 | 0.13463669803607173 | No Hit |
| GTGGATGCCTTGGGTCTGGAAGTCGAAGAAGGACGTGATTACCTGCGATA | 71896 | 0.13073798003783646 | No Hit |
| CACGTATTTAACCTACCTCAAAGACTGGGATAACAACAGGAAACTGTTGC | 70841 | 0.1288195343810556 | No Hit |
| GGACAGTCTACATAGAGTTACAAAGATTATGTATAGTAGAAAAAGCTGGG | 70452 | 0.1281121643711146 | No Hit |
| GCTGGCTGTGTGCCTAATACATGCATGTCGAGCGGAGTATTAATTTATTA | 70192 | 0.1276393720765525 | No Hit |
| CCTTCGCAAAGCTATAGAGATATAGTGGAGGTTAACGGAGTGACAGATGG | 69392 | 0.12618462655482293 | No Hit |
| AAGGTAGCGAAATTCCTTGTCGGCTAAATACTGACCTGCACGAAAGACGC | 69349 | 0.12610643398302995 | No Hit |
| GTCGCCTCCCAAAAGGTAACGGAGGCGTTCAAAGCTACACTCAATATGGT | 68417 | 0.12441165545021501 | No Hit |
| CGCCTCCCAAAAGGTAACGGAGGCGTTCAAAGCTACACTCAATATGGTCA | 67244 | 0.12227863482897902 | No Hit |
| AGAACCTTACCCACTCTTGACATCCTTCGCAAAGCTATAGAGATATAGTG | 66167 | 0.12032018367035058 | No Hit |
| GGAATTCCTAGTGAAGCGGTGAAATGTGTAGATATTAGGAAGAACATCAA | 65374 | 0.11887816717193617 | No Hit |
| GCGGTGGCGCGTGCCTGTAGTCCCAGCTACTCGGGAGGCTGAGGCTGGAG | 65215 | 0.1185890364994924 | No Hit |
| GCGGTGGCGCGTGCCTGTAGTCCCAGCTACTCGGGAGGCTGAGGTGGGAG | 64040 | 0.1164523790144521 | No Hit |
| GTTAAATCCCGGGGCTCAACCCTGGCCCGCTTTTGATACTAGCAAACTAG | 62869 | 0.11432299525702043 | No Hit |
| GTCTGATGGAGTGACACAGCGTGCAGGATGAAGGTCTTCGGATTGTAAAC | 62652 | 0.11392839553425128 | No Hit |
| ATTTGATGTGGGCGTTGGAATATTGATGAGAGCTGCTCTTAGTACGAGAG | 58606 | 0.106571020058104 | No Hit |
| GCGTTATCCGAAATTATTGGGTGTAAAGAGTTCGTAGGTTGTTTGTTAAG | 56600 | 0.10292324566236709 | No Hit |
| GGTGGCGCGTGCCTGTAGTCCCAGCTACTCGGGAGGCTGAGGTGGGAGGA | 56537 | 0.1028086844525309 | No Hit |
| GCGAAATTCCTTGTCGGCTAAATACTGACCTGCACGAAAGACGCAATGAT | 56343 | 0.10245590866351147 | No Hit |
| GGTGGCGCGTGCCTGTAGTCCCAGCTACTCGGGAGGCTGAGGCTGGAGGA | 55994 | 0.10182127592965695 | No Hit |
| GGTAGCGAAATTCCTTGTCGGCTAAATACTGACCTGCACGAAAGACGCAA | 55957 | 0.10175399394927694 | No Hit |

## Adapter Content

Produced by FastQC (version 0.11.7)
