# Supplementary material for: Variation of Long Non-Coding RNA And mRNA Profiles in Breast Cancer Cells With Influences of Adipocytes
Source: Front Oncol. 2021 May 21;11:631551. doi: 10.3389/fonc.2021.631551 (PMC8176020; doi:10.3389/fonc.2021.631551)
Supplement: Supplementary file 1 [file DataSheet_1.zip › sequencing/025G-201090513-CX-116_│┬╨π_6╚╦╤∙▒╛lncRNA_20190627/025G-201090513-CX-116_chenxiu_6╚╦╤∙▒╛lncRNA_20190627/1-Quality/clean/B1_clean_R1_fastqc/fastqc_report.html]

B1\_clean\_R1.fastq.gz FastQC Report 

FastQC Report

星期一 22 七月 2019  
B1\_clean\_R1.fastq.gz

## Summary

- Basic Statistics
- Per base sequence quality
- Per tile sequence quality
- Per sequence quality scores
- Per base sequence content
- Per sequence GC content
- Per base N content
- Sequence Length Distribution
- Sequence Duplication Levels
- Overrepresented sequences
- Adapter Content

## Basic Statistics

| Measure | Value |
| --- | --- |
| Filename | B1\_clean\_R1.fastq.gz |
| File type | Conventional base calls |
| Encoding | Sanger / Illumina 1.9 |
| Total Sequences | 52270051 |
| Sequences flagged as poor quality | 0 |
| Sequence length | 40-150 |
| %GC | 48 |

## Per base sequence quality

## Per tile sequence quality

## Per sequence quality scores

## Per base sequence content

## Per sequence GC content

## Per base N content

## Sequence Length Distribution

## Sequence Duplication Levels

## Overrepresented sequences

| Sequence | Count | Percentage | Possible Source |
| --- | --- | --- | --- |
| CCGAGAACGTATTCACCGTAGCGTAGCTGATCTACGATTACTAGCGATTC | 486344 | 0.9304448545496924 | No Hit |
| CGAGAACGTATTCACCGTAGCGTAGCTGATCTACGATTACTAGCGATTCC | 273916 | 0.5240400473303537 | No Hit |
| GTCTGATTAGTATTTAGCCTTACCGGGTGGTCCCGGCAGATTCAGACAGG | 207812 | 0.39757374638873033 | No Hit |
| ATTCAGGCGGATCATTTAACGCGTTAGCTGCGTTAGTGAAATTATTCCAC | 204712 | 0.3916430079626285 | No Hit |
| GGCGGATCATTTAACGCGTTAGCTGCGTTAGTGAAATTATTCCACCAACT | 181603 | 0.3474322227081814 | No Hit |
| CCCATTTTTAAGTGAAGCTGTGAAGCTCCTTTCTATTACTCATCATGCGA | 178169 | 0.34086249504520283 | No Hit |
| GTCCCTTAGTGTCAATATATAACCAGTTAGCTGCCTTCGCCTATTGGTGT | 113908 | 0.21792211375496842 | No Hit |
| GTCTGGAGTCTTGGAAGCTTGACTACCCTACGTTCTCCTACAAATGGACC | 106500 | 0.20374956205801292 | No Hit |
| CTGATTAGTATTTAGCCTTACCGGGTGGTCCCGGCAGATTCAGACAGGGT | 105547 | 0.20192633827734358 | No Hit |
| CTGGAGTCTTGGAAGCTTGACTACCCTACGTTCTCCTACAAATGGACCTT | 97265 | 0.18608170097251292 | No Hit |
| CCAGGCTGGAGTGCAGTGGCTATTCACAGGCGCGATCCCACTACTGATCA | 97079 | 0.18572585666694683 | No Hit |
| CCGGATAACGCTTGCGACCTATGTATTACCGCGGCTGCTGGCACATAGTT | 86790 | 0.16604154451657221 | No Hit |
| CCTTAGTGTCAATATATAACCAGTTAGCTGCCTTCGCCTATTGGTGTTCT | 85659 | 0.16387778156175895 | No Hit |
| CACGTGTGTTGCCCCACTCGTAAGAGGCATGATGATTTGACGTCGTCCCC | 85404 | 0.16338993049767636 | No Hit |
| GCTCAGGCTGGAGTGCAGTGGCTATTCACAGGCGCGATCCCACTACTGAT | 83134 | 0.15904709945662765 | No Hit |
| CCGGCATTCTCACTTTTAATCTCTCCACCAGTCCTCACGGTCTGACTTCA | 80277 | 0.15358125439747514 | No Hit |
| CCCCTCCTTAGGCAACCTGGTGGTCCCCCGCTCCCGGGAGGTCACCATAT | 77082 | 0.14746876753573476 | No Hit |
| GTGGCTATTCACAGGCGCGATCCCACTACTGATCAGCACGGGAGTTTTGA | 76783 | 0.1468967382488301 | No Hit |
| GTCCCCACCTTCCTCCTGGTTACCCAGGCAGTATCTCTAGAGTCCTTAAC | 70879 | 0.13560155125924786 | No Hit |
| GGCTGGAGTGCAGTGGCTATTCACAGGCGCGATCCCACTACTGATCAGCA | 70018 | 0.1339543364899338 | No Hit |
| CCACAATCCAGTAAGTGGTAGAACTATCCTTTTTCGTCACTCCATCATTC | 67978 | 0.13005152797727326 | No Hit |
| GCTCCGTTTCCGACCTGGGCCGGTTCACCCCTCCTTAGGCAACCTGGTGG | 67765 | 0.12964402885315723 | No Hit |
| CTCCGTTTAACCTTCGGGCACTGGGCAGGCTTCACCCTCTATACGTCGTT | 65846 | 0.12597271045325745 | No Hit |
| GCCCAGGCTGGAGTGCAGTGGCTATTCACAGGCGCGATCCCACTACTGAT | 63068 | 0.12065800356689915 | No Hit |
| CCCCCATTAAACAATACTATACGCTAGCCCTAAAGCTATTTCGAAGAGAA | 62151 | 0.11890365287762968 | No Hit |
| GGGGTCTTGTCGTCTTGATGCGGGTAACCAGCGTTTTCACTGGTACCATA | 62019 | 0.1186511182091634 | No Hit |
| CCATTTTTAAGTGAAGCTGTGAAGCTCCTTTCTATTACTCATCATGCGAT | 61746 | 0.1181288305993809 | No Hit |
| CCCTAGAGTACCTTTTATCCGTTGAGCGATGGCCCTTCCATACAGAACCA | 61517 | 0.11769072121242047 | No Hit |
| CAGGCGGATCATTTAACGCGTTAGCTGCGTTAGTGAAATTATTCCACCAA | 59421 | 0.11368077677980455 | No Hit |
| CTTAGTGTCAATATATAACCAGTTAGCTGCCTTCGCCTATTGGTGTTCTT | 59148 | 0.11315848917002205 | No Hit |
| CCCTCCTTAGGCAACCTGGTGGTCCCCCGCTCCCGGGAGGTCACCATATT | 59010 | 0.11289447565298913 | No Hit |
| AGCACGTGTGTTGCCCCACTCGTAAGAGGCATGATGATTTGACGTCGTCC | 56315 | 0.10773855950513613 | No Hit |
| GCCGTATCTCAGTCCCAGTGTGGCCGTACAGCCTCTCGGCCCGGCTAAAC | 56158 | 0.1074381963009755 | No Hit |
| AGGCGGATCATTTAACGCGTTAGCTGCGTTAGTGAAATTATTCCACCAAC | 55760 | 0.10667676601272114 | No Hit |
| GCTCCATGTCACCATATTGCTTCTCTTTGTACCGACCATTGTAGCACGTG | 55625 | 0.10641849191997153 | No Hit |
| GCTCCACGGGGTCTTGTCGTCTTGATGCGGGTAACCAGCGTTTTCACTGG | 54092 | 0.10348564611119282 | No Hit |
| CCTCTTTATAATTCTATTTTGCCAGTATCCAAAGCGGACTGAAGTTGAGC | 54036 | 0.10337851019123744 | No Hit |
| GCGGATCATTTAACGCGTTAGCTGCGTTAGTGAAATTATTCCACCAACTA | 54025 | 0.1033574656355319 | No Hit |
| CTCCATCATTCTTTTACCAAGTACAGGAATATTAACCTGTTGTCCATCGA | 53437 | 0.10223253847600033 | No Hit |
| GGGACCTTAGCTGACGATCTGGGTTGTTTCCCTCGCGAGCGTGGACGTTA | 53342 | 0.10205079004036173 | No Hit |
| GCACGTGTGTTGCCCCACTCGTAAGAGGCATGATGATTTGACGTCGTCCC | 53259 | 0.10189199930185644 | No Hit |
| CCTCCTTAGGCAACCTGGTGGTCCCCCGCTCCCGGGAGGTCACCATATTG | 52545 | 0.10052601632242525 | No Hit |

## Adapter Content

Produced by FastQC (version 0.11.7)
