# Supplementary figures and images for: Variation of Long Non-Coding RNA And mRNA Profiles in Breast Cancer Cells With Influences of Adipocytes
Source: Front Oncol. 2021 May 21;11:631551. doi: 10.3389/fonc.2021.631551 (PMC8176020; doi:10.3389/fonc.2021.631551)

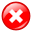

Supplement: Supplementary file 1 [file DataSheet_1.zip › sequencing/025G-201090513-CX-116_│┬╨π_6╚╦╤∙▒╛lncRNA_20190627/025G-201090513-CX-116_chenxiu_6╚╦╤∙▒╛lncRNA_20190627/1-Quality/clean/A1_clean_R1_fastqc/Icons/error.png]

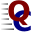

Supplement: Supplementary file 1 [file DataSheet_1.zip › sequencing/025G-201090513-CX-116_│┬╨π_6╚╦╤∙▒╛lncRNA_20190627/025G-201090513-CX-116_chenxiu_6╚╦╤∙▒╛lncRNA_20190627/1-Quality/clean/A1_clean_R1_fastqc/Icons/fastqc_icon.png]

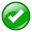

Supplement: Supplementary file 1 [file DataSheet_1.zip › sequencing/025G-201090513-CX-116_│┬╨π_6╚╦╤∙▒╛lncRNA_20190627/025G-201090513-CX-116_chenxiu_6╚╦╤∙▒╛lncRNA_20190627/1-Quality/clean/A1_clean_R1_fastqc/Icons/tick.png]

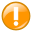

Supplement: Supplementary file 1 [file DataSheet_1.zip › sequencing/025G-201090513-CX-116_│┬╨π_6╚╦╤∙▒╛lncRNA_20190627/025G-201090513-CX-116_chenxiu_6╚╦╤∙▒╛lncRNA_20190627/1-Quality/clean/A1_clean_R1_fastqc/Icons/warning.png]

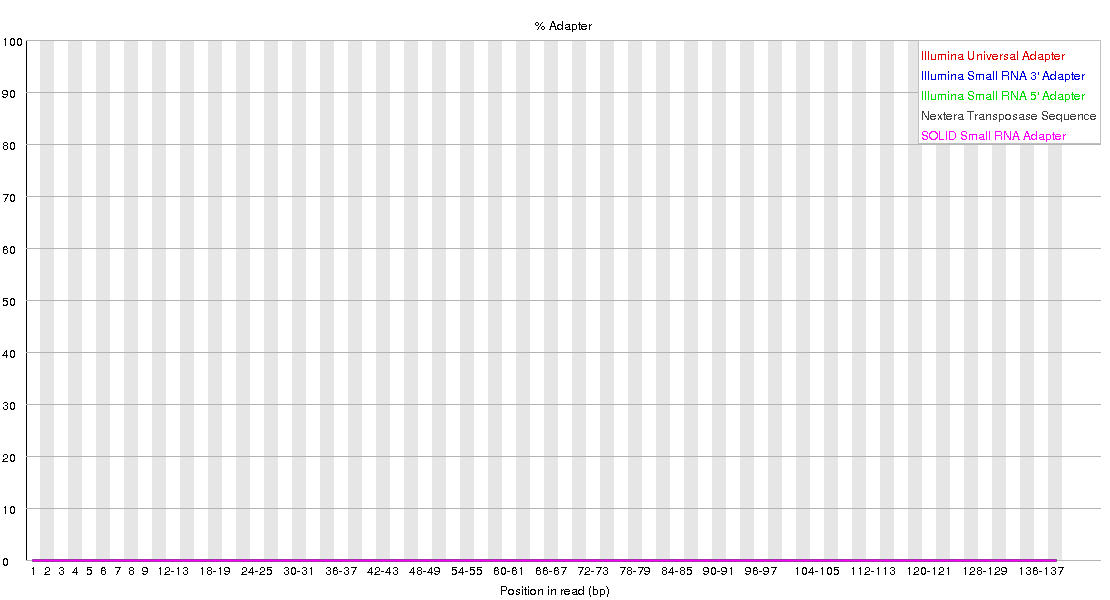

Supplement: Supplementary file 1 [file DataSheet_1.zip › sequencing/025G-201090513-CX-116_│┬╨π_6╚╦╤∙▒╛lncRNA_20190627/025G-201090513-CX-116_chenxiu_6╚╦╤∙▒╛lncRNA_20190627/1-Quality/clean/A1_clean_R1_fastqc/Images/adapter_content.png]

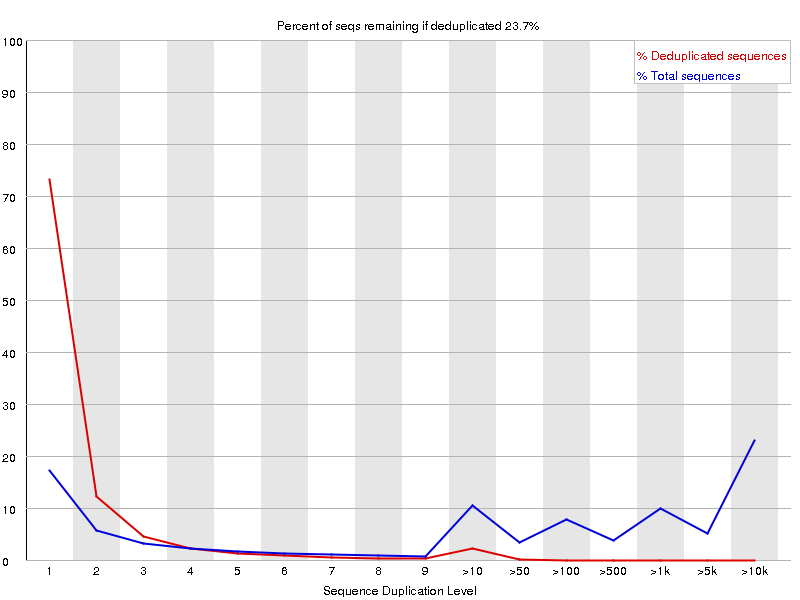

Supplement: Supplementary file 1 [file DataSheet_1.zip › sequencing/025G-201090513-CX-116_│┬╨π_6╚╦╤∙▒╛lncRNA_20190627/025G-201090513-CX-116_chenxiu_6╚╦╤∙▒╛lncRNA_20190627/1-Quality/clean/A1_clean_R1_fastqc/Images/duplication_levels.png]

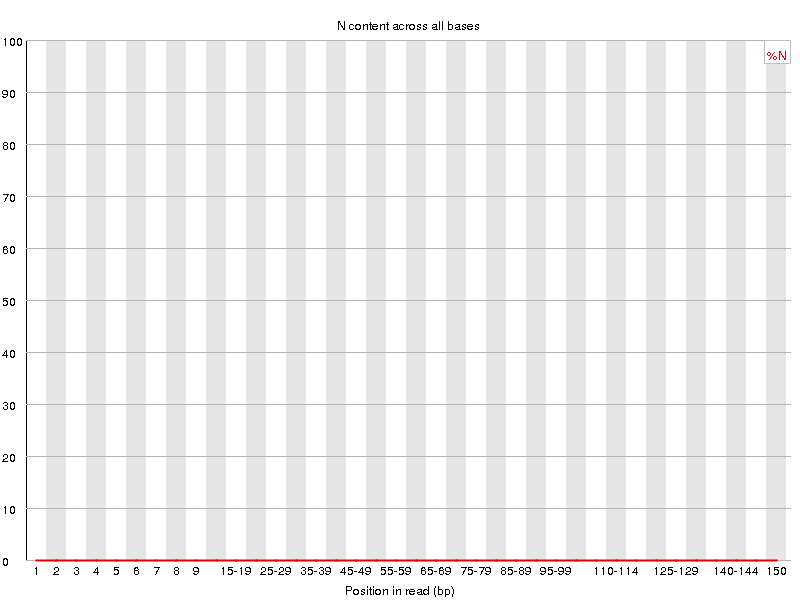

Supplement: Supplementary file 1 [file DataSheet_1.zip › sequencing/025G-201090513-CX-116_│┬╨π_6╚╦╤∙▒╛lncRNA_20190627/025G-201090513-CX-116_chenxiu_6╚╦╤∙▒╛lncRNA_20190627/1-Quality/clean/A1_clean_R1_fastqc/Images/per_base_n_content.png]

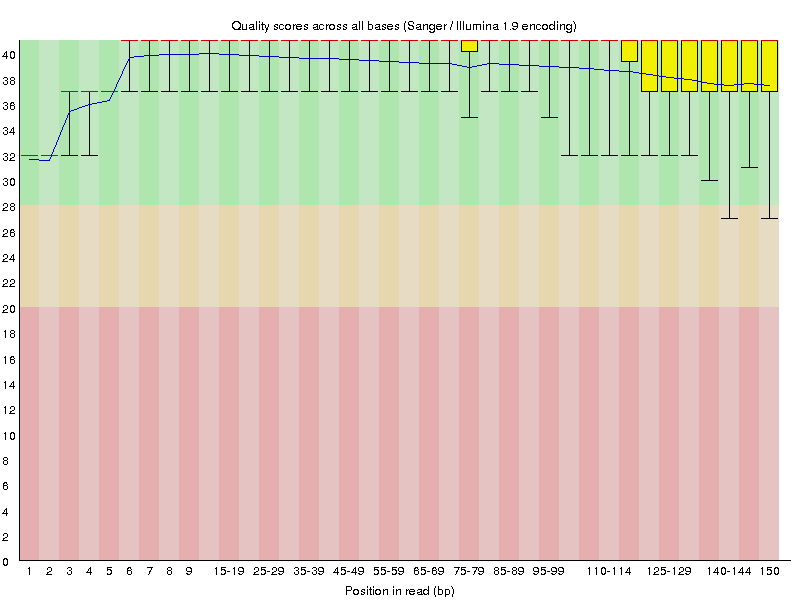

Supplement: Supplementary file 1 [file DataSheet_1.zip › sequencing/025G-201090513-CX-116_│┬╨π_6╚╦╤∙▒╛lncRNA_20190627/025G-201090513-CX-116_chenxiu_6╚╦╤∙▒╛lncRNA_20190627/1-Quality/clean/A1_clean_R1_fastqc/Images/per_base_quality.png]

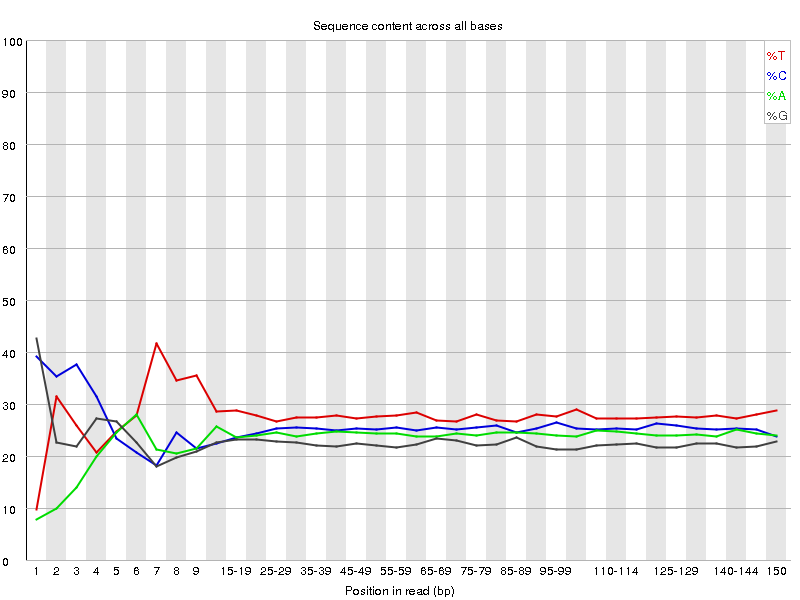

Supplement: Supplementary file 1 [file DataSheet_1.zip › sequencing/025G-201090513-CX-116_│┬╨π_6╚╦╤∙▒╛lncRNA_20190627/025G-201090513-CX-116_chenxiu_6╚╦╤∙▒╛lncRNA_20190627/1-Quality/clean/A1_clean_R1_fastqc/Images/per_base_sequence_content.png]

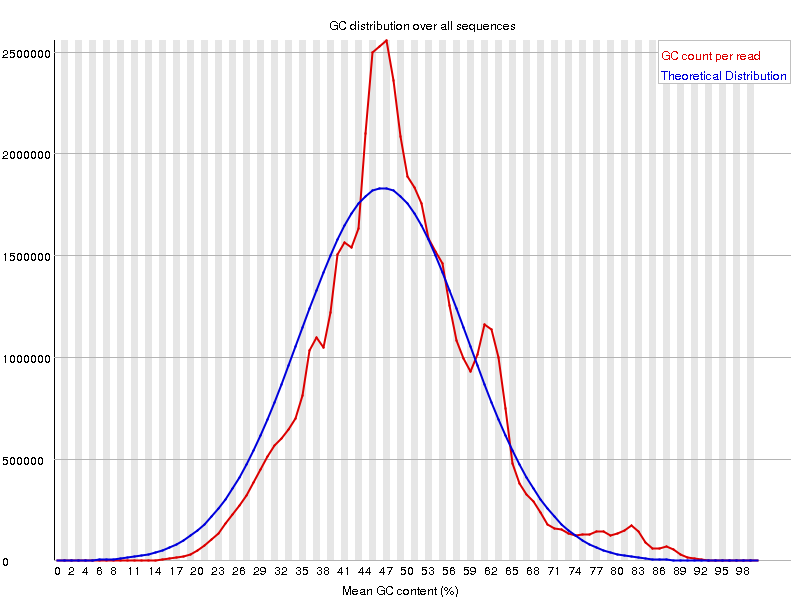

Supplement: Supplementary file 1 [file DataSheet_1.zip › sequencing/025G-201090513-CX-116_│┬╨π_6╚╦╤∙▒╛lncRNA_20190627/025G-201090513-CX-116_chenxiu_6╚╦╤∙▒╛lncRNA_20190627/1-Quality/clean/A1_clean_R1_fastqc/Images/per_sequence_gc_content.png]

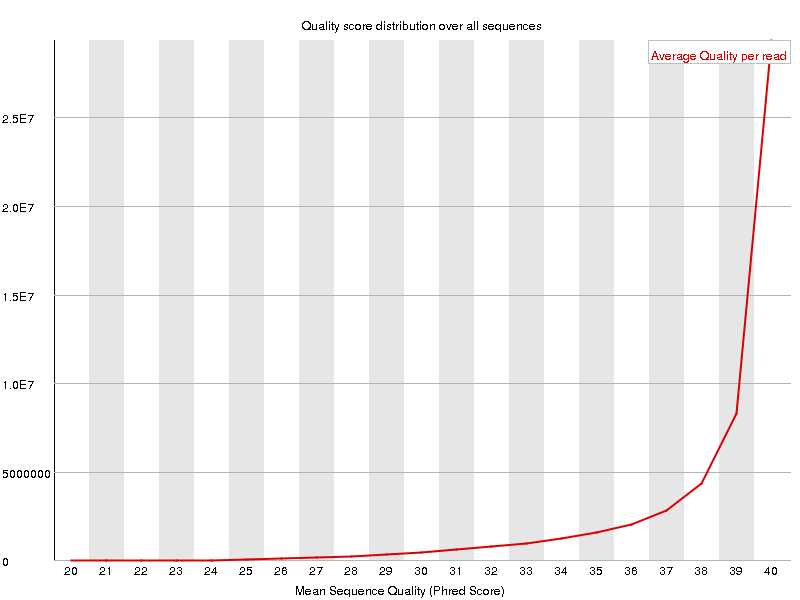

Supplement: Supplementary file 1 [file DataSheet_1.zip › sequencing/025G-201090513-CX-116_│┬╨π_6╚╦╤∙▒╛lncRNA_20190627/025G-201090513-CX-116_chenxiu_6╚╦╤∙▒╛lncRNA_20190627/1-Quality/clean/A1_clean_R1_fastqc/Images/per_sequence_quality.png]

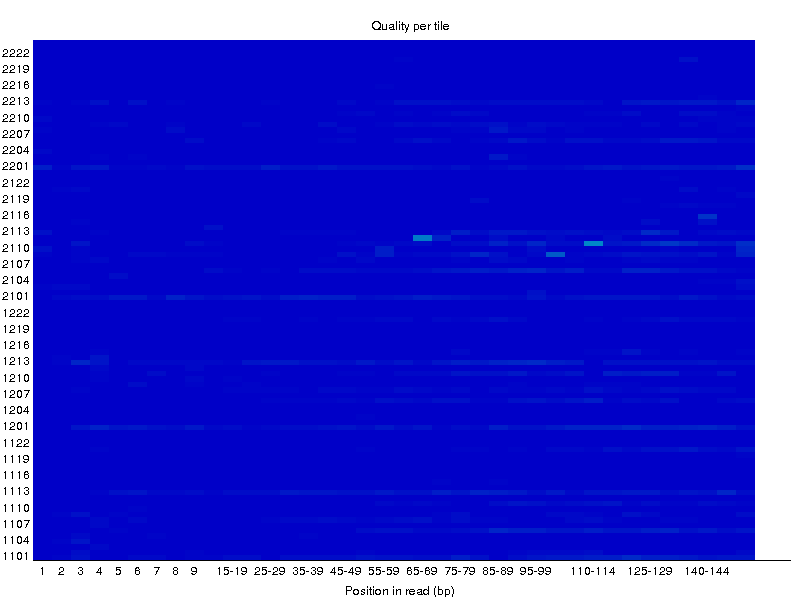

Supplement: Supplementary file 1 [file DataSheet_1.zip › sequencing/025G-201090513-CX-116_│┬╨π_6╚╦╤∙▒╛lncRNA_20190627/025G-201090513-CX-116_chenxiu_6╚╦╤∙▒╛lncRNA_20190627/1-Quality/clean/A1_clean_R1_fastqc/Images/per_tile_quality.png]

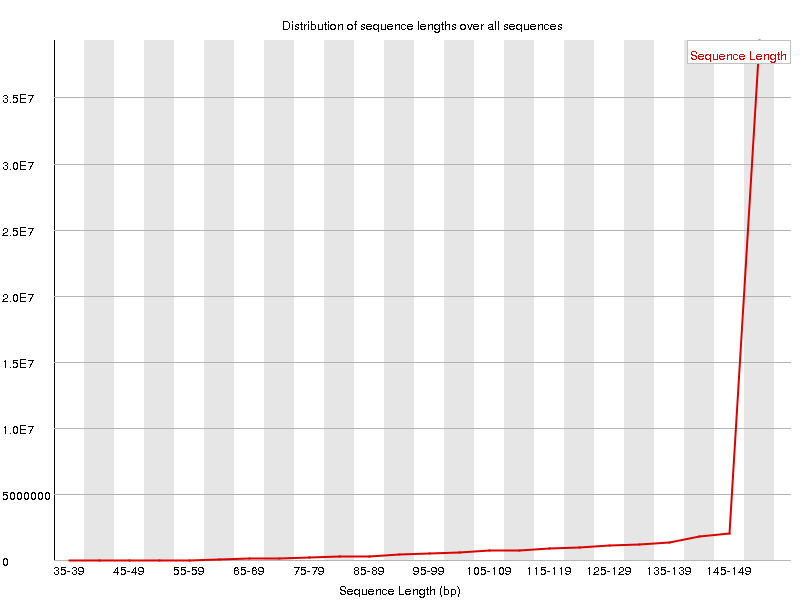

Supplement: Supplementary file 1 [file DataSheet_1.zip › sequencing/025G-201090513-CX-116_│┬╨π_6╚╦╤∙▒╛lncRNA_20190627/025G-201090513-CX-116_chenxiu_6╚╦╤∙▒╛lncRNA_20190627/1-Quality/clean/A1_clean_R1_fastqc/Images/sequence_length_distribution.png]

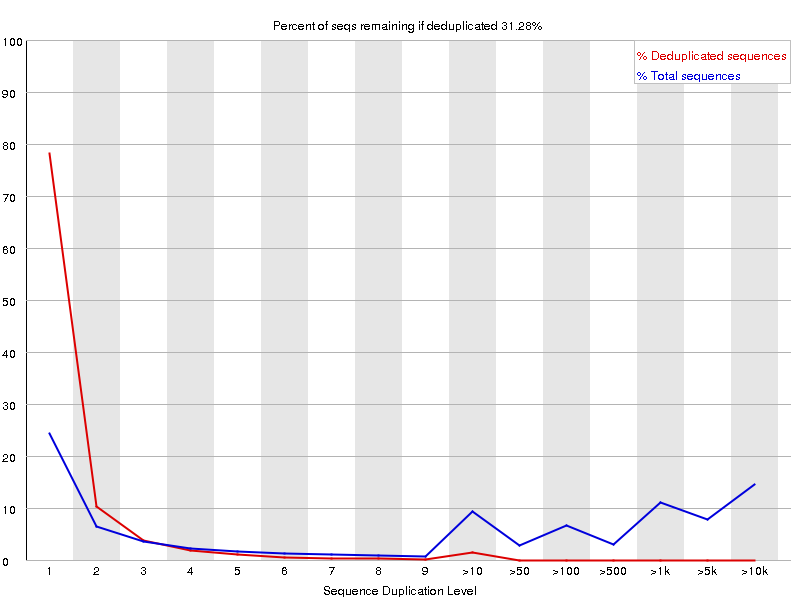

Supplement: Supplementary file 1 [file DataSheet_1.zip › sequencing/025G-201090513-CX-116_│┬╨π_6╚╦╤∙▒╛lncRNA_20190627/025G-201090513-CX-116_chenxiu_6╚╦╤∙▒╛lncRNA_20190627/1-Quality/clean/A1_clean_R2_fastqc/Images/duplication_levels.png]

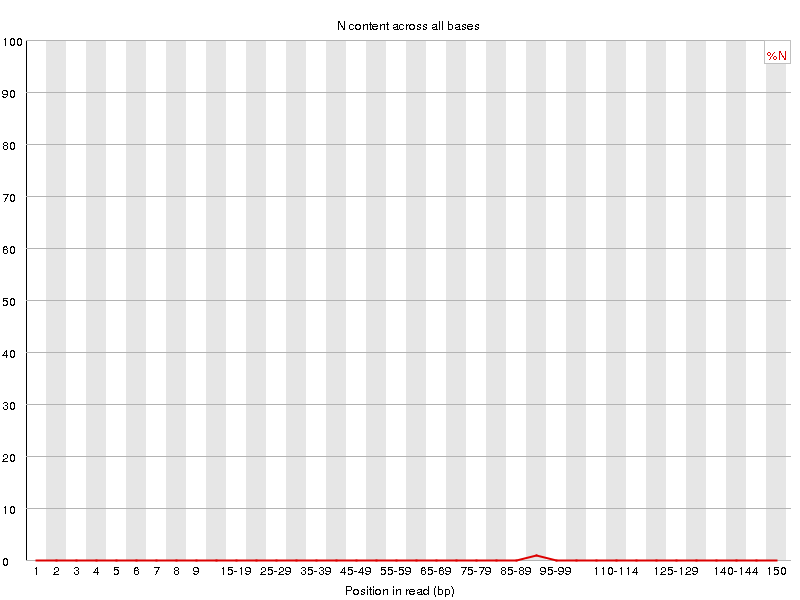

Supplement: Supplementary file 1 [file DataSheet_1.zip › sequencing/025G-201090513-CX-116_│┬╨π_6╚╦╤∙▒╛lncRNA_20190627/025G-201090513-CX-116_chenxiu_6╚╦╤∙▒╛lncRNA_20190627/1-Quality/clean/A1_clean_R2_fastqc/Images/per_base_n_content.png]

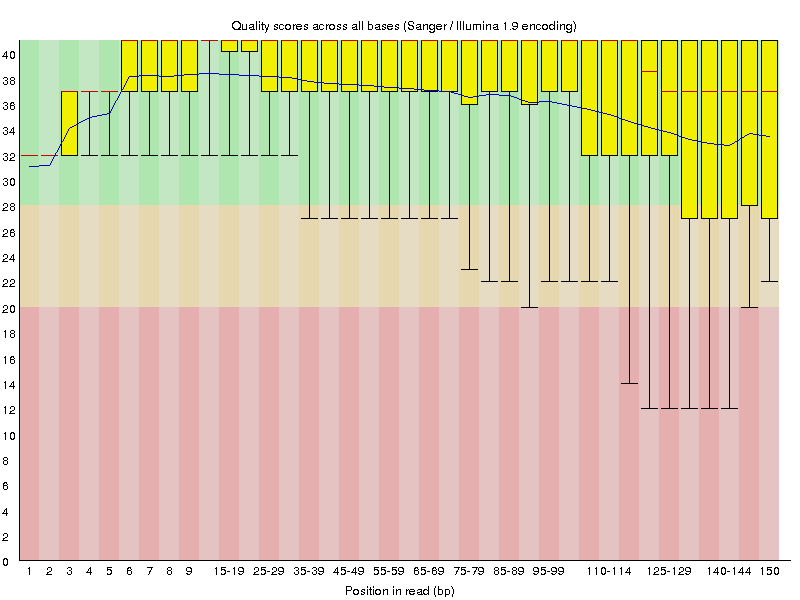

Supplement: Supplementary file 1 [file DataSheet_1.zip › sequencing/025G-201090513-CX-116_│┬╨π_6╚╦╤∙▒╛lncRNA_20190627/025G-201090513-CX-116_chenxiu_6╚╦╤∙▒╛lncRNA_20190627/1-Quality/clean/A1_clean_R2_fastqc/Images/per_base_quality.png]

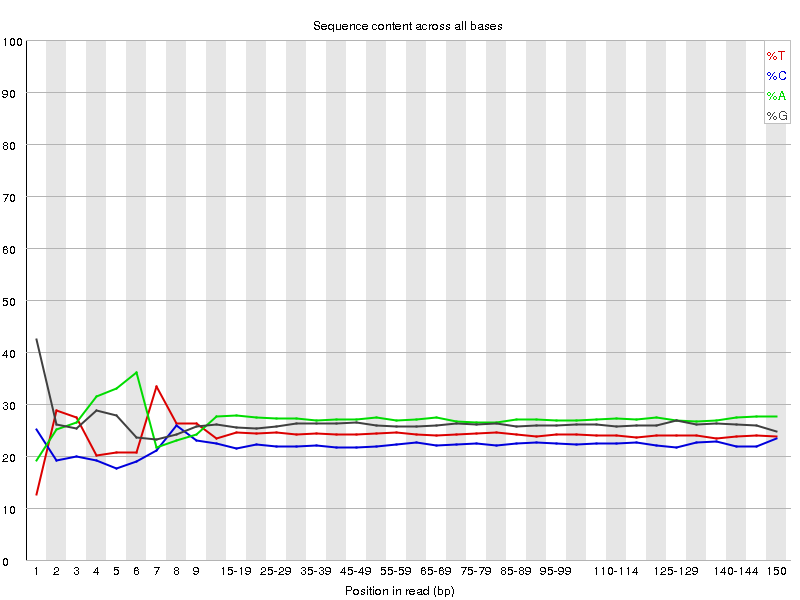

Supplement: Supplementary file 1 [file DataSheet_1.zip › sequencing/025G-201090513-CX-116_│┬╨π_6╚╦╤∙▒╛lncRNA_20190627/025G-201090513-CX-116_chenxiu_6╚╦╤∙▒╛lncRNA_20190627/1-Quality/clean/A1_clean_R2_fastqc/Images/per_base_sequence_content.png]

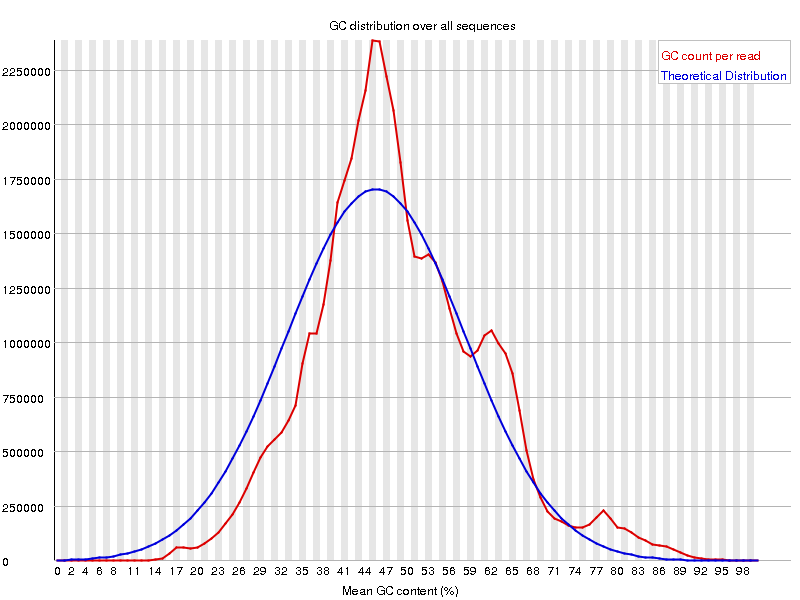

Supplement: Supplementary file 1 [file DataSheet_1.zip › sequencing/025G-201090513-CX-116_│┬╨π_6╚╦╤∙▒╛lncRNA_20190627/025G-201090513-CX-116_chenxiu_6╚╦╤∙▒╛lncRNA_20190627/1-Quality/clean/A1_clean_R2_fastqc/Images/per_sequence_gc_content.png]

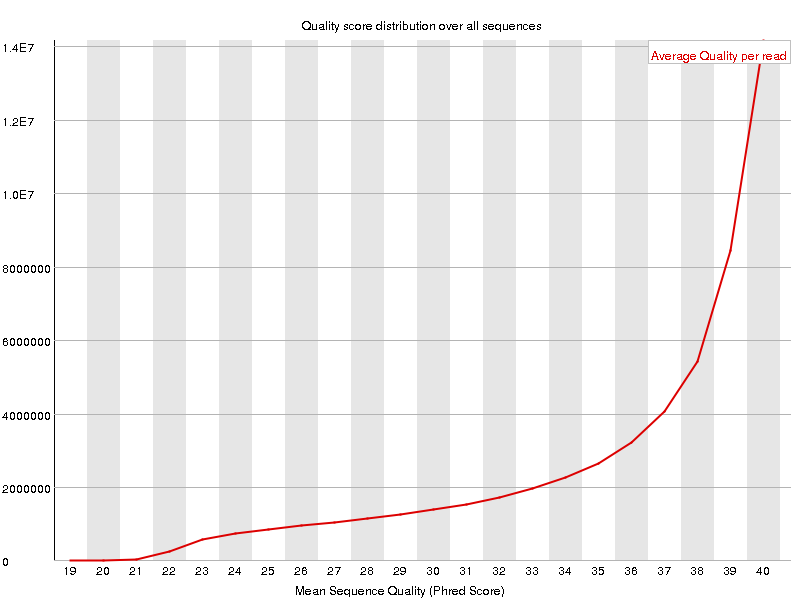

Supplement: Supplementary file 1 [file DataSheet_1.zip › sequencing/025G-201090513-CX-116_│┬╨π_6╚╦╤∙▒╛lncRNA_20190627/025G-201090513-CX-116_chenxiu_6╚╦╤∙▒╛lncRNA_20190627/1-Quality/clean/A1_clean_R2_fastqc/Images/per_sequence_quality.png]

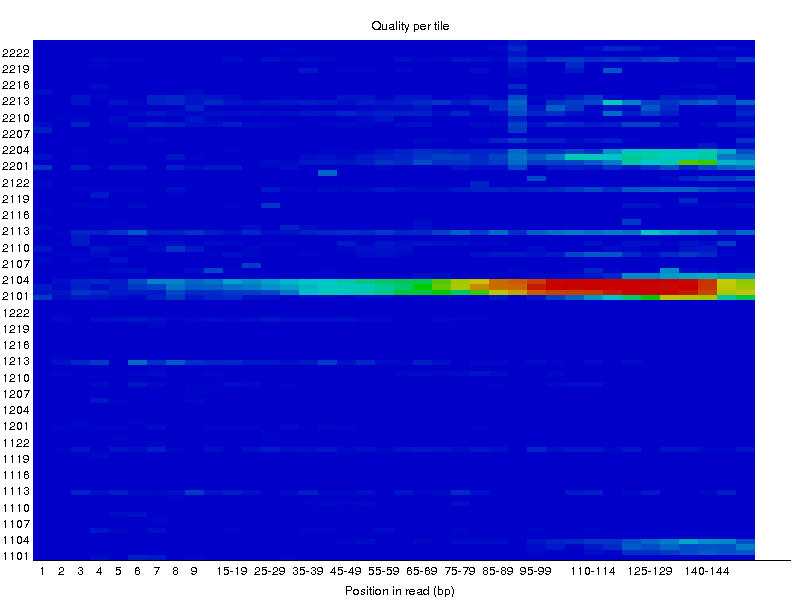

Supplement: Supplementary file 1 [file DataSheet_1.zip › sequencing/025G-201090513-CX-116_│┬╨π_6╚╦╤∙▒╛lncRNA_20190627/025G-201090513-CX-116_chenxiu_6╚╦╤∙▒╛lncRNA_20190627/1-Quality/clean/A1_clean_R2_fastqc/Images/per_tile_quality.png]

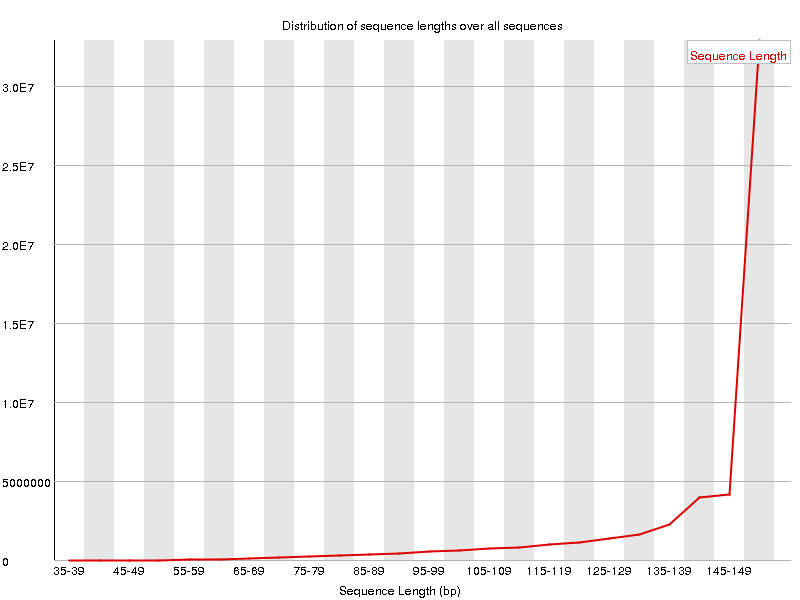

Supplement: Supplementary file 1 [file DataSheet_1.zip › sequencing/025G-201090513-CX-116_│┬╨π_6╚╦╤∙▒╛lncRNA_20190627/025G-201090513-CX-116_chenxiu_6╚╦╤∙▒╛lncRNA_20190627/1-Quality/clean/A1_clean_R2_fastqc/Images/sequence_length_distribution.png]

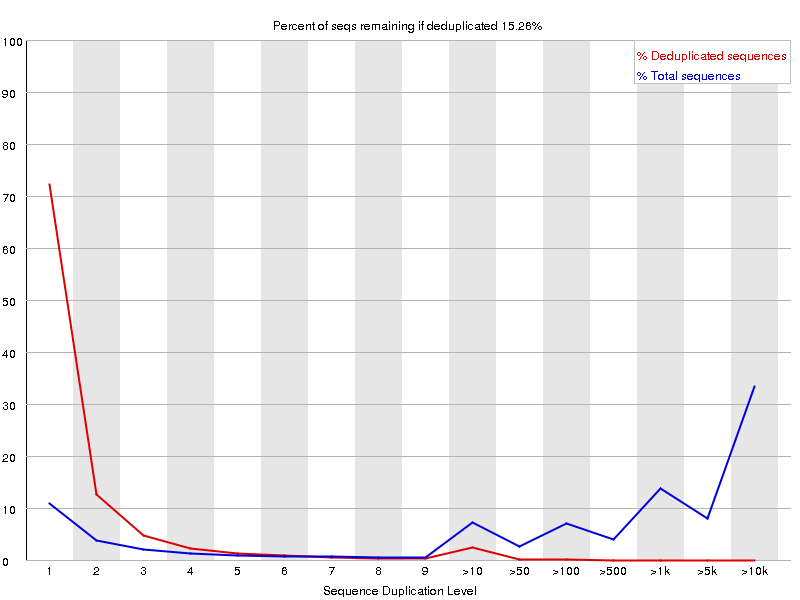

Supplement: Supplementary file 1 [file DataSheet_1.zip › sequencing/025G-201090513-CX-116_│┬╨π_6╚╦╤∙▒╛lncRNA_20190627/025G-201090513-CX-116_chenxiu_6╚╦╤∙▒╛lncRNA_20190627/1-Quality/clean/A2_clean_R1_fastqc/Images/duplication_levels.png]

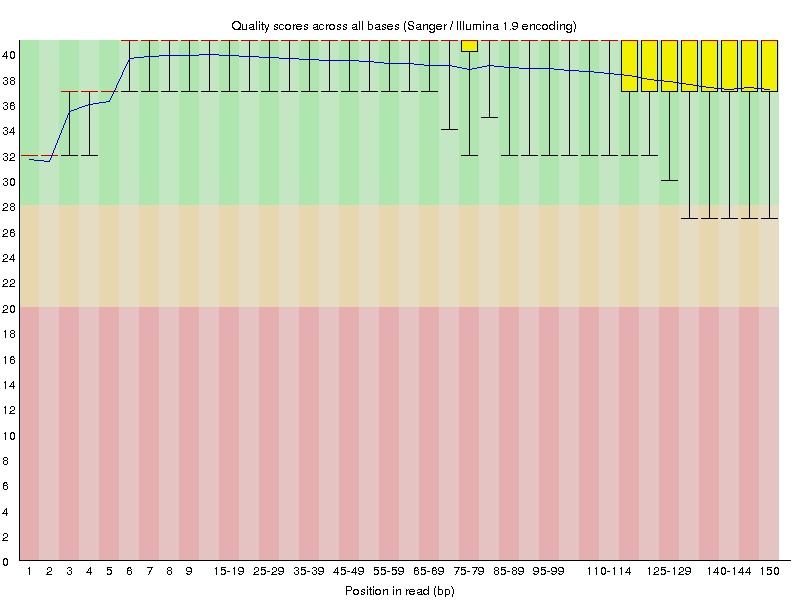

Supplement: Supplementary file 1 [file DataSheet_1.zip › sequencing/025G-201090513-CX-116_│┬╨π_6╚╦╤∙▒╛lncRNA_20190627/025G-201090513-CX-116_chenxiu_6╚╦╤∙▒╛lncRNA_20190627/1-Quality/clean/A2_clean_R1_fastqc/Images/per_base_quality.png]

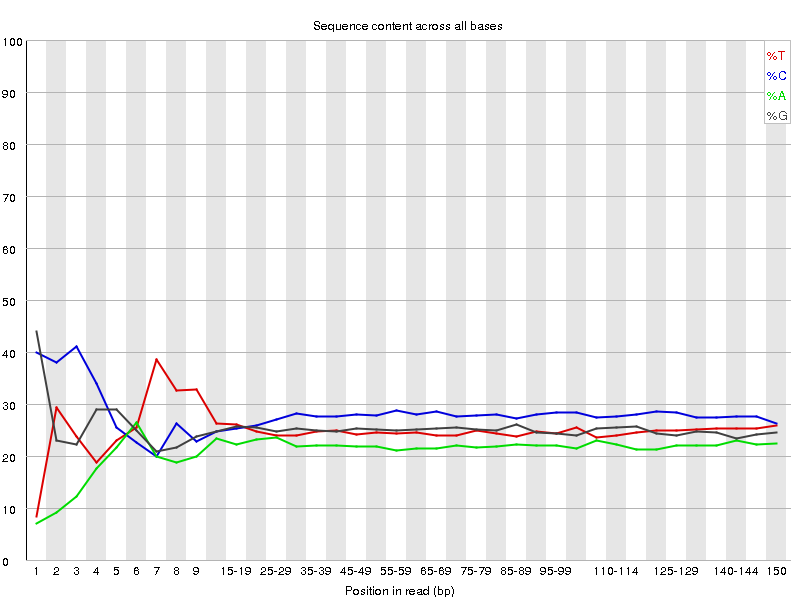

Supplement: Supplementary file 1 [file DataSheet_1.zip › sequencing/025G-201090513-CX-116_│┬╨π_6╚╦╤∙▒╛lncRNA_20190627/025G-201090513-CX-116_chenxiu_6╚╦╤∙▒╛lncRNA_20190627/1-Quality/clean/A2_clean_R1_fastqc/Images/per_base_sequence_content.png]

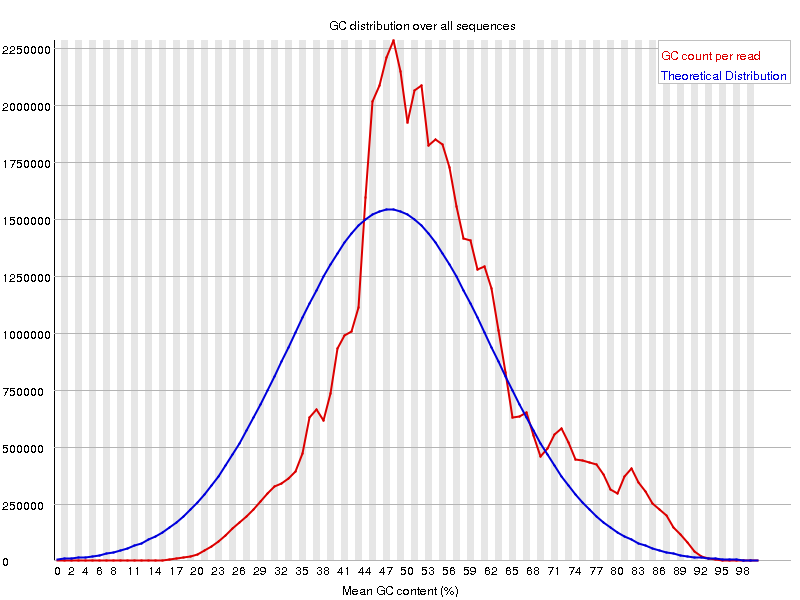

Supplement: Supplementary file 1 [file DataSheet_1.zip › sequencing/025G-201090513-CX-116_│┬╨π_6╚╦╤∙▒╛lncRNA_20190627/025G-201090513-CX-116_chenxiu_6╚╦╤∙▒╛lncRNA_20190627/1-Quality/clean/A2_clean_R1_fastqc/Images/per_sequence_gc_content.png]

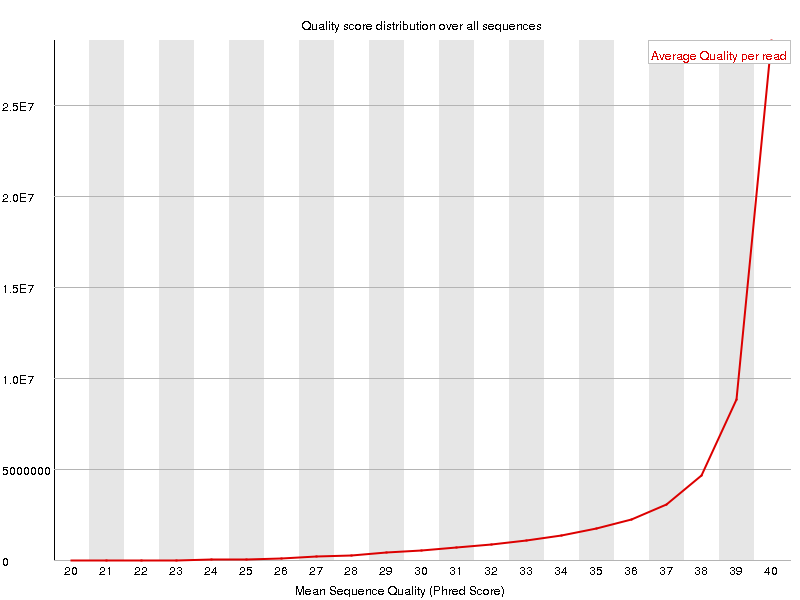

Supplement: Supplementary file 1 [file DataSheet_1.zip › sequencing/025G-201090513-CX-116_│┬╨π_6╚╦╤∙▒╛lncRNA_20190627/025G-201090513-CX-116_chenxiu_6╚╦╤∙▒╛lncRNA_20190627/1-Quality/clean/A2_clean_R1_fastqc/Images/per_sequence_quality.png]

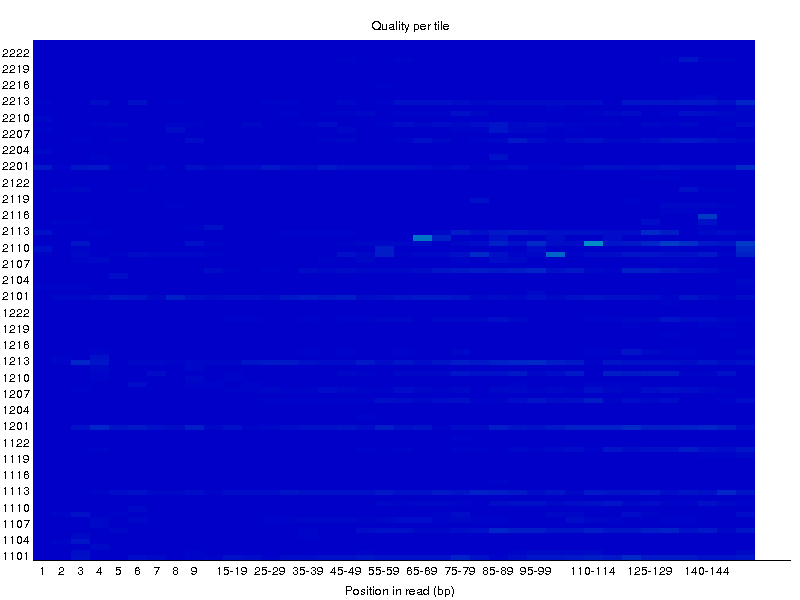

Supplement: Supplementary file 1 [file DataSheet_1.zip › sequencing/025G-201090513-CX-116_│┬╨π_6╚╦╤∙▒╛lncRNA_20190627/025G-201090513-CX-116_chenxiu_6╚╦╤∙▒╛lncRNA_20190627/1-Quality/clean/A2_clean_R1_fastqc/Images/per_tile_quality.png]

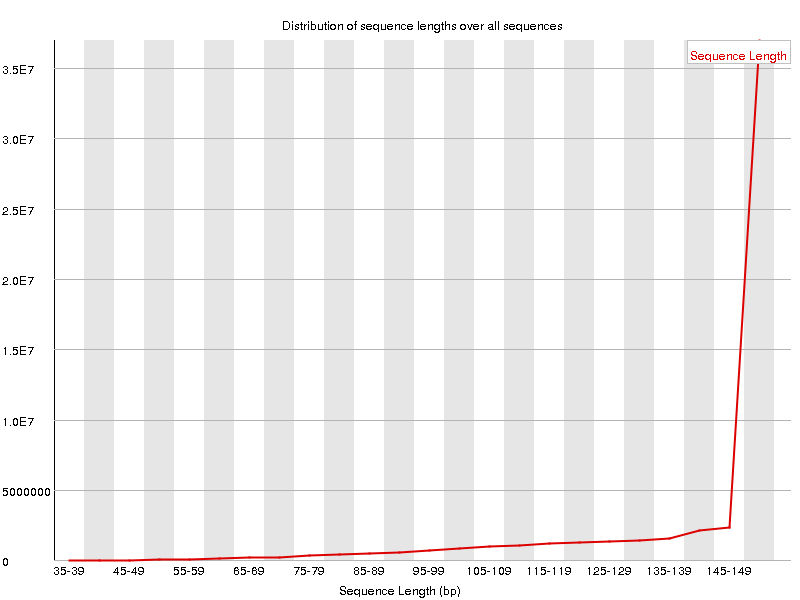

Supplement: Supplementary file 1 [file DataSheet_1.zip › sequencing/025G-201090513-CX-116_│┬╨π_6╚╦╤∙▒╛lncRNA_20190627/025G-201090513-CX-116_chenxiu_6╚╦╤∙▒╛lncRNA_20190627/1-Quality/clean/A2_clean_R1_fastqc/Images/sequence_length_distribution.png]

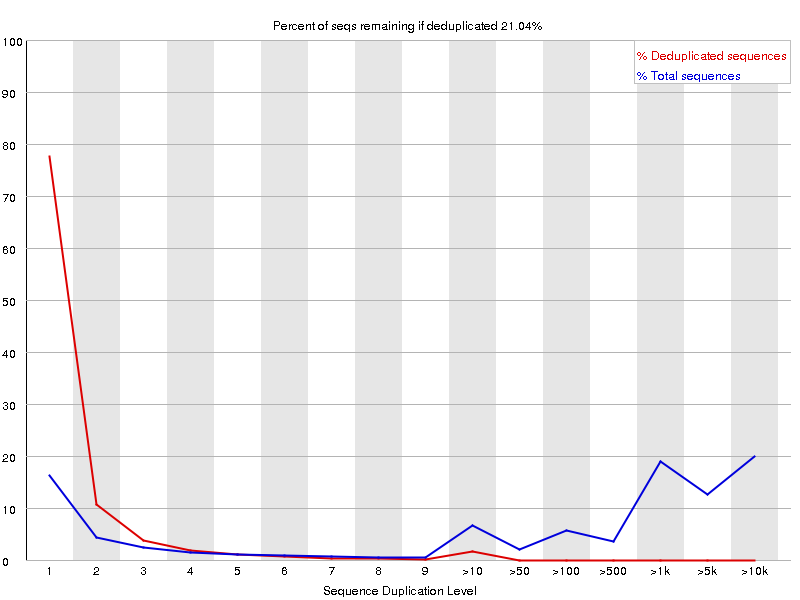

Supplement: Supplementary file 1 [file DataSheet_1.zip › sequencing/025G-201090513-CX-116_│┬╨π_6╚╦╤∙▒╛lncRNA_20190627/025G-201090513-CX-116_chenxiu_6╚╦╤∙▒╛lncRNA_20190627/1-Quality/clean/A2_clean_R2_fastqc/Images/duplication_levels.png]

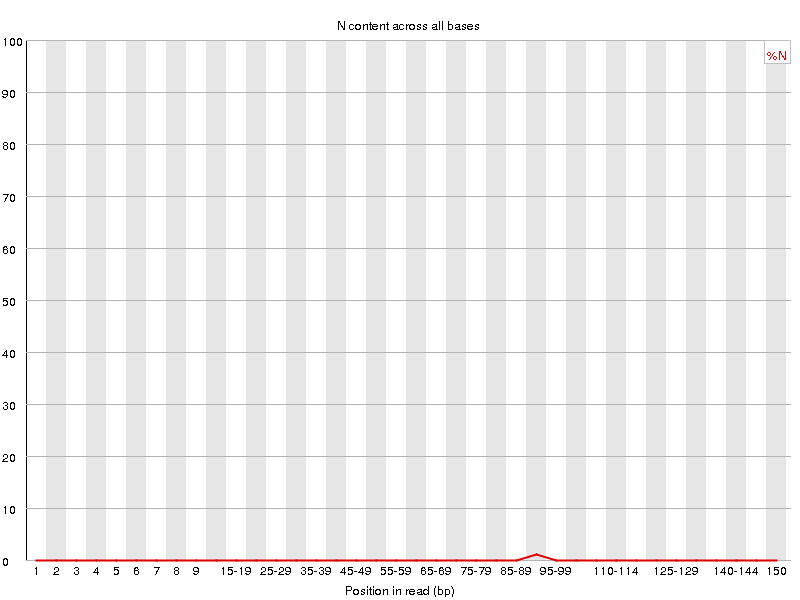

Supplement: Supplementary file 1 [file DataSheet_1.zip › sequencing/025G-201090513-CX-116_│┬╨π_6╚╦╤∙▒╛lncRNA_20190627/025G-201090513-CX-116_chenxiu_6╚╦╤∙▒╛lncRNA_20190627/1-Quality/clean/A2_clean_R2_fastqc/Images/per_base_n_content.png]

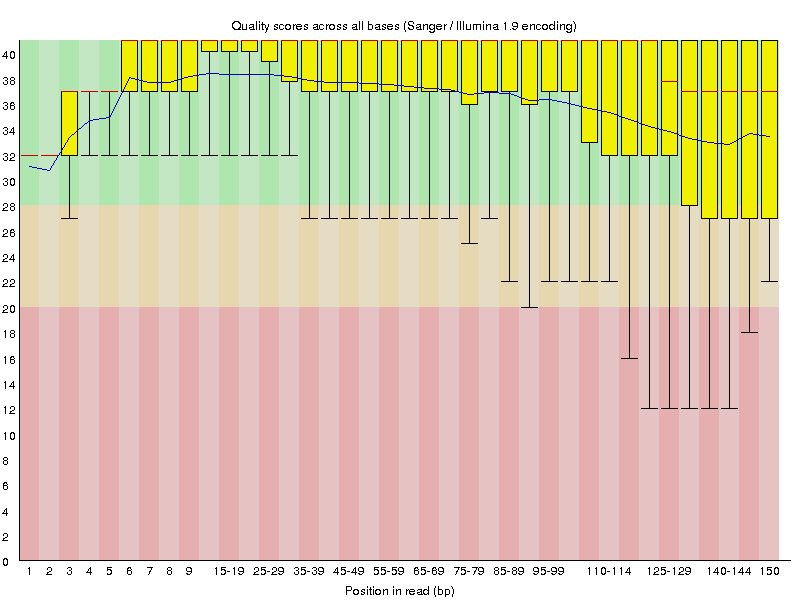

Supplement: Supplementary file 1 [file DataSheet_1.zip › sequencing/025G-201090513-CX-116_│┬╨π_6╚╦╤∙▒╛lncRNA_20190627/025G-201090513-CX-116_chenxiu_6╚╦╤∙▒╛lncRNA_20190627/1-Quality/clean/A2_clean_R2_fastqc/Images/per_base_quality.png]

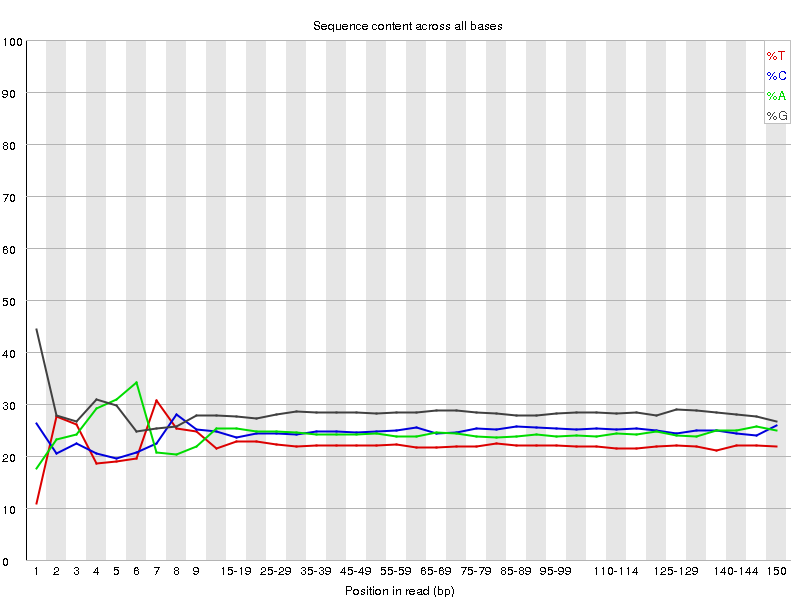

Supplement: Supplementary file 1 [file DataSheet_1.zip › sequencing/025G-201090513-CX-116_│┬╨π_6╚╦╤∙▒╛lncRNA_20190627/025G-201090513-CX-116_chenxiu_6╚╦╤∙▒╛lncRNA_20190627/1-Quality/clean/A2_clean_R2_fastqc/Images/per_base_sequence_content.png]

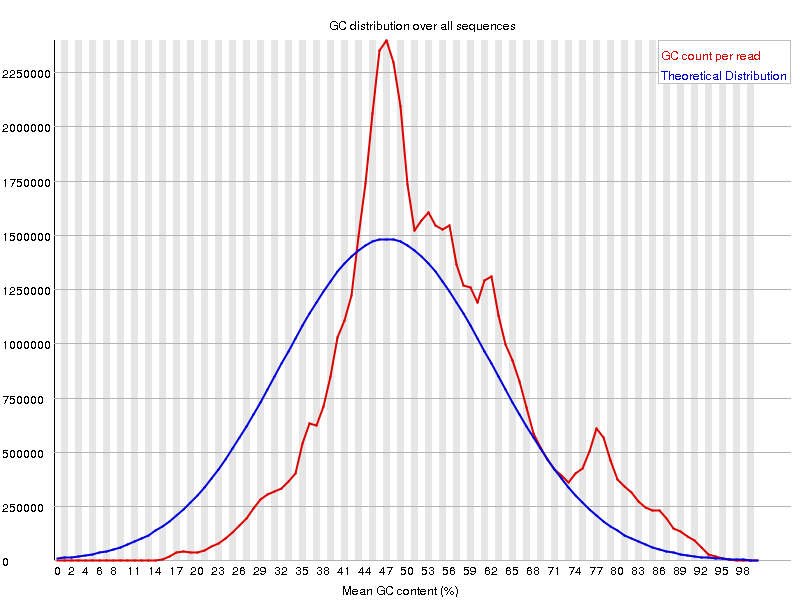

Supplement: Supplementary file 1 [file DataSheet_1.zip › sequencing/025G-201090513-CX-116_│┬╨π_6╚╦╤∙▒╛lncRNA_20190627/025G-201090513-CX-116_chenxiu_6╚╦╤∙▒╛lncRNA_20190627/1-Quality/clean/A2_clean_R2_fastqc/Images/per_sequence_gc_content.png]

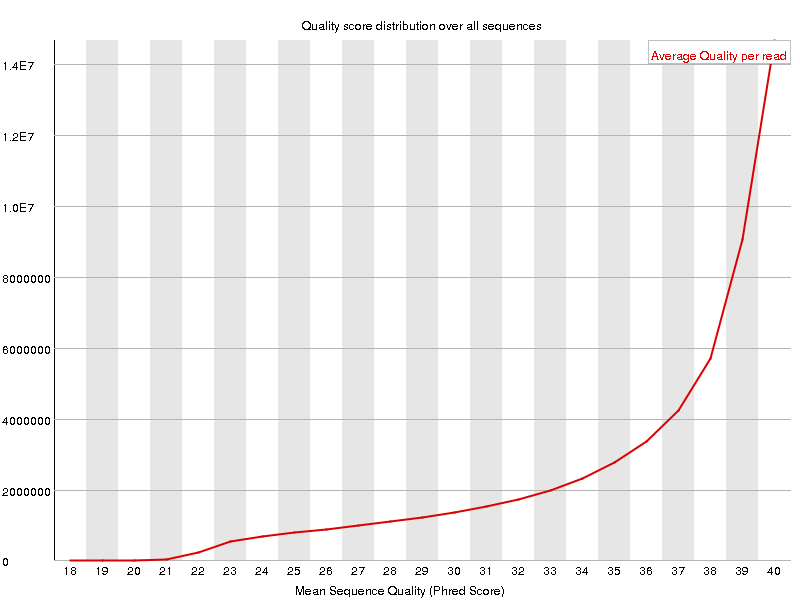

Supplement: Supplementary file 1 [file DataSheet_1.zip › sequencing/025G-201090513-CX-116_│┬╨π_6╚╦╤∙▒╛lncRNA_20190627/025G-201090513-CX-116_chenxiu_6╚╦╤∙▒╛lncRNA_20190627/1-Quality/clean/A2_clean_R2_fastqc/Images/per_sequence_quality.png]

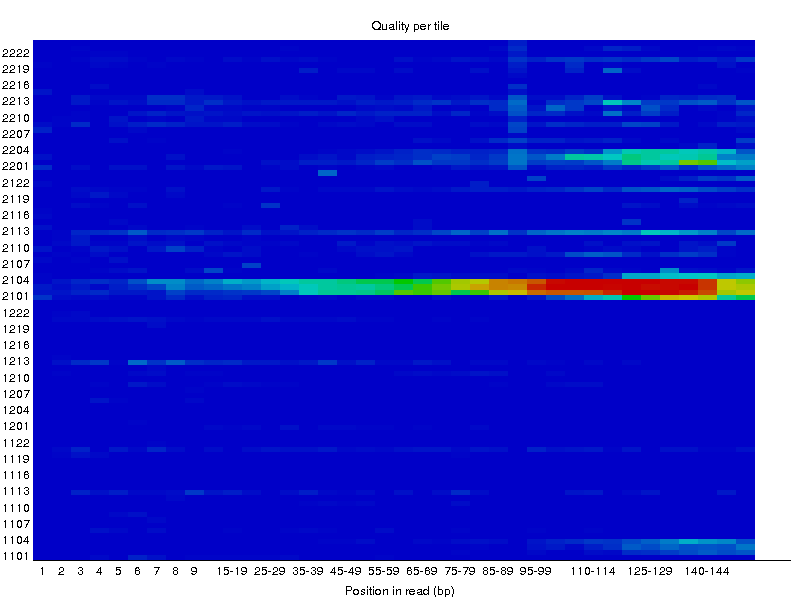

Supplement: Supplementary file 1 [file DataSheet_1.zip › sequencing/025G-201090513-CX-116_│┬╨π_6╚╦╤∙▒╛lncRNA_20190627/025G-201090513-CX-116_chenxiu_6╚╦╤∙▒╛lncRNA_20190627/1-Quality/clean/A2_clean_R2_fastqc/Images/per_tile_quality.png]

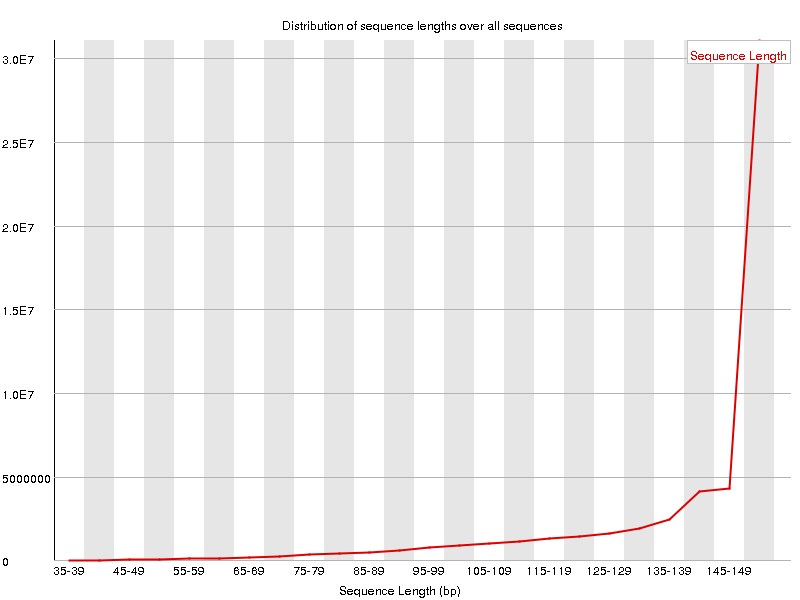

Supplement: Supplementary file 1 [file DataSheet_1.zip › sequencing/025G-201090513-CX-116_│┬╨π_6╚╦╤∙▒╛lncRNA_20190627/025G-201090513-CX-116_chenxiu_6╚╦╤∙▒╛lncRNA_20190627/1-Quality/clean/A2_clean_R2_fastqc/Images/sequence_length_distribution.png]

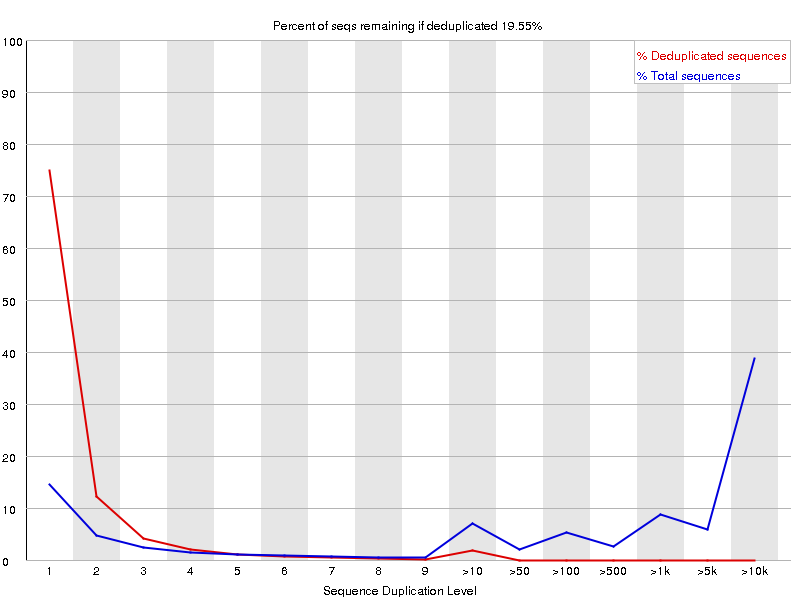

Supplement: Supplementary file 1 [file DataSheet_1.zip › sequencing/025G-201090513-CX-116_│┬╨π_6╚╦╤∙▒╛lncRNA_20190627/025G-201090513-CX-116_chenxiu_6╚╦╤∙▒╛lncRNA_20190627/1-Quality/clean/A3_clean_R1_fastqc/Images/duplication_levels.png]

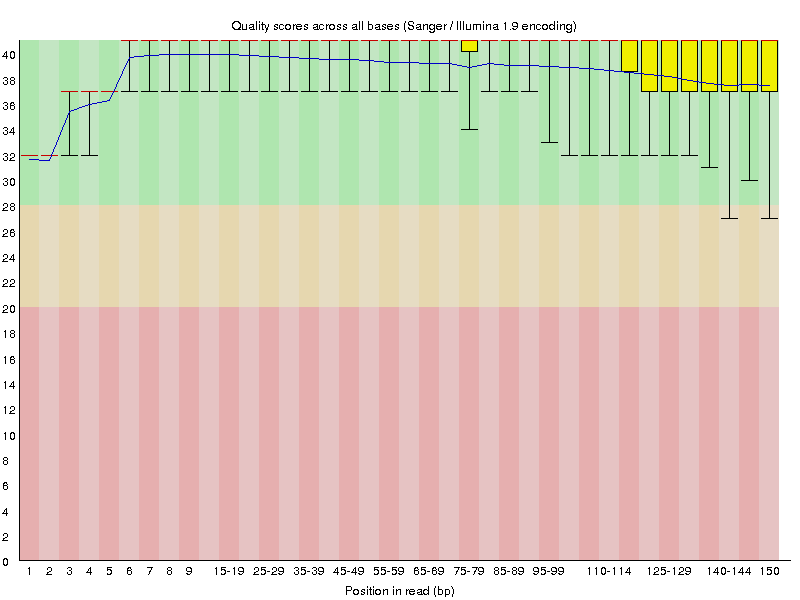

Supplement: Supplementary file 1 [file DataSheet_1.zip › sequencing/025G-201090513-CX-116_│┬╨π_6╚╦╤∙▒╛lncRNA_20190627/025G-201090513-CX-116_chenxiu_6╚╦╤∙▒╛lncRNA_20190627/1-Quality/clean/A3_clean_R1_fastqc/Images/per_base_quality.png]

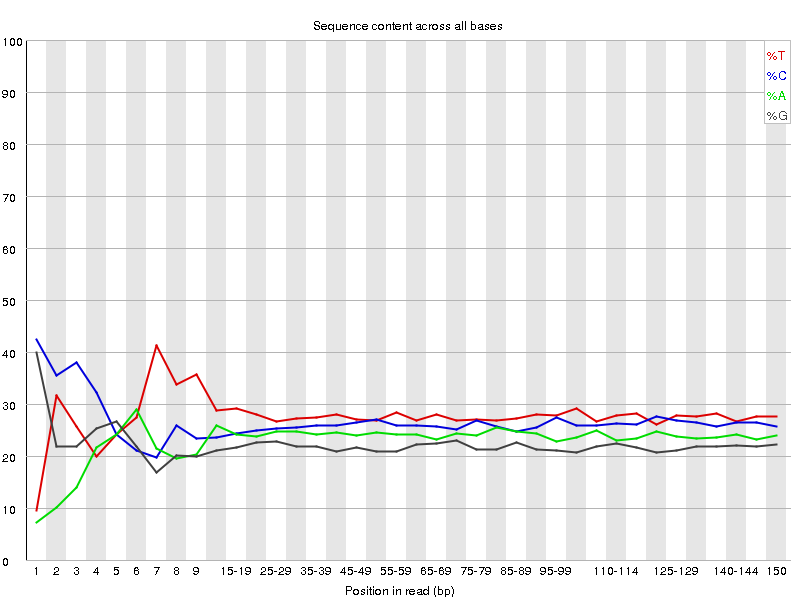

Supplement: Supplementary file 1 [file DataSheet_1.zip › sequencing/025G-201090513-CX-116_│┬╨π_6╚╦╤∙▒╛lncRNA_20190627/025G-201090513-CX-116_chenxiu_6╚╦╤∙▒╛lncRNA_20190627/1-Quality/clean/A3_clean_R1_fastqc/Images/per_base_sequence_content.png]

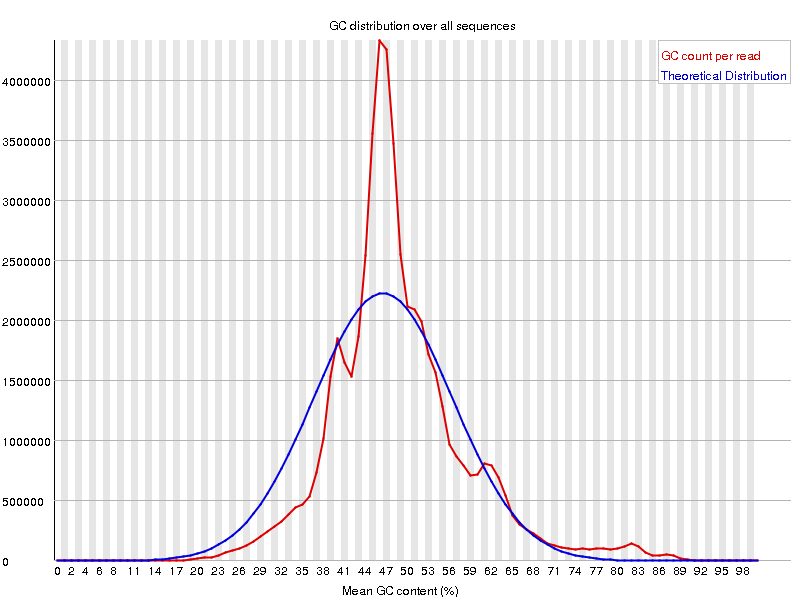

Supplement: Supplementary file 1 [file DataSheet_1.zip › sequencing/025G-201090513-CX-116_│┬╨π_6╚╦╤∙▒╛lncRNA_20190627/025G-201090513-CX-116_chenxiu_6╚╦╤∙▒╛lncRNA_20190627/1-Quality/clean/A3_clean_R1_fastqc/Images/per_sequence_gc_content.png]

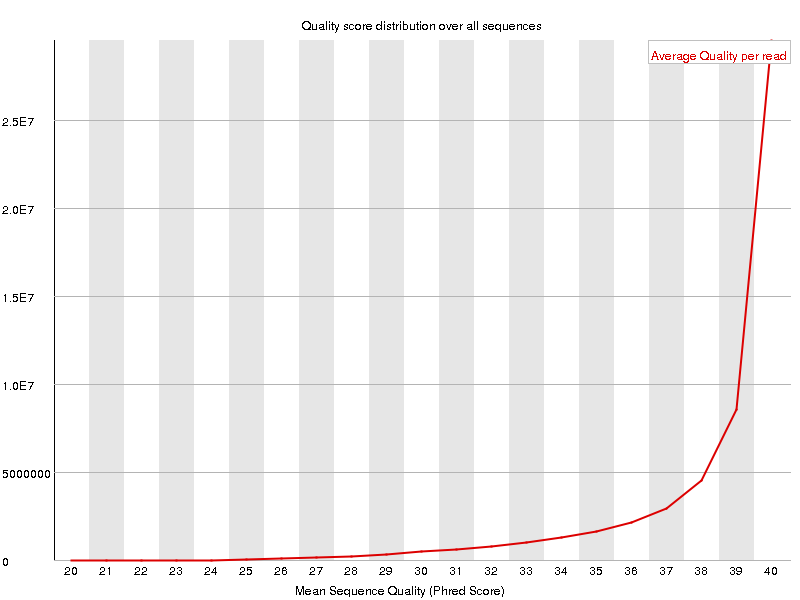

Supplement: Supplementary file 1 [file DataSheet_1.zip › sequencing/025G-201090513-CX-116_│┬╨π_6╚╦╤∙▒╛lncRNA_20190627/025G-201090513-CX-116_chenxiu_6╚╦╤∙▒╛lncRNA_20190627/1-Quality/clean/A3_clean_R1_fastqc/Images/per_sequence_quality.png]

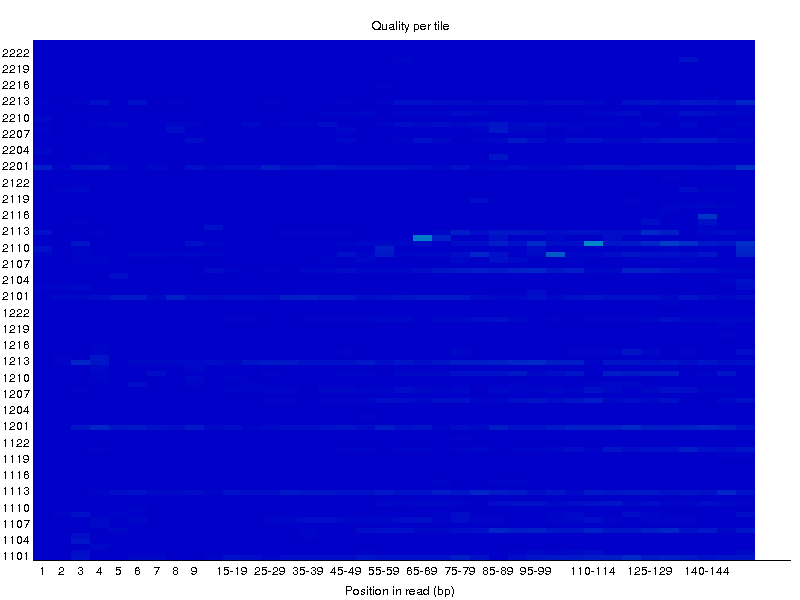

Supplement: Supplementary file 1 [file DataSheet_1.zip › sequencing/025G-201090513-CX-116_│┬╨π_6╚╦╤∙▒╛lncRNA_20190627/025G-201090513-CX-116_chenxiu_6╚╦╤∙▒╛lncRNA_20190627/1-Quality/clean/A3_clean_R1_fastqc/Images/per_tile_quality.png]

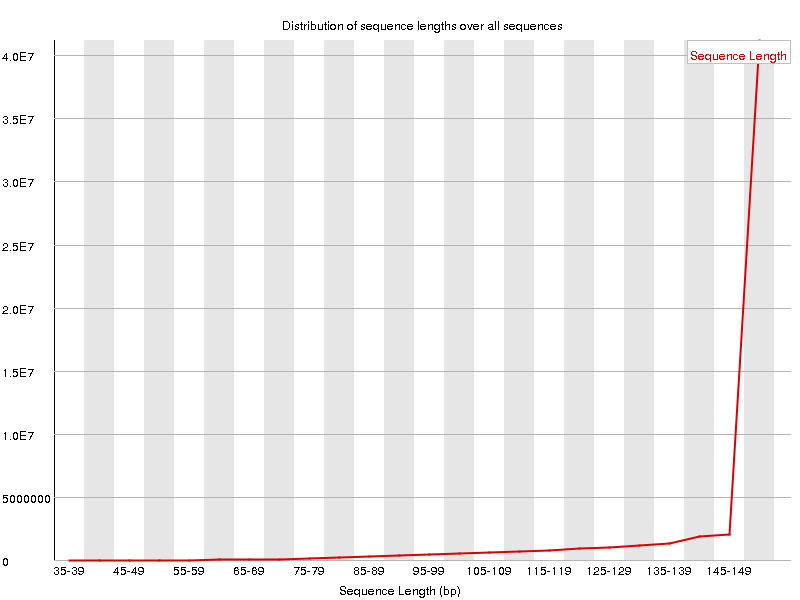

Supplement: Supplementary file 1 [file DataSheet_1.zip › sequencing/025G-201090513-CX-116_│┬╨π_6╚╦╤∙▒╛lncRNA_20190627/025G-201090513-CX-116_chenxiu_6╚╦╤∙▒╛lncRNA_20190627/1-Quality/clean/A3_clean_R1_fastqc/Images/sequence_length_distribution.png]

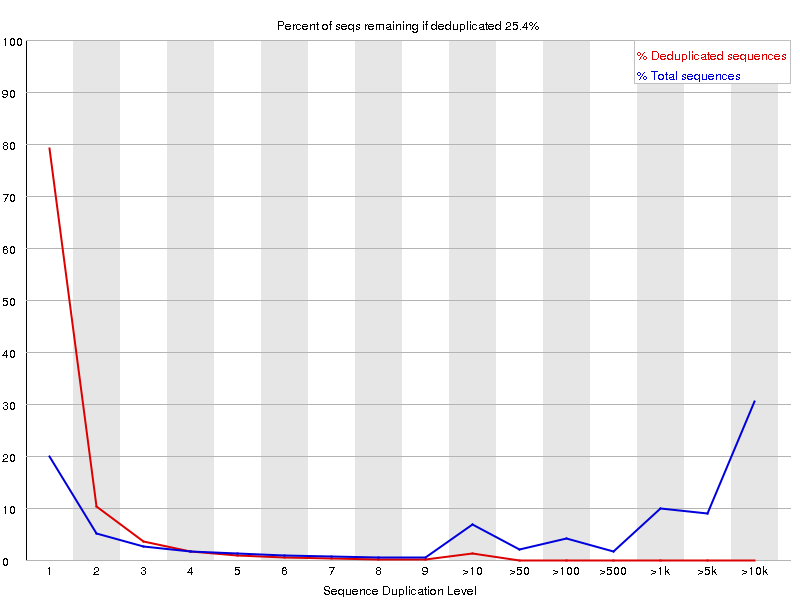

Supplement: Supplementary file 1 [file DataSheet_1.zip › sequencing/025G-201090513-CX-116_│┬╨π_6╚╦╤∙▒╛lncRNA_20190627/025G-201090513-CX-116_chenxiu_6╚╦╤∙▒╛lncRNA_20190627/1-Quality/clean/A3_clean_R2_fastqc/Images/duplication_levels.png]

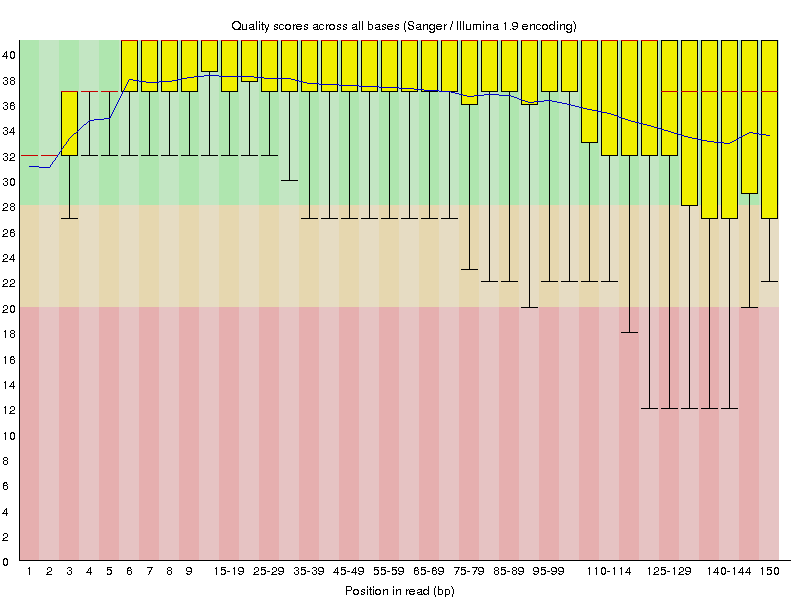

Supplement: Supplementary file 1 [file DataSheet_1.zip › sequencing/025G-201090513-CX-116_│┬╨π_6╚╦╤∙▒╛lncRNA_20190627/025G-201090513-CX-116_chenxiu_6╚╦╤∙▒╛lncRNA_20190627/1-Quality/clean/A3_clean_R2_fastqc/Images/per_base_quality.png]

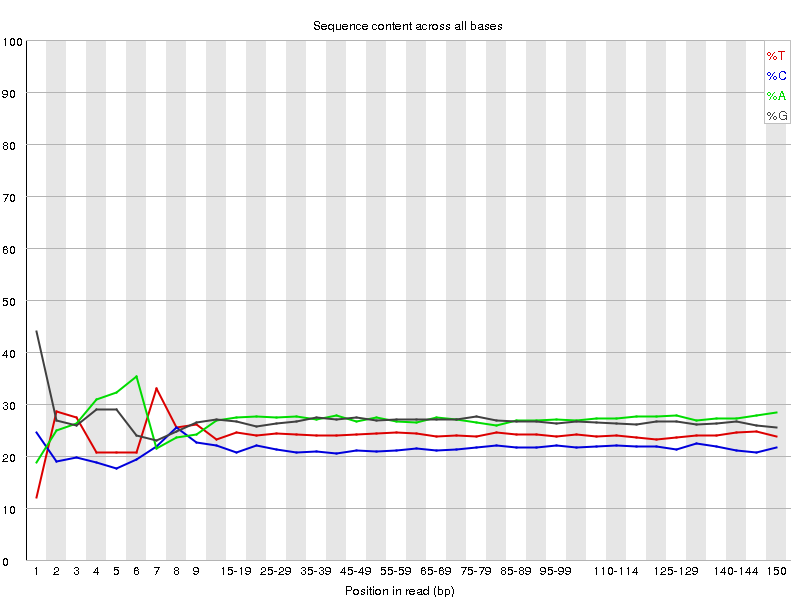

Supplement: Supplementary file 1 [file DataSheet_1.zip › sequencing/025G-201090513-CX-116_│┬╨π_6╚╦╤∙▒╛lncRNA_20190627/025G-201090513-CX-116_chenxiu_6╚╦╤∙▒╛lncRNA_20190627/1-Quality/clean/A3_clean_R2_fastqc/Images/per_base_sequence_content.png]

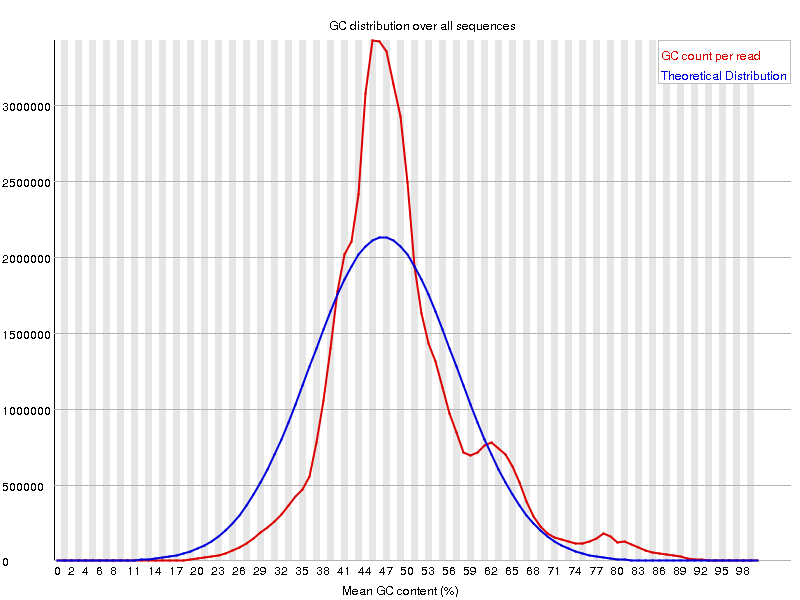

Supplement: Supplementary file 1 [file DataSheet_1.zip › sequencing/025G-201090513-CX-116_│┬╨π_6╚╦╤∙▒╛lncRNA_20190627/025G-201090513-CX-116_chenxiu_6╚╦╤∙▒╛lncRNA_20190627/1-Quality/clean/A3_clean_R2_fastqc/Images/per_sequence_gc_content.png]

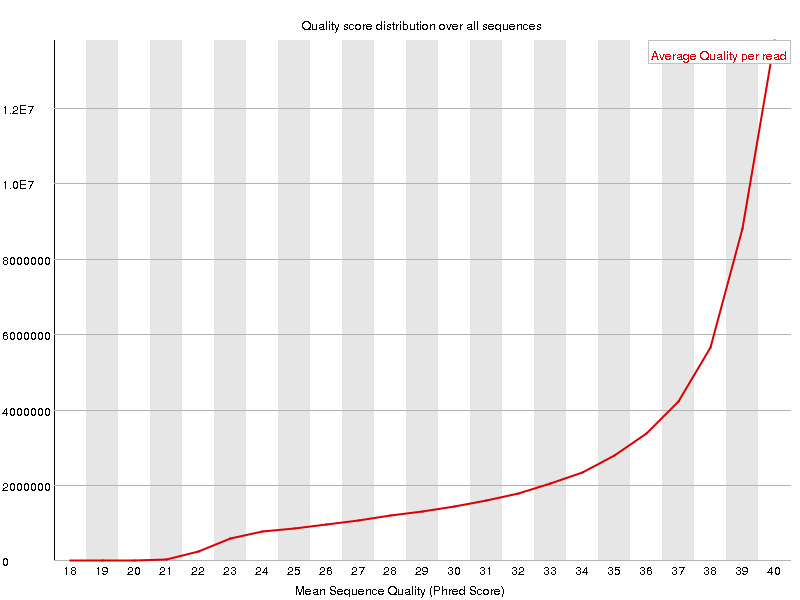

Supplement: Supplementary file 1 [file DataSheet_1.zip › sequencing/025G-201090513-CX-116_│┬╨π_6╚╦╤∙▒╛lncRNA_20190627/025G-201090513-CX-116_chenxiu_6╚╦╤∙▒╛lncRNA_20190627/1-Quality/clean/A3_clean_R2_fastqc/Images/per_sequence_quality.png]

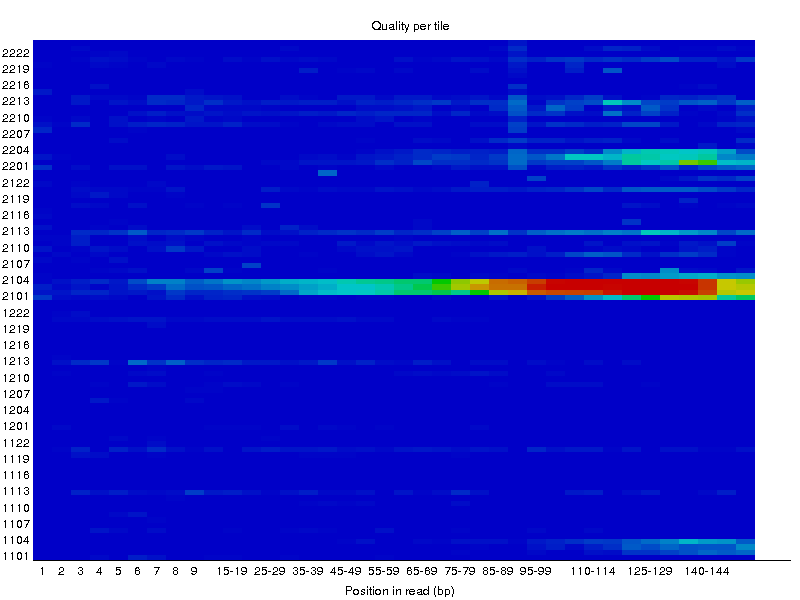

Supplement: Supplementary file 1 [file DataSheet_1.zip › sequencing/025G-201090513-CX-116_│┬╨π_6╚╦╤∙▒╛lncRNA_20190627/025G-201090513-CX-116_chenxiu_6╚╦╤∙▒╛lncRNA_20190627/1-Quality/clean/A3_clean_R2_fastqc/Images/per_tile_quality.png]

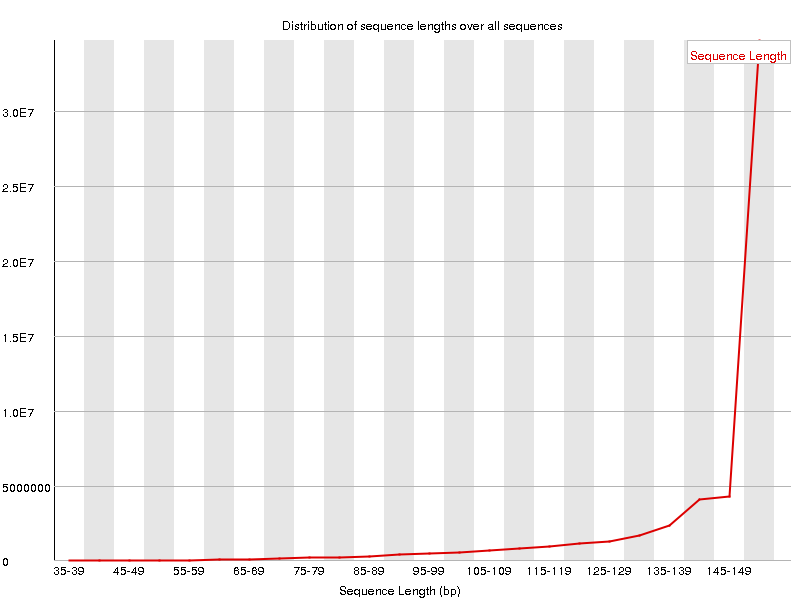

Supplement: Supplementary file 1 [file DataSheet_1.zip › sequencing/025G-201090513-CX-116_│┬╨π_6╚╦╤∙▒╛lncRNA_20190627/025G-201090513-CX-116_chenxiu_6╚╦╤∙▒╛lncRNA_20190627/1-Quality/clean/A3_clean_R2_fastqc/Images/sequence_length_distribution.png]

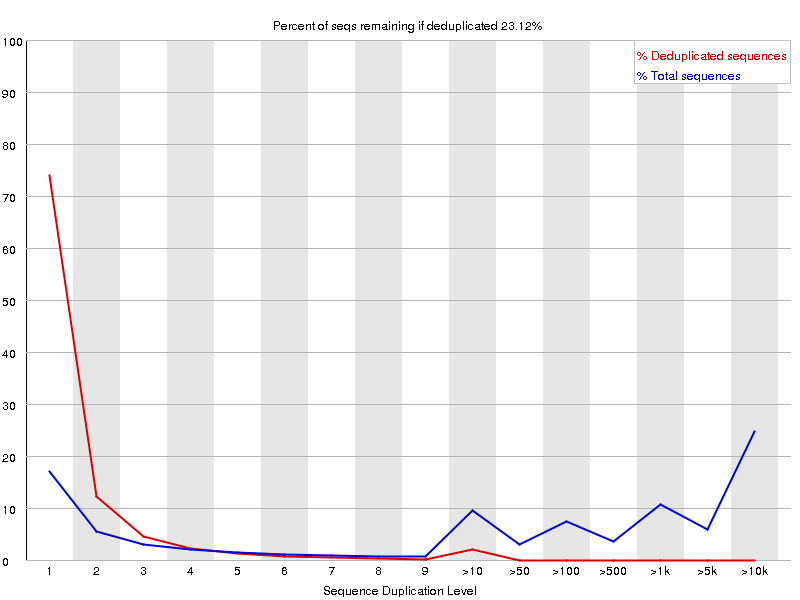

Supplement: Supplementary file 1 [file DataSheet_1.zip › sequencing/025G-201090513-CX-116_│┬╨π_6╚╦╤∙▒╛lncRNA_20190627/025G-201090513-CX-116_chenxiu_6╚╦╤∙▒╛lncRNA_20190627/1-Quality/clean/B1_clean_R1_fastqc/Images/duplication_levels.png]

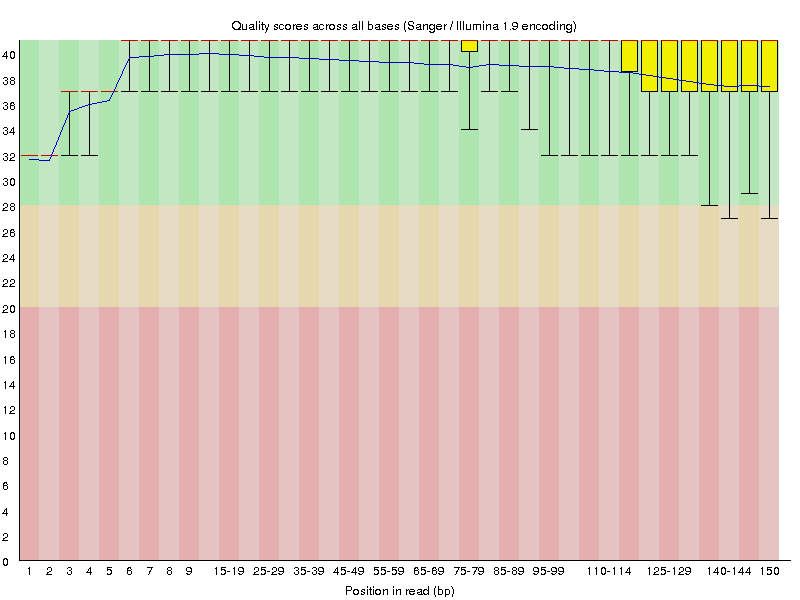

Supplement: Supplementary file 1 [file DataSheet_1.zip › sequencing/025G-201090513-CX-116_│┬╨π_6╚╦╤∙▒╛lncRNA_20190627/025G-201090513-CX-116_chenxiu_6╚╦╤∙▒╛lncRNA_20190627/1-Quality/clean/B1_clean_R1_fastqc/Images/per_base_quality.png]

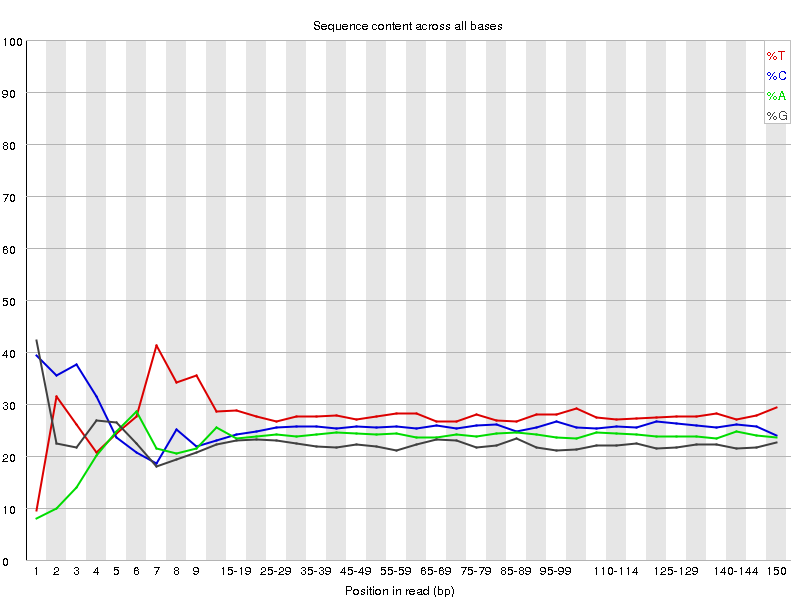

Supplement: Supplementary file 1 [file DataSheet_1.zip › sequencing/025G-201090513-CX-116_│┬╨π_6╚╦╤∙▒╛lncRNA_20190627/025G-201090513-CX-116_chenxiu_6╚╦╤∙▒╛lncRNA_20190627/1-Quality/clean/B1_clean_R1_fastqc/Images/per_base_sequence_content.png]

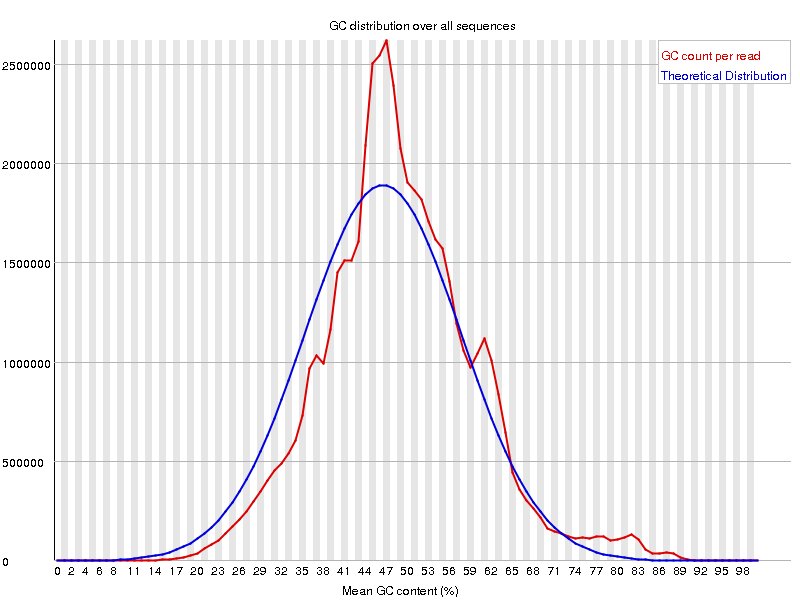

Supplement: Supplementary file 1 [file DataSheet_1.zip › sequencing/025G-201090513-CX-116_│┬╨π_6╚╦╤∙▒╛lncRNA_20190627/025G-201090513-CX-116_chenxiu_6╚╦╤∙▒╛lncRNA_20190627/1-Quality/clean/B1_clean_R1_fastqc/Images/per_sequence_gc_content.png]

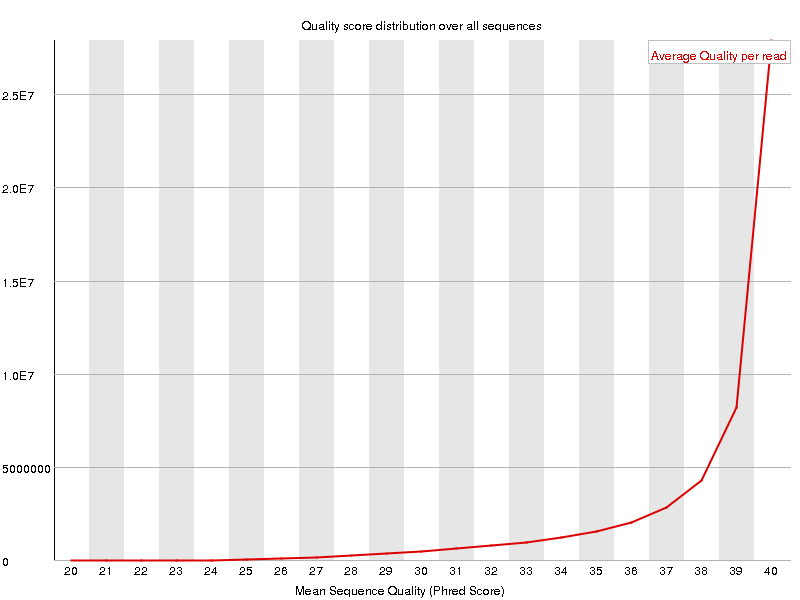

Supplement: Supplementary file 1 [file DataSheet_1.zip › sequencing/025G-201090513-CX-116_│┬╨π_6╚╦╤∙▒╛lncRNA_20190627/025G-201090513-CX-116_chenxiu_6╚╦╤∙▒╛lncRNA_20190627/1-Quality/clean/B1_clean_R1_fastqc/Images/per_sequence_quality.png]

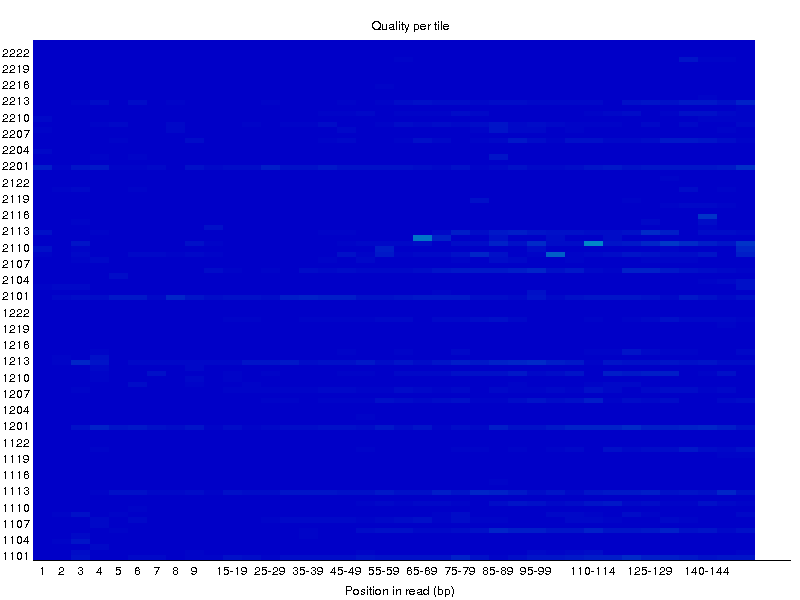

Supplement: Supplementary file 1 [file DataSheet_1.zip › sequencing/025G-201090513-CX-116_│┬╨π_6╚╦╤∙▒╛lncRNA_20190627/025G-201090513-CX-116_chenxiu_6╚╦╤∙▒╛lncRNA_20190627/1-Quality/clean/B1_clean_R1_fastqc/Images/per_tile_quality.png]

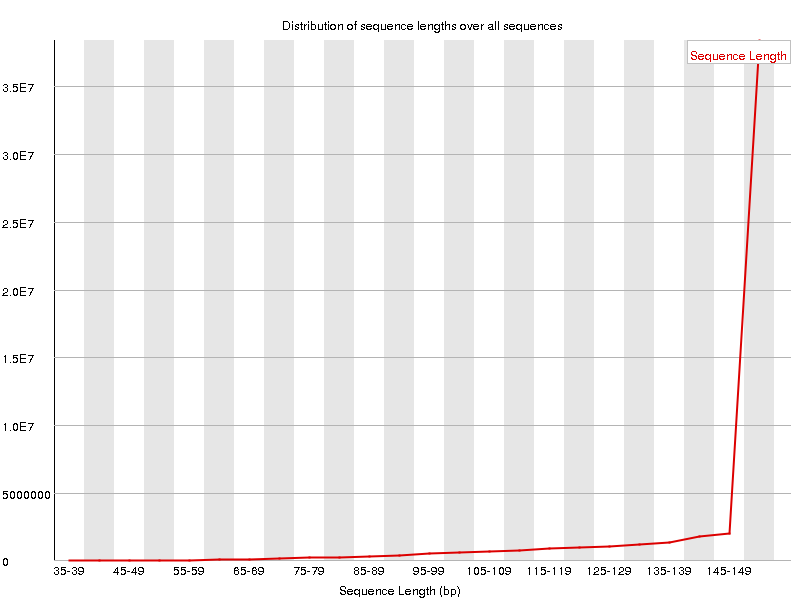

Supplement: Supplementary file 1 [file DataSheet_1.zip › sequencing/025G-201090513-CX-116_│┬╨π_6╚╦╤∙▒╛lncRNA_20190627/025G-201090513-CX-116_chenxiu_6╚╦╤∙▒╛lncRNA_20190627/1-Quality/clean/B1_clean_R1_fastqc/Images/sequence_length_distribution.png]
